# Supplementary material for: Simultaneous Quantification of 66 Compounds in Two Tibetan Codonopsis Species Reveals Four Chemical Features by Database-Enabled UHPLC-Q-Orbitrap-MS/MS Analysis
Source: Molecules. 2024 Nov 3;29(21):5203. doi: 10.3390/molecules29215203 (PMC11547486; doi:10.3390/molecules29215203)
Supplement: Supplementary file 1 [file molecules-29-05203-s001.zip › Supplementary data S1. UHPLC-Q-Orbitrap-MSMS spectra and identification of 66.pdf]

*Suppl. 1.1* Identification of betaine (CAS 107-43-7, $C_5H_{11}NO_2$, M.W. 117.15).

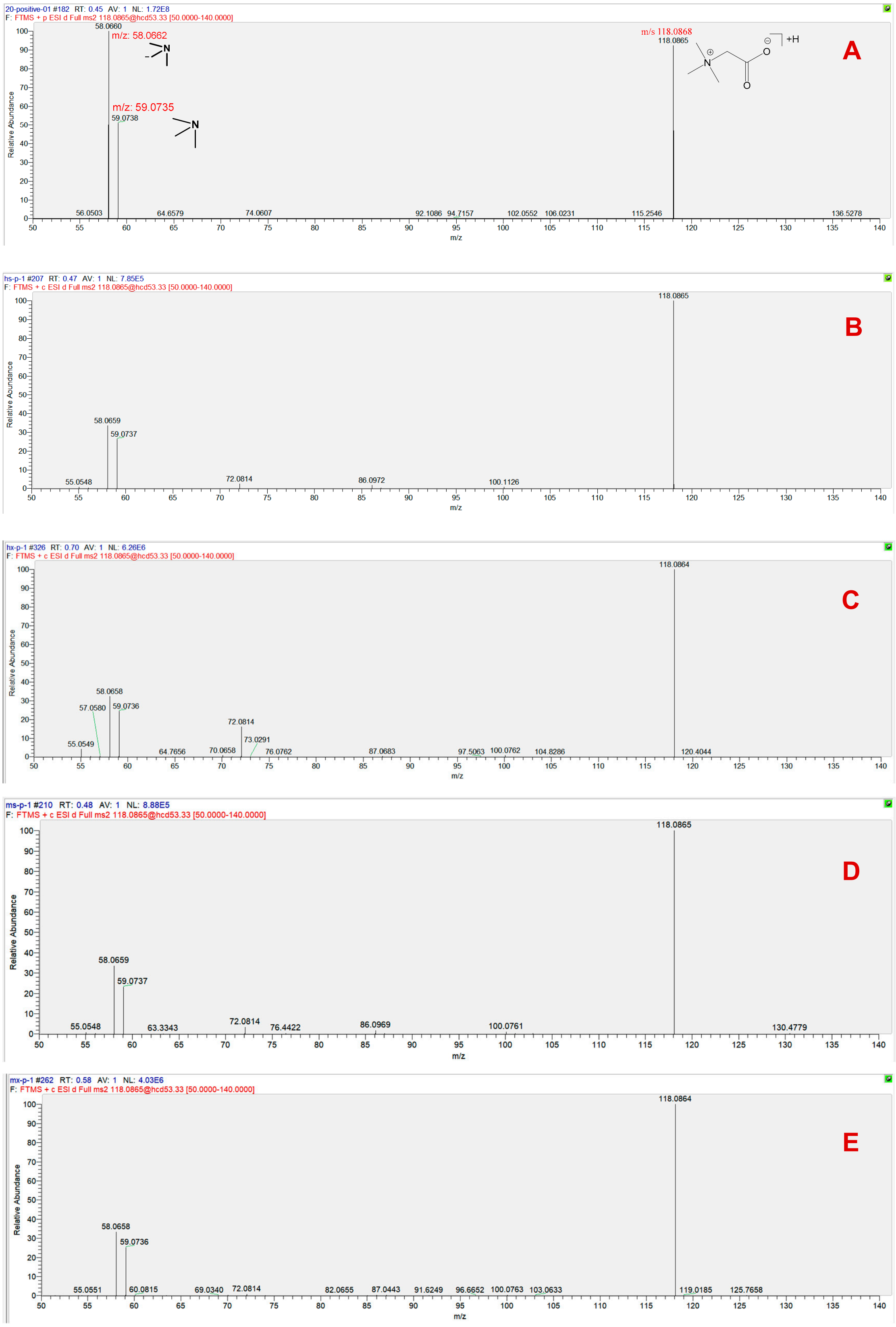


Fig. S1.1.1 The main results of betaine (CAS 107-43-7, $C_5H_{11}NO_2$) and its corresponding peak in the TIC diagram using UHPLC-Q-Orbitrap-MS/MS analysis. (**A**) The MS/MS fragments of authentic standard betaine. (**B**) The MS/MS spectra from chromatographic peak in the CoCA extract. (**C**) The

MS/MS spectra from chromatographic peak in the CoCU extract. (**D**) The MS/MS spectra from chromatographic peak in the CoNA extract. (**E**) The MS/MS spectra from chromatographic peak in the CoNU extract.

**Note**: The m/z values in red are the calculated ones. The m/z calculation was based on the relative atomic masses of C (12.0000), H (1.007825), O (15.994915), and N (14.003074)[1]

**Identification**: As seen in Fig. S1.1.1, the extract ion peak, MS/MS spectra, and characteristic pears were highly similar. Thus, the chromatographic peaks in the extracts (CoCA, CoCU, CoNA, CoNU) were identified as betaine (CAS 107-43-7).

*Suppl. 1.2* Identification of sucrose (CAS 57-50-1, $C_{12}H_{22}O_{11}$, M.W. 342.3 ).

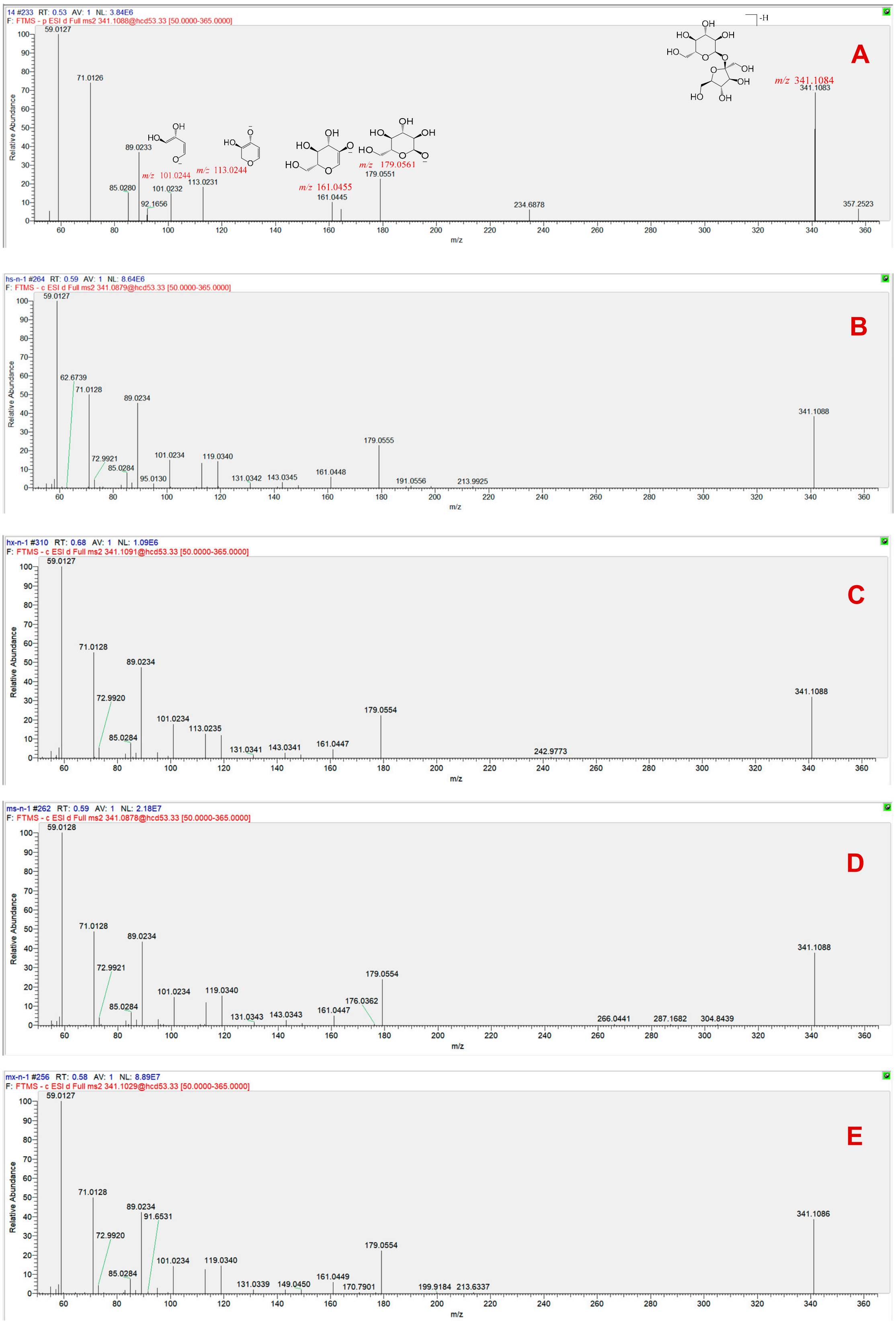


Fig. S1.2.1 The main results of sucrose (CAS 57-50-1, $C_{12}H_{22}O_{11}$) and its corresponding peak in the TIC diagram using UHPLC-Q-Orbitrap-MS/MS analysis. (**A**) The MS/MS fragments of authentic standard sucrose. (**B**) The MS/MS spectra from chromatographic peak in the CoCA extract. (**C**) The

MS/MS spectra from chromatographic peak in the CoCU extract. (**D**) The MS/MS spectra from chromatographic peak in the CoNA extract. (**E**) The MS/MS spectra from chromatographic peak in the CoNU extract.

**Note**: The m/z values in red are the calculated ones. The m/z calculation was based on the relative atomic masses of C (12.0000), H (1.007825), O (15.994915)[1]

**Identification**: As seen in Fig. S1.2.1, the extract ion peak, MS/MS spectra, and characteristic pears were highly similar. Thus, the chromatographic peaks in the extracts (CoCA, CoCU, CoNA, CoNU) were identified as sucrose (CAS 57-50-1).

## *Suppl. 1.3* Identification of D-gluconic acid (CAS 526-95-4, $C_6H_{12}O_7$, M.W. 196.16 ).

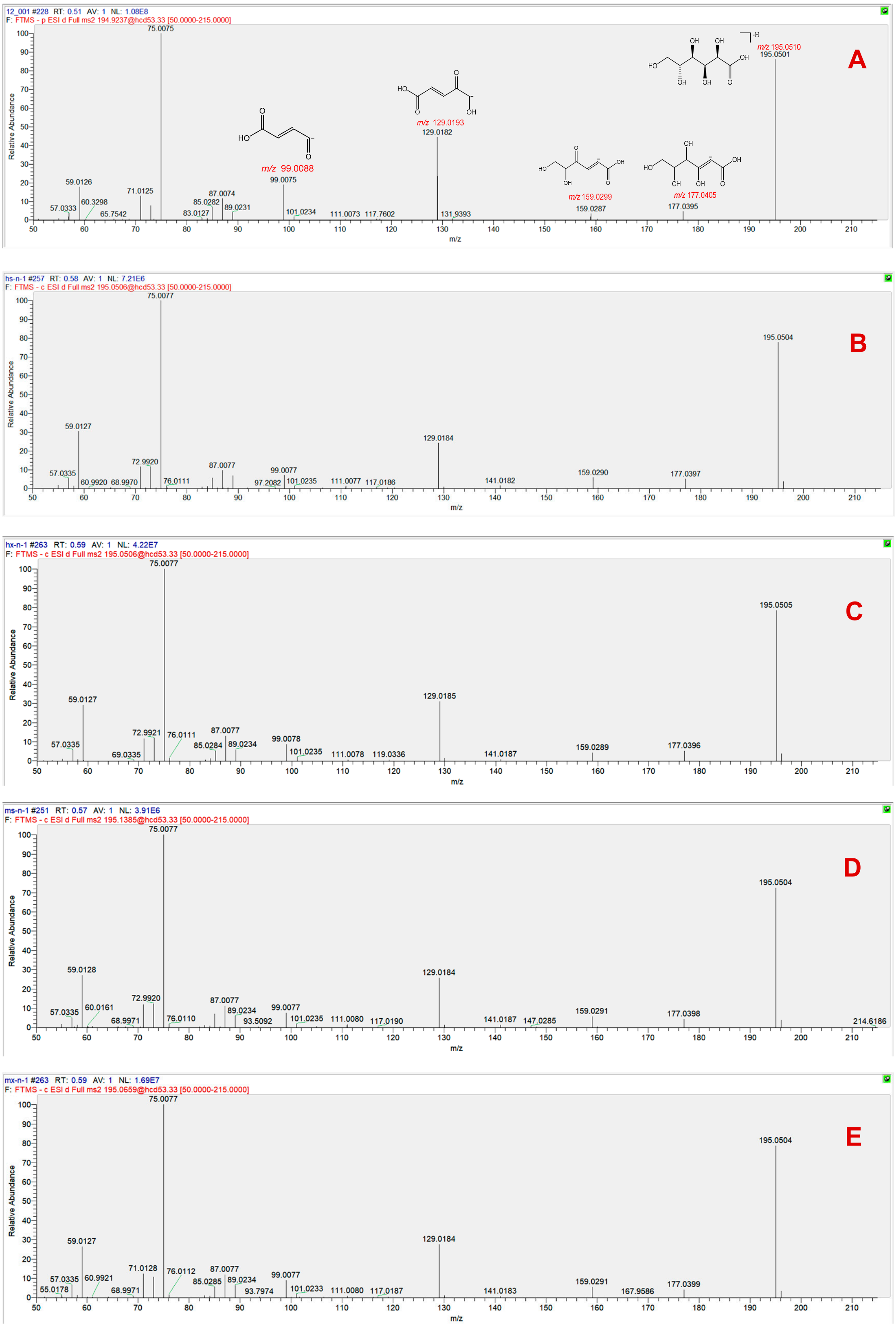


Fig. S1.3.1 The main results of D-gluconic acid (CAS 526-95-4, $C_6H_{12}O_7$) and its corresponding peak in the TIC diagram using UHPLC-Q-Orbitrap-MS/MS analysis. (**A**) The MS/MS fragments of authentic standard D-gluconic acid. (**B**) The MS/MS spectra from chromatographic peak in the CoCA

extract. (**C**) The MS/MS spectra from chromatographic peak in the CoCU extract. (**D**) The MS/MS spectra from chromatographic peak in the CoNA extract. (**E**) The MS/MS spectra from chromatographic peak in the CoNU extract.

**Note**: The m/z values in red are the calculated ones. The m/z calculation was based on the relative atomic masses of C (12.0000), H (1.007825), O (15.994915)[1]

**Identification**: As seen in Fig. S1.3.1, the extract ion peak, MS/MS spectra, and characteristic pears were highly similar. Thus, the chromatographic peaks in the extracts (CoCA, CoCU, CoNA, CoNU) were identified as D-gluconic acid (CAS 526-95-4).

## *Suppl. 1.4* Identification of 1-kestose (CAS 470-69-9, $C_{18}H_{32}O_{16}$, M.W. 504.44).

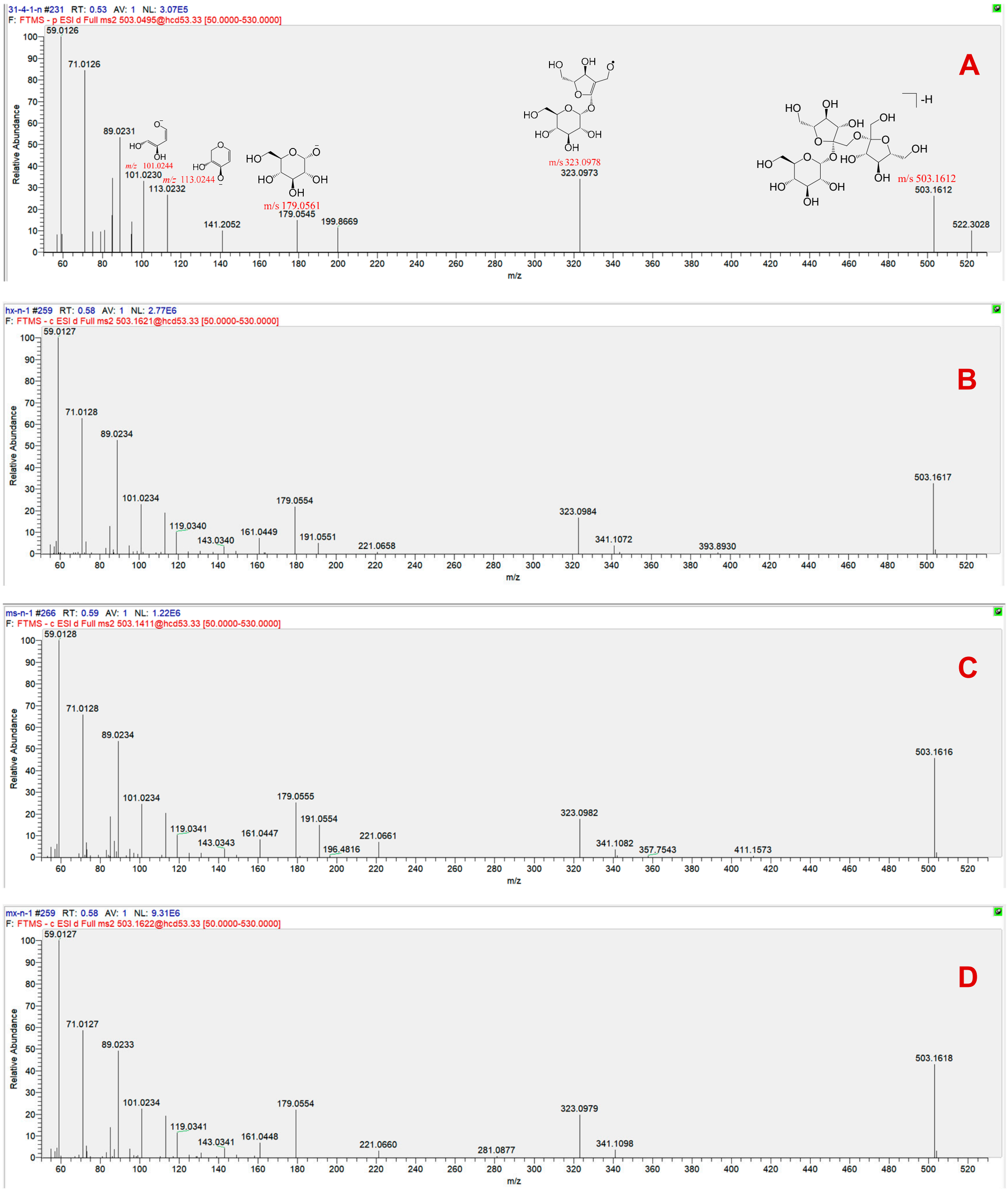


Fig. S1.4.1 The main results of 1-kestose (CAS 470-69-9, $C_{18}H_{32}O_{16}$) and its corresponding peak in the TIC diagram using UHPLC-Q-Orbitrap-MS/MS analysis. (**A**) The MS/MS fragments of authentic standard 1-kestose. (**B**) The MS/MS spectra from chromatographic peak in the CoCU extract. (**C**) The MS/MS spectra from chromatographic peak in the CoNA extract. (**D**) The MS/MS spectra from chromatographic peak in the CoNU extract.

**Note**: The m/z values in red are the calculated ones. The m/z calculation was based on the relative atomic masses of C (12.0000), H (1.007825), O (15.994915), and N (14.003074)[1]

**Identification**: As seen in Fig. S1.4.1, the extract ion peak, MS/MS spectra, and characteristic pears were highly similar. Thus, the chromatographic peaks in the extracts (CoCU, CoNA, CoNU) were identified as 1-kestose (CAS 470-69-9).

*Suppl. 1.5* Identification of *L*-proline (CAS 147-85-3, $C_5H_9NO_2$, M.W. 115.13 ).

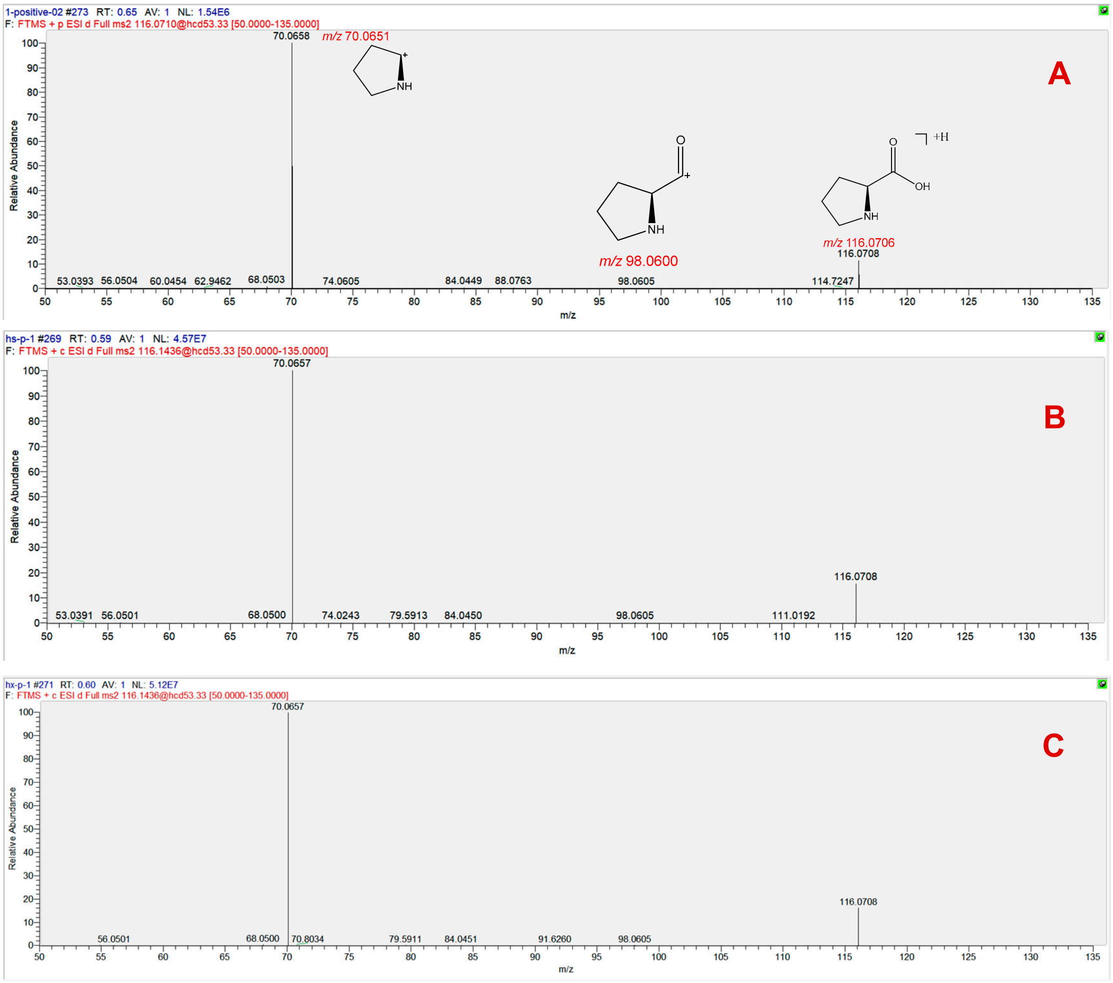


Fig. S1.5.1 The main results of *L*-proline (CAS 147-85-3, $C_5H_9NO_2$) and its corresponding peak in the TIC diagram using UHPLC-Q-Orbitrap-MS/MS analysis. (**A**) The MS/MS fragments of authentic standard *L*-proline. (**B**) The MS/MS spectra from chromatographic peak in the CoCA extract. (**C**) The MS/MS spectra from chromatographic peak in the CoCU extract.

**Note**: The m/z values in red are the calculated ones. The m/z calculation was based on the relative atomic masses of C (12.0000), H (1.007825), O (15.994915), and N (14.003074)[1]

**Identification**: As seen in Fig. S1.5.1, the extract ion peak, MS/MS spectra, and characteristic pears were highly similar. Thus, the chromatographic peaks in the extracts (CoCA, CoCU) were identified as L-proline (CAS 147-85-3).

*Suppl. 1.6* Identification of nystose (CAS 13133-07-8, $C_{24}H_{42}O_{21}$, M.W. 666.58).

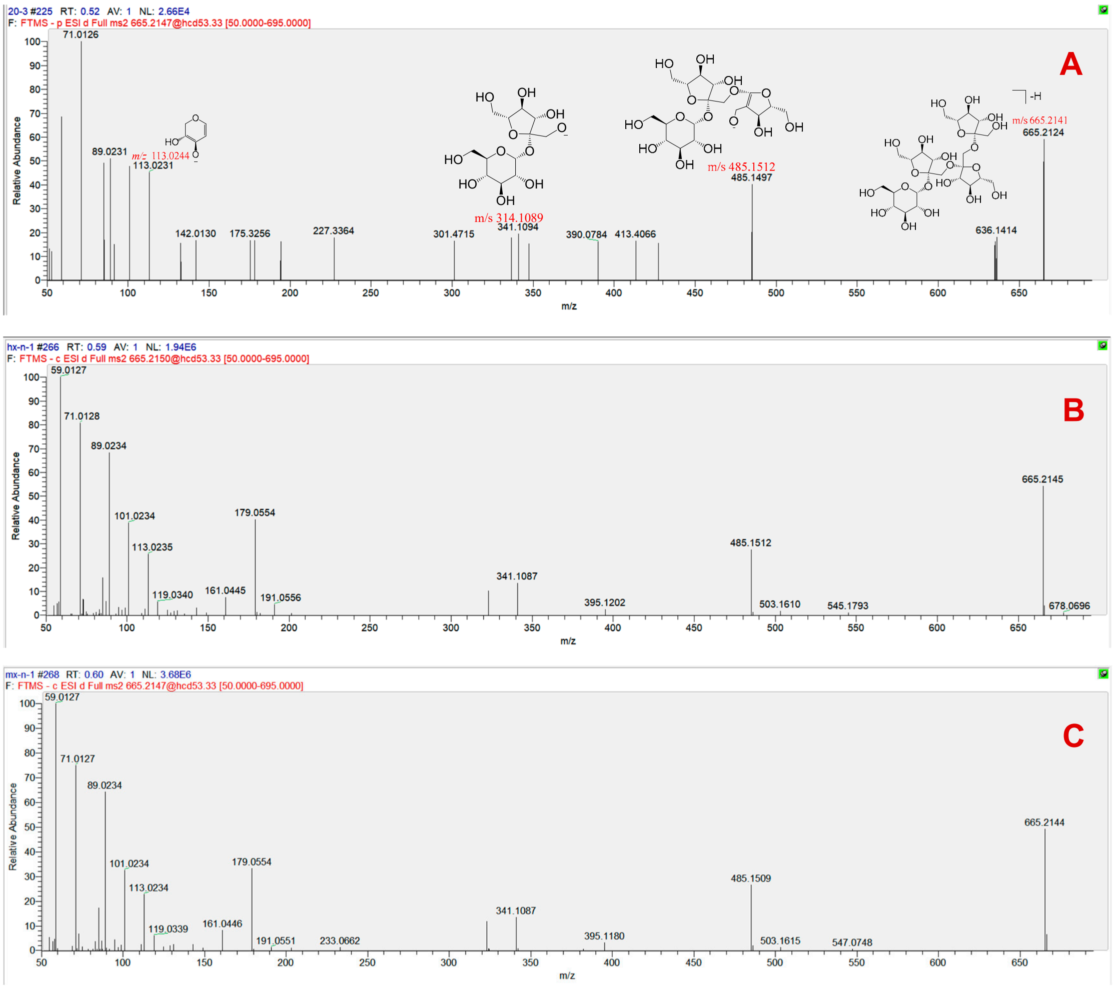


Fig. S1.6.1 The main results of nystose (CAS 13133-07-8, $C_{24}H_{42}O_{21}$) and its corresponding peak in the TIC diagram using UHPLC-Q-Orbitrap-MS/MS analysis. (**A**) The MS/MS fragments of authentic standard nystose. (**B**) The MS/MS spectra from chromatographic peak in the CoCU extract. (**C**) The MS/MS spectra from chromatographic peak in the CoNU extract.

**Note**: The m/z values in red are the calculated ones. The m/z calculation was based on the relative atomic masses of C (12.0000), H (1.007825), O (15.994915)[1]

**Identification**: As seen in Fig. S1.6.1, the extract ion peak, MS/MS spectra, and characteristic pears were highly similar. Thus, the chromatographic peaks in the extracts (CoCU, CoNU) were identified as nystose (CAS 13133-07-8).

## *Suppl. 1.7* Identification of quinic acid (CAS 77-95-2, $C_7H_{12}O_6$, M.W. 192.17 ).

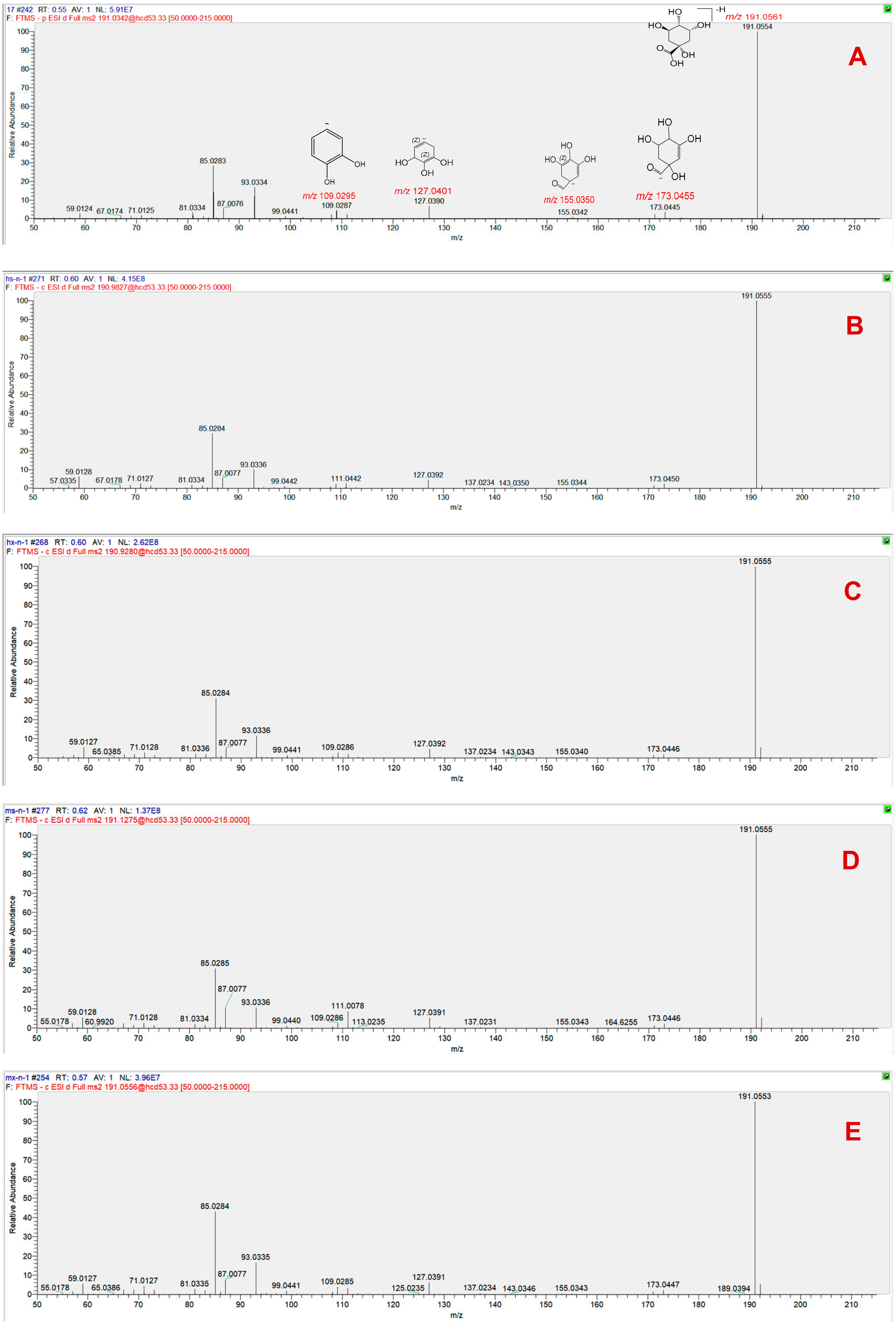


Fig. S1.7.1 The main results of quinic acid (CAS 77-95-2, $C_7H_{12}O_6$) and its corresponding peak in the TIC diagram using UHPLC-Q-Orbitrap-MS/MS analysis. (**A**) The MS/MS fragments of authentic standard quinic acid. (**B**) The MS/MS spectra from chromatographic peak in the CoCA extract. (**C**) The

MS/MS spectra from chromatographic peak in the CoCU extract. (**D**) The MS/MS spectra from chromatographic peak in the CoNA extract. (**E**) The MS/MS spectra from chromatographic peak in the CoNU extract.

**Note**: The m/z values in red are the calculated ones. The m/z calculation was based on the relative atomic masses of C (12.0000), H (1.007825), O (15.994915)[1]

**Identification**: As seen in Fig. S1.7.1, the extract ion peak, MS/MS spectra, and characteristic pears were highly similar. Thus, the chromatographic peaks in the extracts (CoCA, CoCU, CoNA, CoNU) were identified as quinic acid (CAS 77-95-2).

*Suppl. 1.8* Identification of malic acid (CAS 6915-15-7, $C_4H_6O_5$, M.W. 134.09).

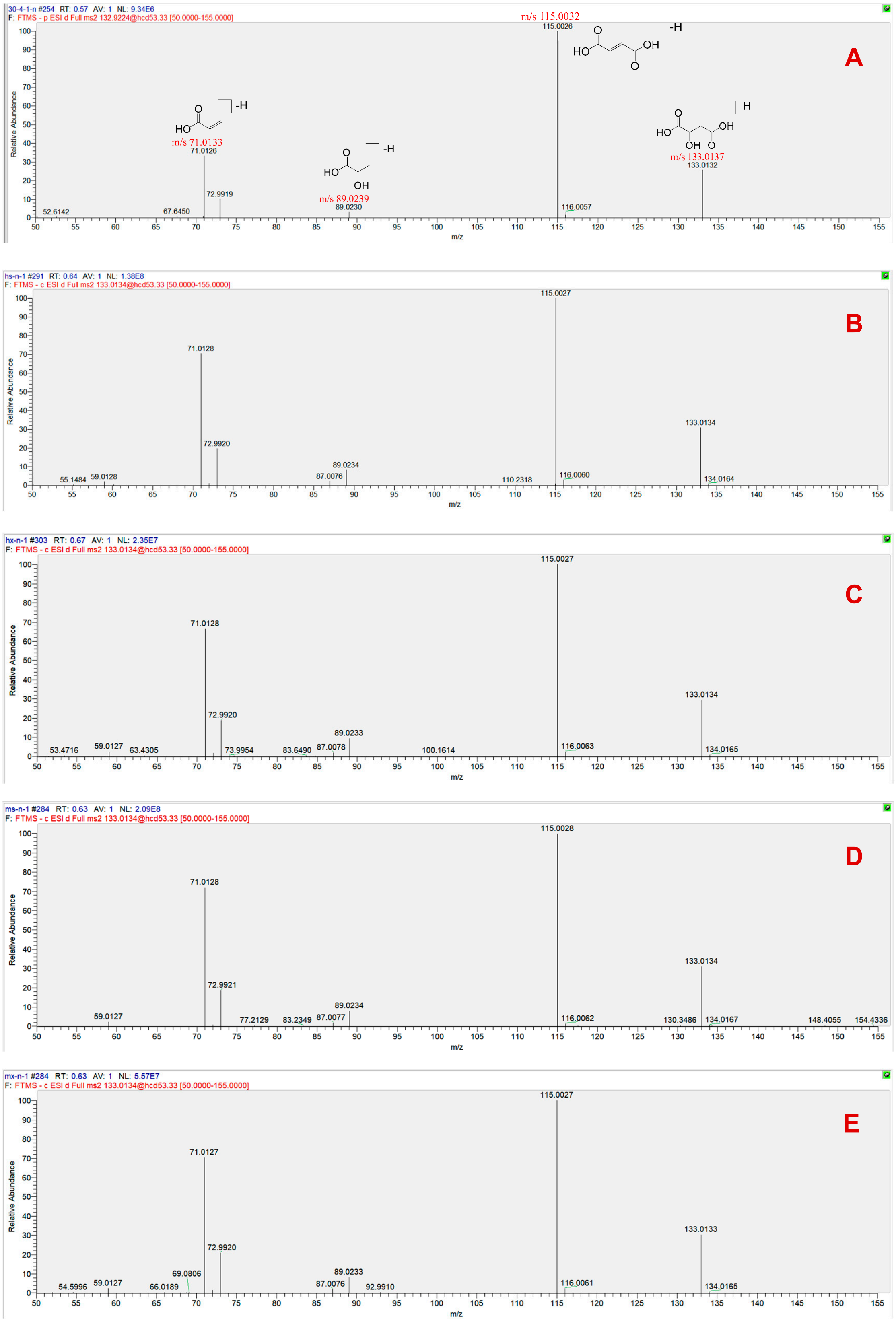


Fig. S1.8.1 The main results of malic acid (CAS 6915-15-7, $C_4H_6O_5$) and its corresponding peak in the TIC diagram using UHPLC-Q-Orbitrap-MS/MS analysis. (**A**) The MS/MS fragments of authentic standard malic acid. (**B**) The MS/MS spectra from chromatographic peak in the CoCA extract. (**C**) The

MS/MS spectra from chromatographic peak in the CoCU extract. (**D**) The MS/MS spectra from chromatographic peak in the CoNA extract. (**E**) The MS/MS spectra from chromatographic peak in the CoNU extract.

**Note**: The m/z values in red are the calculated ones. The m/z calculation was based on the relative atomic masses of C (12.0000), H (1.007825), O (15.994915)[1]

**Identification**: As seen in Fig. S1.8.1, the extract ion peak, MS/MS spectra, and characteristic pears were highly similar. Thus, the chromatographic peaks in the extracts (CoCA, CoCU, CoNA, CoNU) were identified as malic acid (CAS 6915-15-7).

*Suppl. 1.9* Identification of citric acid (CAS 77-92-9, $C_6H_8O_7$, M.W. 192.12 ).

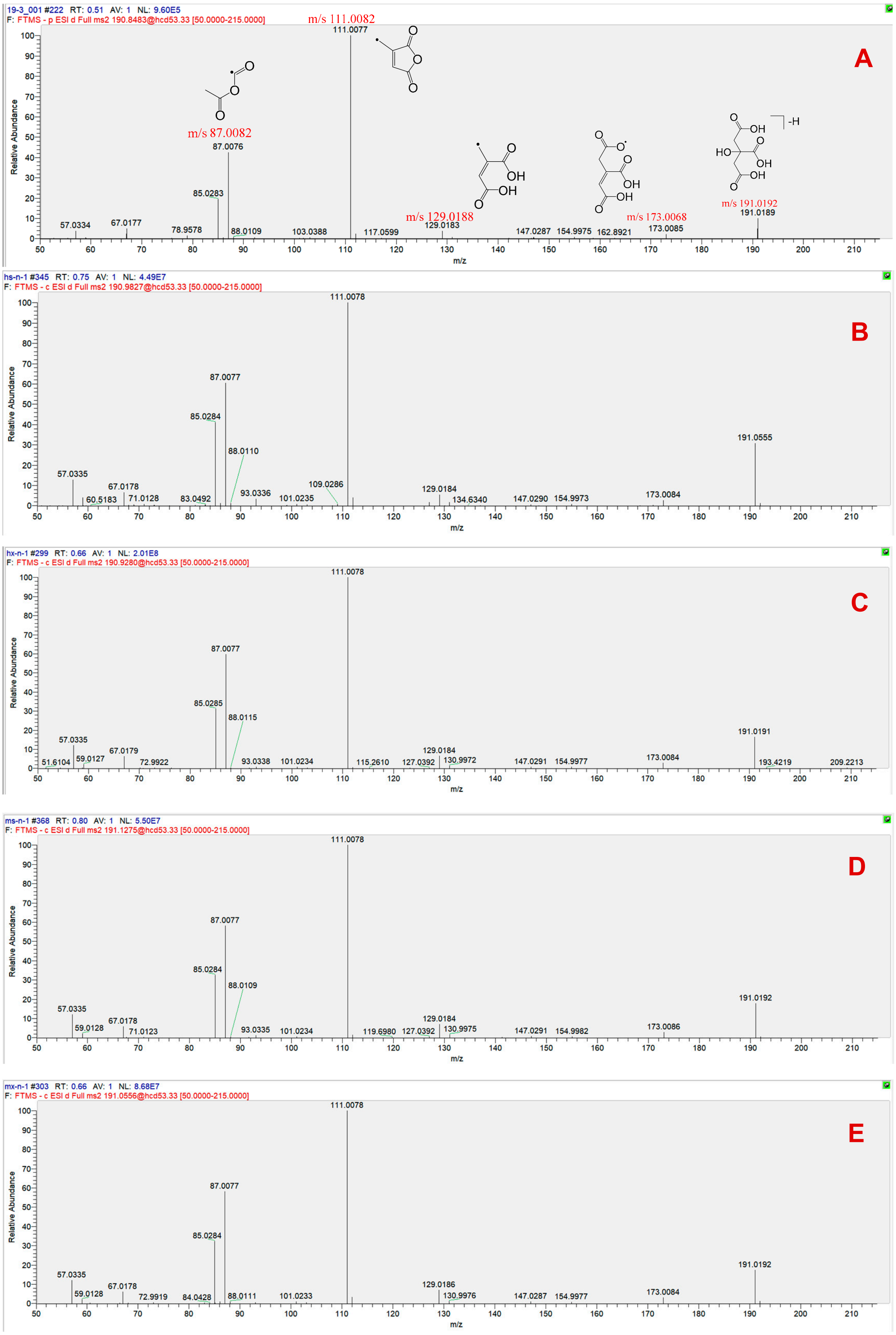


Fig. S1.9.1 The main results of citric acid (CAS 77-92-9, $C_6H_8O_7$) and its corresponding peak in the TIC diagram using UHPLC-Q-Orbitrap-MS/MS analysis. (**A**) The MS/MS fragments of authentic standard citric acid. (**B**) The MS/MS spectra from chromatographic peak in the CoCA extract. (**C**) The

MS/MS spectra from chromatographic peak in the CoCU extract. (**D**) The MS/MS spectra from chromatographic peak in the CoNA extract. (**E**) The MS/MS spectra from chromatographic peak in the CoNU extract.

**Note**: The m/z values in red are the calculated ones. The m/z calculation was based on the relative atomic masses of C (12.0000), H (1.007825), O (15.994915)[1]

**Identification**: As seen in Fig. S1.9.1, the extract ion peak, MS/MS spectra, and characteristic pears were highly similar. Thus, the chromatographic peaks in the extracts (CoCA, CoCU, CoNA, CoNU) were identified as citric acid (CAS 77-92-9).

*Suppl. 1.10* Identification of gallic acid (CAS 149-91-7, $C_7H_6O_5$, M.W. 170.12 ).

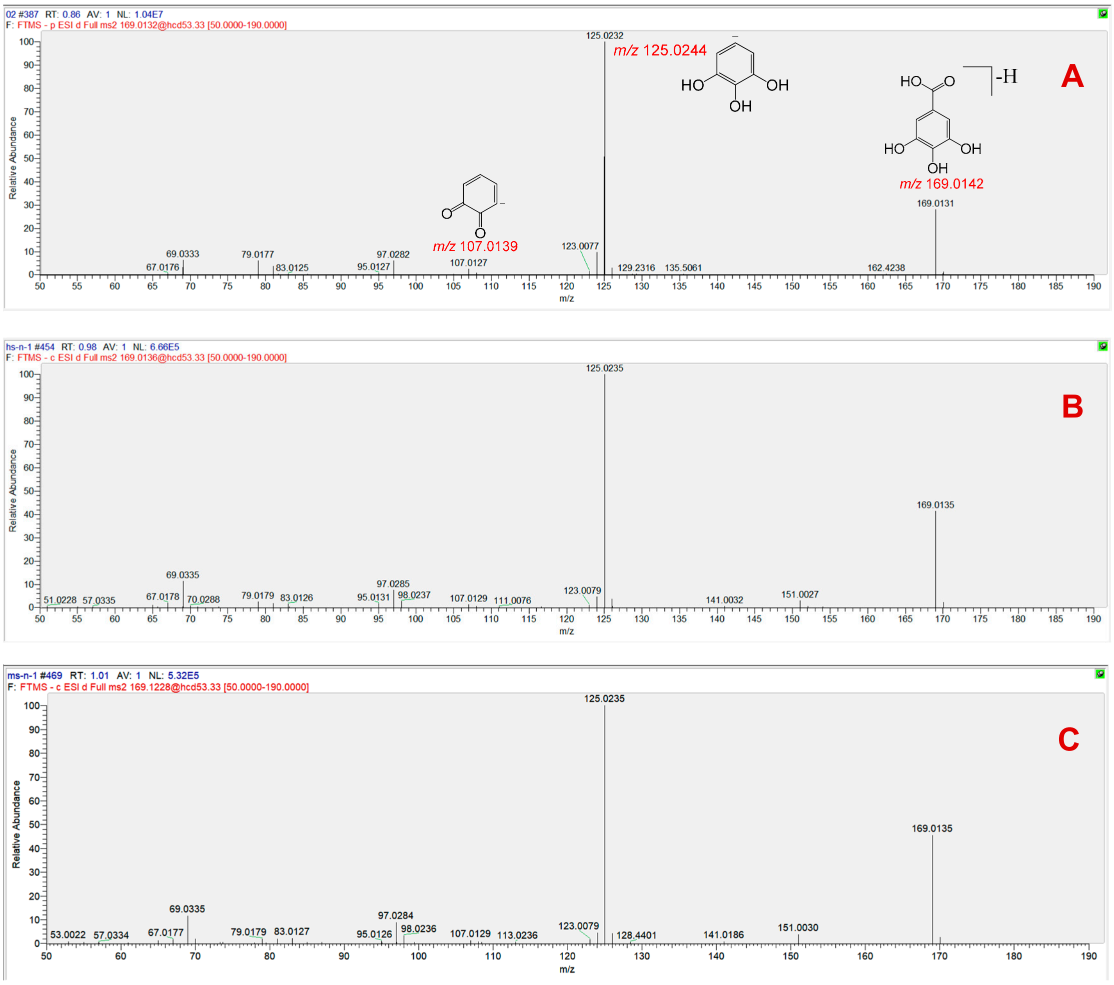


Fig. S1.10.1 The main results of gallic acid (CAS 149-91-7, $C_7H_6O_5$) and its corresponding peak in the TIC diagram using UHPLC-Q-Orbitrap-MS/MS analysis. (**A**) The MS/MS fragments of authentic standard gallic acid. (**B**) The MS/MS spectra from chromatographic peak in the CoCA extract. (**C**) The MS/MS spectra from chromatographic peak in the CoNA extract.

**Note**: The m/z values in red are the calculated ones. The m/z calculation was based on the relative atomic masses of C (12.0000), H (1.007825), O (15.994915)[1]

**Identification**: As seen in Fig. S1.10.1, the extract ion peak, MS/MS spectra, and characteristic pears were highly similar. Thus, the chromatographic peaks in the extracts (CoCA, CoNA) were identified as gallic acid (CAS 149-91-7).

*Suppl. 1.11* Identification of *L*-leucine (CAS 61-90-5, $C_6H_{13}NO_2$, M.W. 131.17 ).

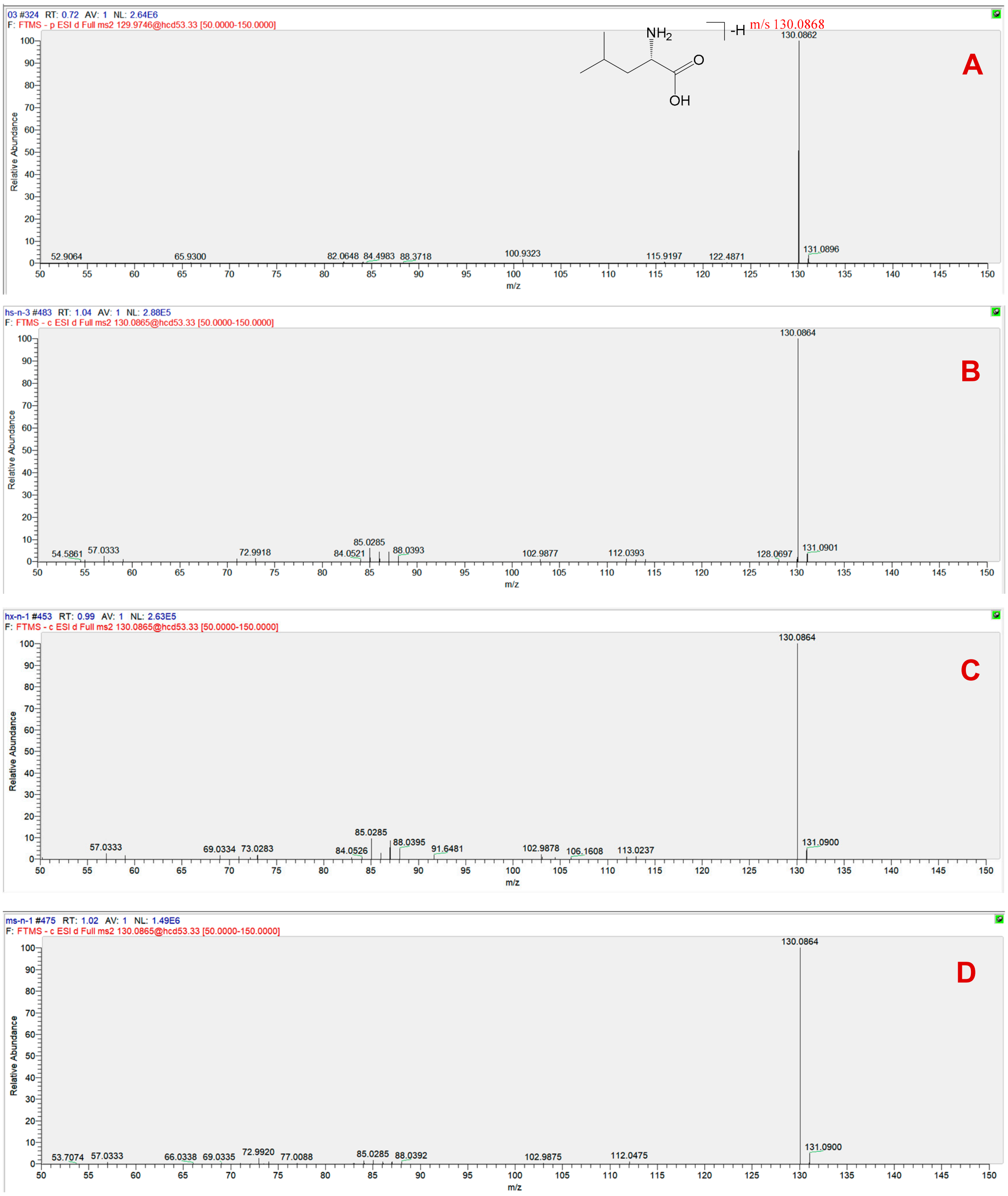


Fig. S1.11.1 The main results of *L*-leucine (CAS 61-90-5, $C_6H_{13}NO_2$) and its corresponding peak in the TIC diagram using UHPLC-Q-Orbitrap-MS/MS analysis. (**A**) The MS/MS fragments of authentic standard *L*-leucine. (**B**) The MS/MS spectra from chromatographic peak in the CoCA extract. (**C**) The MS/MS spectra from chromatographic peak in the CoCU extract. (**D**) The MS/MS spectra from chromatographic peak in the CoNA extract.

**Note**: The m/z values in red are the calculated ones. The m/z calculation was based on the relative atomic masses of C (12.0000), H (1.007825), O (15.994915), and N (14.003074)[1]

**Identification**: As seen in Fig. S1.11.1, the extract ion peak, MS/MS spectra, and characteristic pears were highly similar. Thus, the chromatographic peaks in the extracts (CoCA, CoCU, CoNA) were identified as *L*-leucine (CAS 61-90-5).

*Suppl. 1.12* Identification of *L*-phenylalanine (CAS 63-91-2, $C_9H_{11}NO_2$, M.W. 165.19 ).

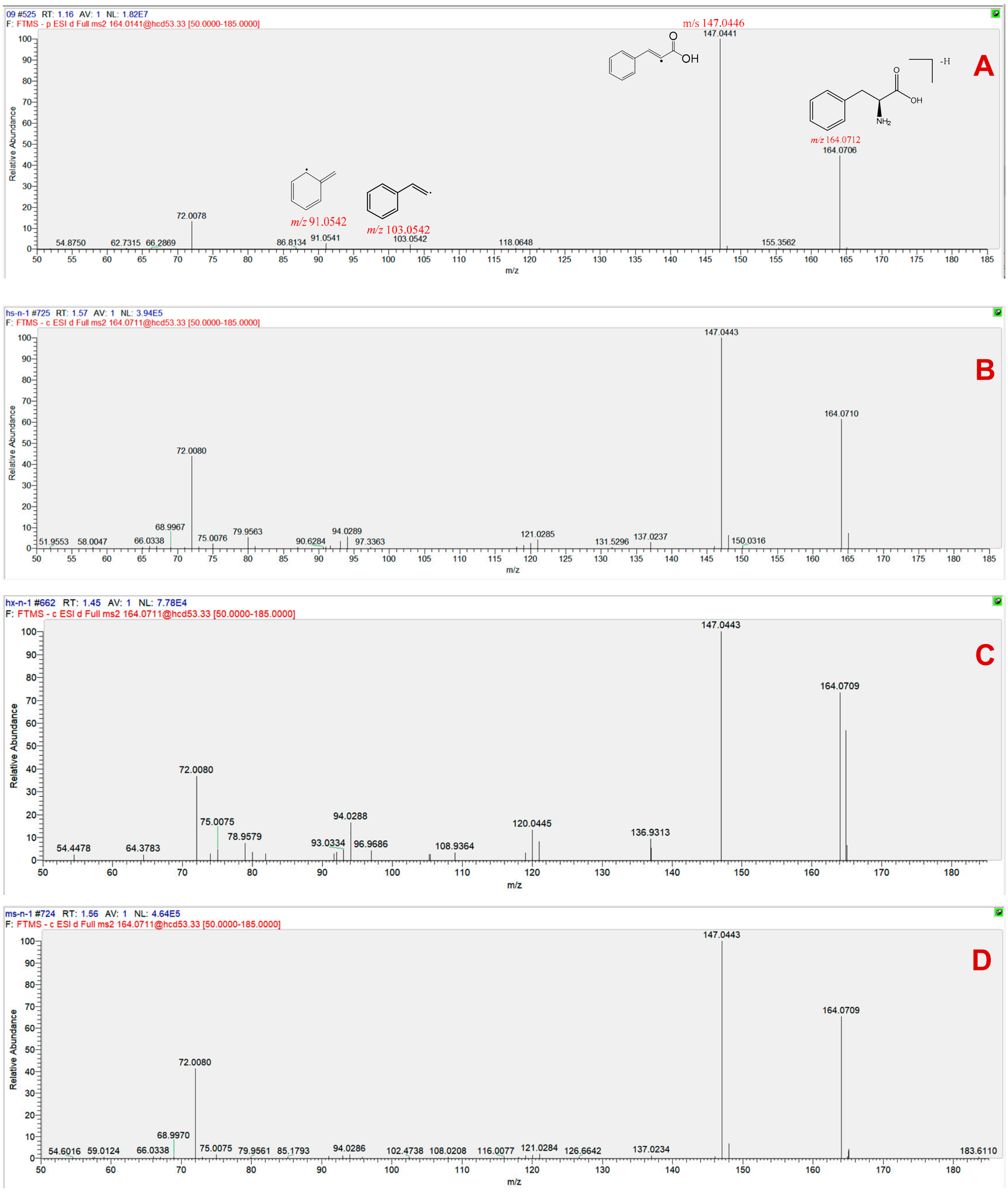


Fig. S1.12.1 The main results of *L*-phenylalanine (CAS 63-91-2, $C_9H_{11}NO_2$) and its corresponding peak in the TIC diagram using UHPLC-Q-Orbitrap-MS/MS analysis. (**A**) The MS/MS fragments of authentic standard *L*-phenylalanine. (**B**) The MS/MS spectra from chromatographic peak in the CoCA extract. (**C**) The MS/MS spectra from chromatographic peak in the CoCU extract. (**D**) The MS/MS spectra from chromatographic peak in the CoNA extract.

**Note**: The m/z values in red are the calculated ones. The m/z calculation was based on the relative atomic masses of C (12.0000), H (1.007825), O (15.994915), and N (14.003074)[1]

**Identification**: As seen in Fig. S1.12.1, the extract ion peak, MS/MS spectra, and characteristic pears were highly similar. Thus, the chromatographic peaks in the extracts

(CoCA, CoCU, CoNA) were identified as *L*-phenylalanine (CAS 63-91-2).

*Suppl. 1.13* Identification of 5-caffeoylquinic acid (CAS 906-33-2, $C_{16}H_{18}O_9$, M.W. 354.31).

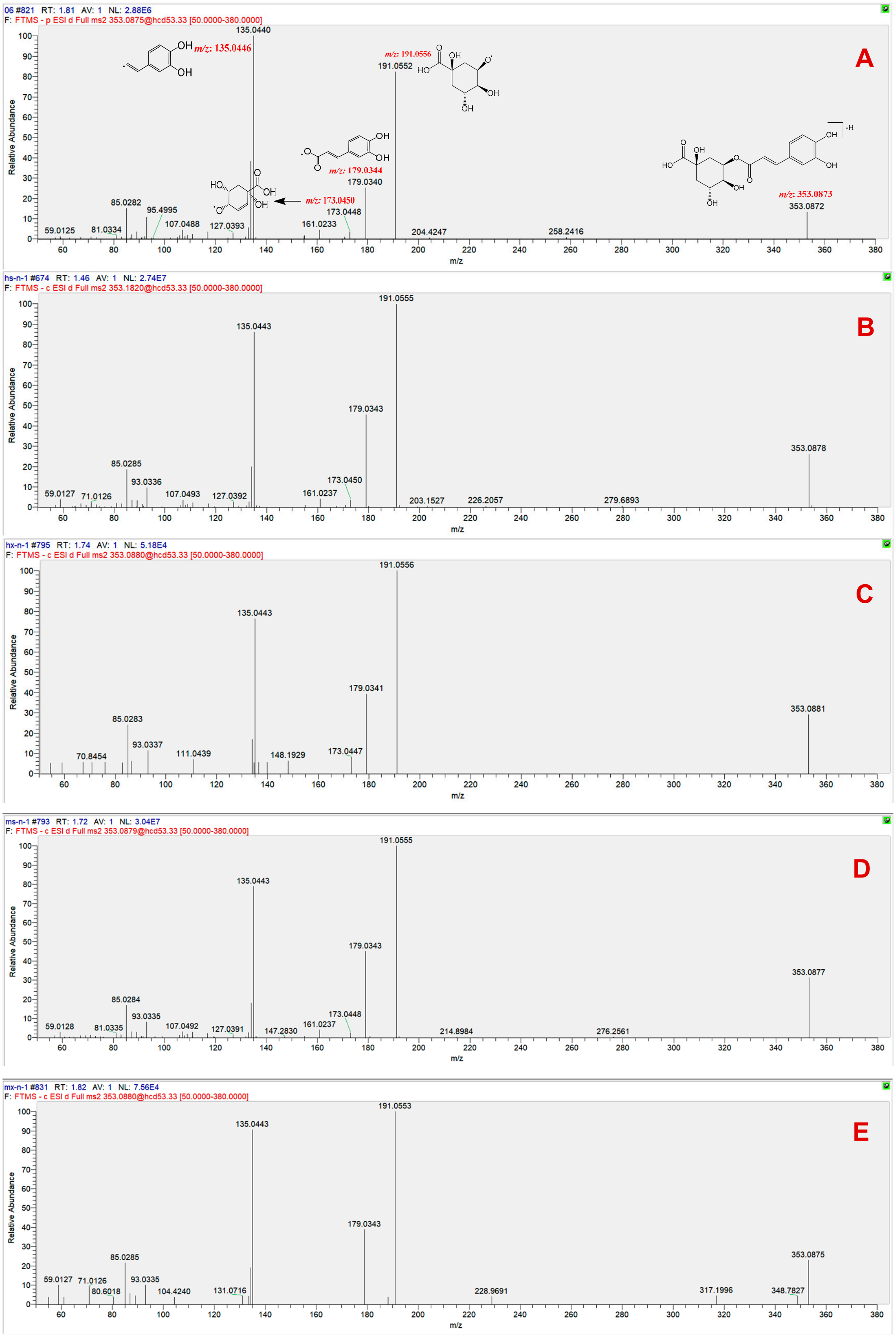


Fig. S1.13.1 The main results of 5-caffeoylquinic acid (CAS 906-33-2, $C_{16}H_{18}O_9$) and its corresponding peak in the TIC diagram using UHPLC-Q-Orbitrap-MS/MS analysis. (**A**) The MS/MS fragments of

authentic standard 5-caffeoylquinic acid. (**B**) The MS/MS spectra from chromatographic peak in the CoCA extract. (**C**) The MS/MS spectra from chromatographic peak in the CoCU extract. (**D**) The MS/MS spectra from chromatographic peak in the CoNA extract. (**E**) The MS/MS spectra from chromatographic peak in the CoNU extract.

**Note**: The m/z values in red are the calculated ones. The m/z calculation was based on the relative atomic masses of C (12.0000), H (1.007825), O (15.994915)[1]

**Identification**: As seen in Fig. S1.13.1, the extract ion peak, MS/MS spectra, and characteristic pears were highly similar. Thus, the chromatographic peaks in the extracts (CoCA, CoCU, CoNA, CoNU) were identified as 5-caffeoylquinic acid (CAS 906-33-2).

*Suppl. 1.14* Identification of 1,2-benzenediol (CAS 120-80-9, $C_6H_6O_2$, M.W. 110.11).

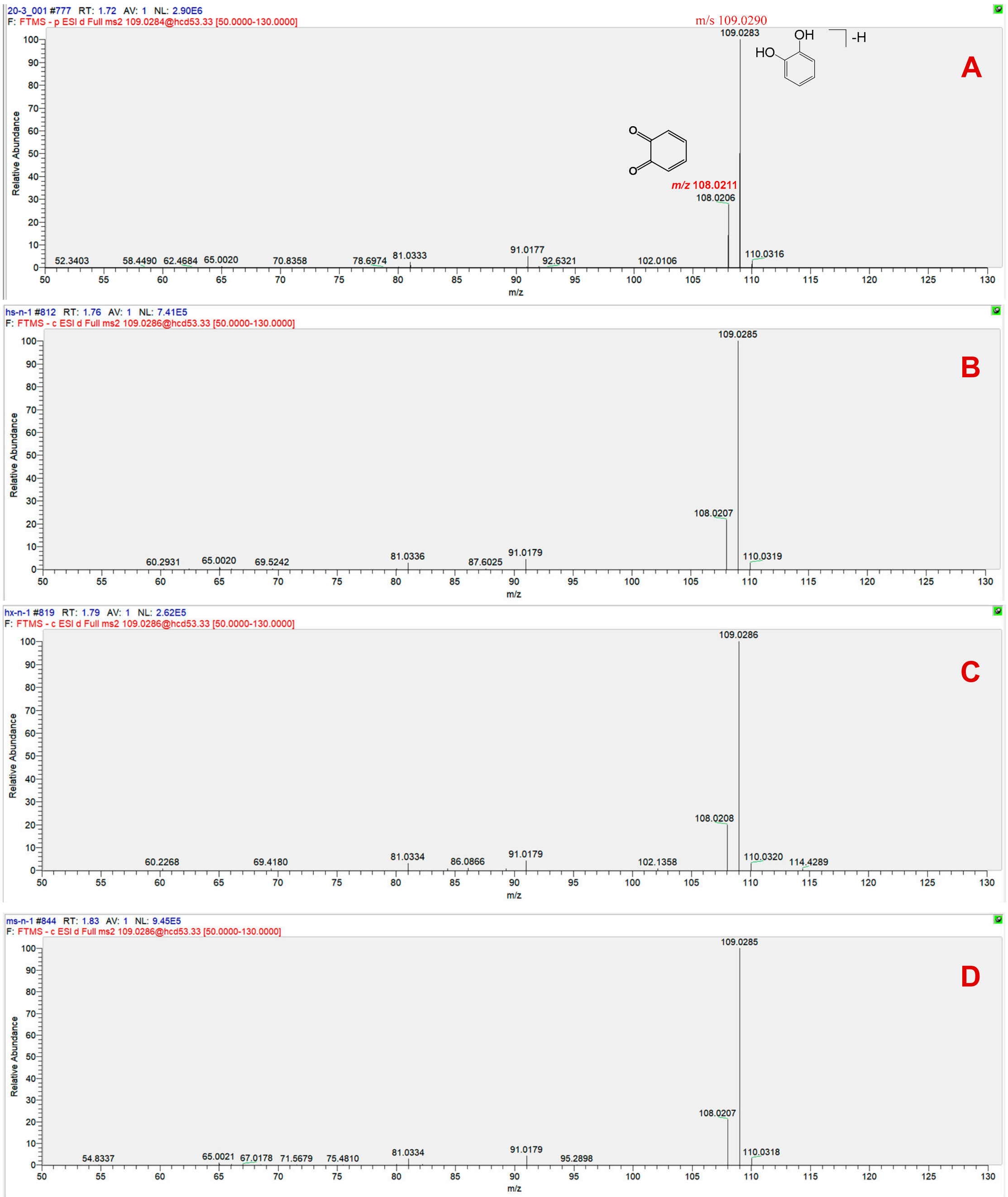


Fig. S1.14.1 The main results of 1,2-benzenediol (CAS 120-80-9, $C_6H_6O_2$) and its corresponding peak in the TIC diagram using UHPLC-Q-Orbitrap-MS/MS analysis. (**A**) The MS/MS fragments of authentic standard 1,2-benzenediol. (**B**) The MS/MS spectra from chromatographic peak in the CoCA extract. (**C**) The MS/MS spectra from chromatographic peak in the CoCU extract. (**D**) The MS/MS spectra from chromatographic peak in the CoNA extract.

**Note**: The m/z values in red are the calculated ones. The m/z calculation was based on the relative atomic masses of C (12.0000), H (1.007825), O (15.994915)[1]

**Identification**: As seen in Fig. S1.14.1, the extract ion peak, MS/MS spectra, and characteristic pears were highly similar. Thus, the chromatographic peaks in the extracts (CoCA, CoCU, CoNA) were identified as 1,2-benzenediol (CAS 120-80-9).

*Suppl. 1.15* Identification of protocatechuic acid (CAS 99-50-3, $C_7H_6O_4$, M.W. 154.12).

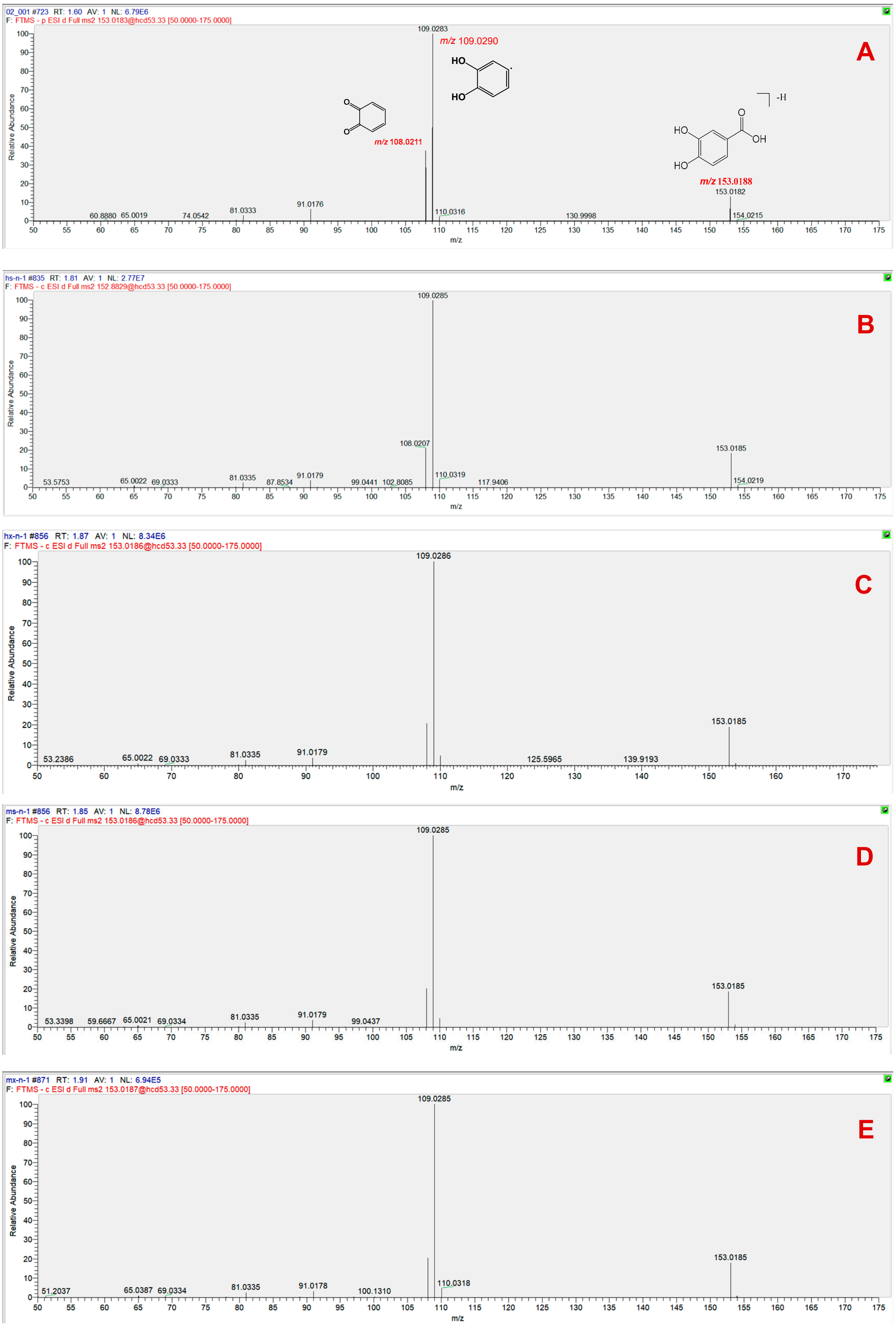


Fig. S1.15.1 The main results of protocatechuic acid (CAS 99-50-3, $C_7H_6O_4$) and its corresponding peak in the TIC diagram using UHPLC-Q-Orbitrap-MS/MS analysis. (**A**) The MS/MS fragments of authentic standard protocatechuic acid. (**B**) The MS/MS spectra from chromatographic peak in the

CoCA extract. (**C**) The MS/MS spectra from chromatographic peak in the CoCU extract. (**D**) The MS/MS spectra from chromatographic peak in the CoNA extract. (**E**) The MS/MS spectra from chromatographic peak in the CoNU extract.

**Note**: The m/z values in red are the calculated ones. The m/z calculation was based on the relative atomic masses of C (12.0000), H (1.007825), O (15.994915)[1]

**Identification**: As seen in Fig. S1.15.1, the extract ion peak, MS/MS spectra, and characteristic pears were highly similar. Thus, the chromatographic peaks in the extracts (CoCA, CoCU, CoNA, CoNU) were identified as protocatechuic acid (CAS 99-50-3).

*Suppl. 1.16* Identification of resorcine (CAS 108-46-3, $C_6H_6O_2$, M.W. 110.11).

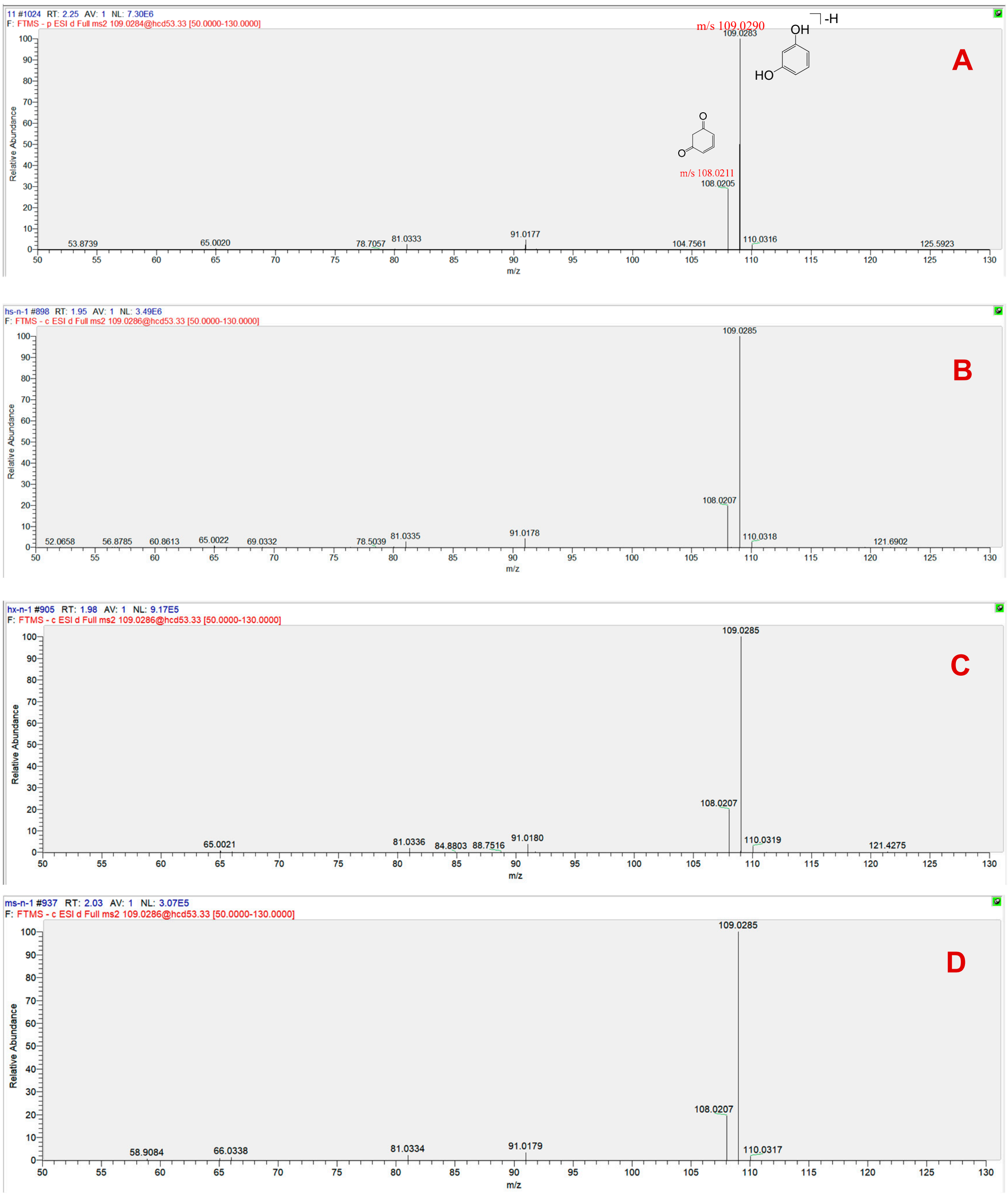


Fig. S1.16.1 The main results of resorcine (CAS 108-46-3, $C_6H_6O_2$) and its corresponding peak in the TIC diagram using UHPLC-Q-Orbitrap-MS/MS analysis. (**A**) The MS/MS fragments of authentic standard resorcine. (**B**) The MS/MS spectra from chromatographic peak in the CoCA extract. (**C**) The MS/MS spectra from chromatographic peak in the CoCU extract. (**D**) The MS/MS spectra from chromatographic peak in the CoNA extract.

**Note**: The m/z values in red are the calculated ones. The m/z calculation was based on the relative atomic masses of C (12.0000), H (1.007825), O (15.994915)[1]

**Identification**: As seen in Fig. S1.16.1, the extract ion peak, MS/MS spectra, and characteristic pears were highly similar. Thus, the chromatographic peaks in the extracts (CoCA, CoCU, CoNA) were identified as resorcine (CAS 108-46-3).

*Suppl. 1.17* Identification of 3,4-dihydroxy-5-methoxybenzoic acid (CAS 3934-84-7, $C_8H_8O_5$, M.W. 184.15).

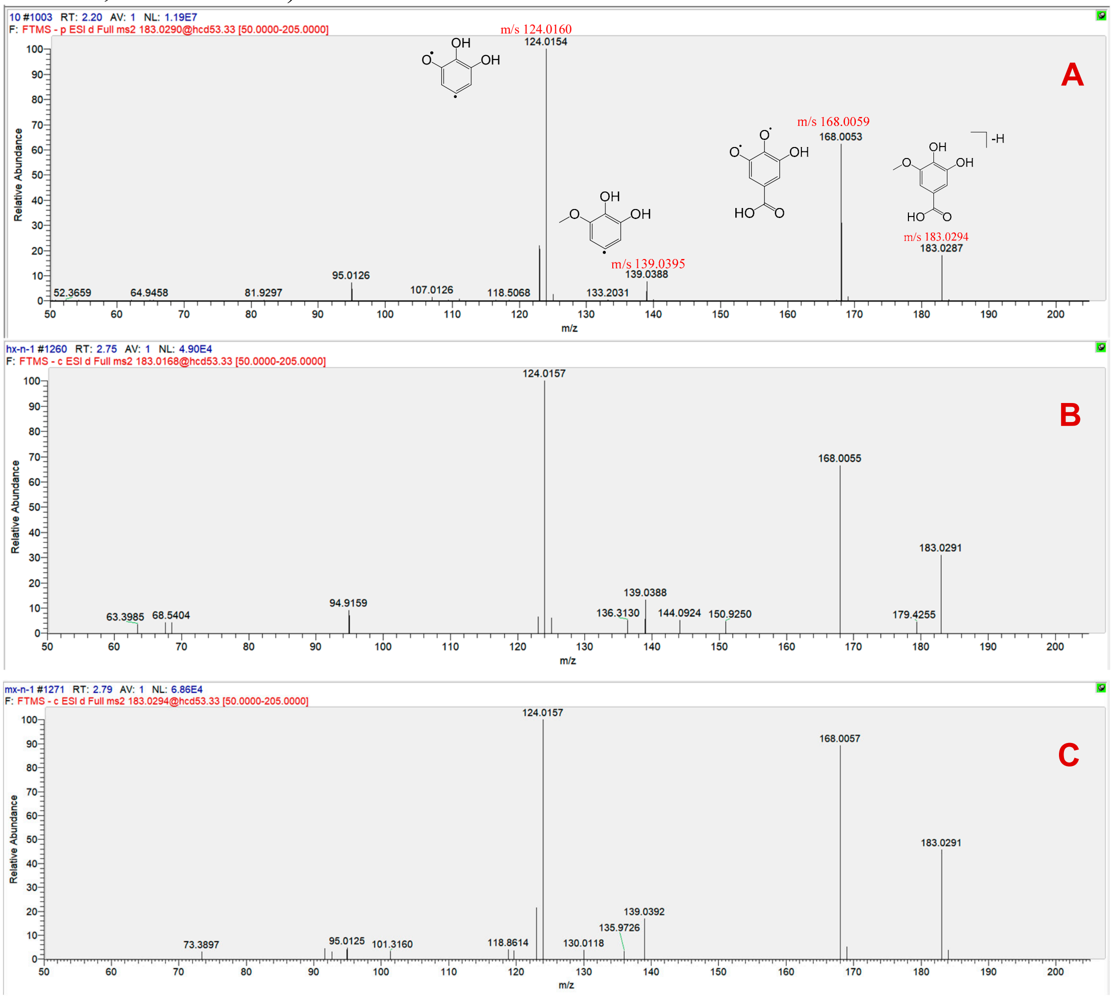


Fig. S1.17.1 The main results of 3,4-dihydroxy-5-methoxybenzoic acid (CAS 3934-84-7, $C_8H_8O_5$) and its corresponding peak in the TIC diagram using UHPLC-Q-Orbitrap-MS/MS analysis. (**A**) The MS/MS fragments of authentic standard 3,4-dihydroxy-5-methoxybenzoic acid. (**B**) The MS/MS spectra from chromatographic peak in the CoCU extract. (**C**) The MS/MS spectra from chromatographic peak in the CoNU extract.

**Note**: The m/z values in red are the calculated ones. The m/z calculation was based on the relative atomic masses of C (12.0000), H (1.007825), O (15.994915)[1]

**Identification**: As seen in Fig. S1.17.1, the extract ion peak, MS/MS spectra, and characteristic pears were highly similar. Thus, the chromatographic peaks in the extracts (CoCU, CoNU) were identified as 3,4-dihydroxy-5-methoxybenzoic acid (CAS 3934-84-7).

*Suppl. 1.18* Identification of 3,4-dihydroxybenzaldehyde (CAS 139-85-5, $C_7H_6O_3$, M.W. 138.12 ).

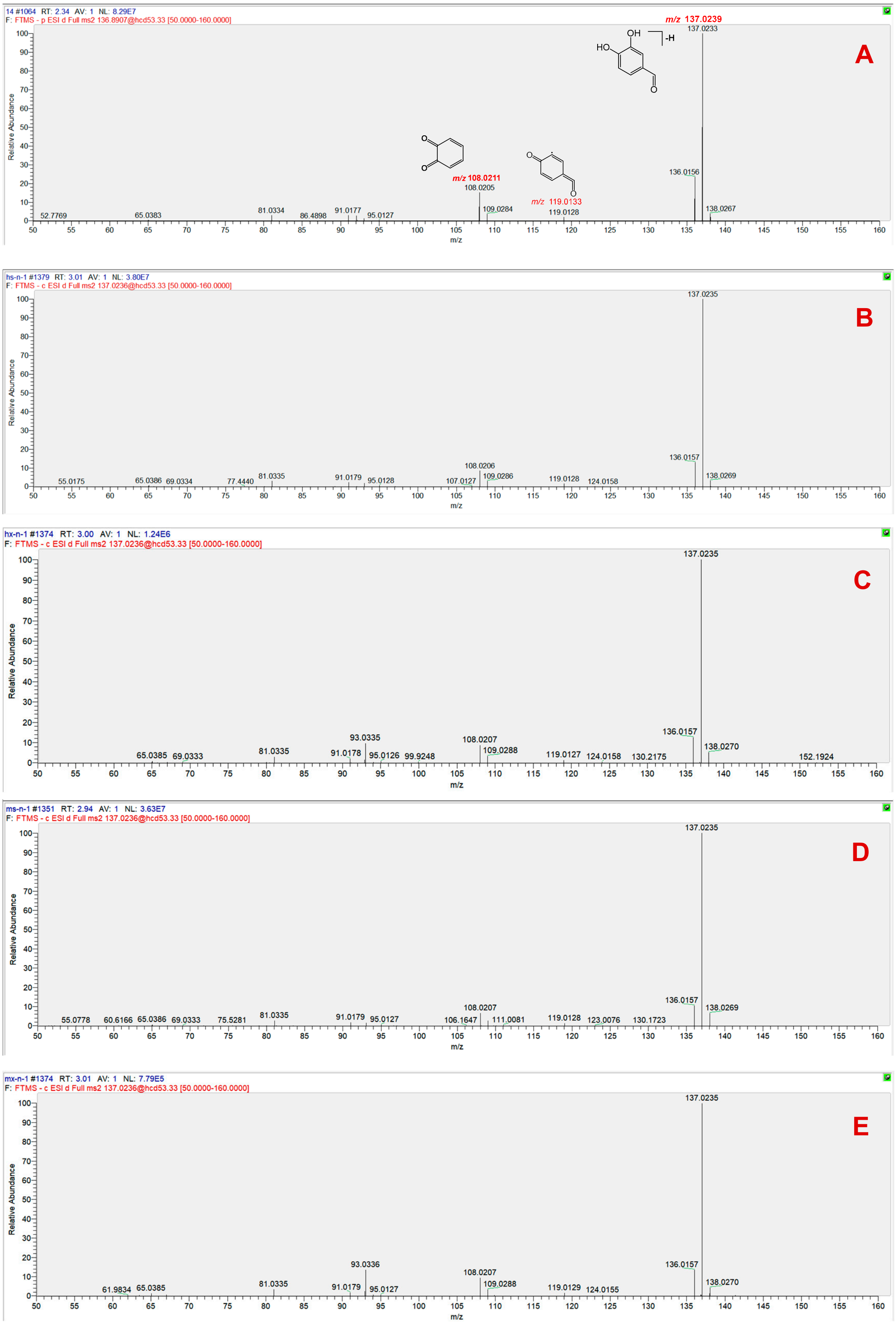


Fig. S1.18.1 The main results of 3,4-dihydroxybenzaldehyde (CAS 139-85-5, $C_7H_6O_3$) and its corresponding peak in the TIC diagram using UHPLC-Q-Orbitrap-MS/MS analysis. (**A**) The MS/MS

fragments of authentic standard 3,4-dihydroxybenzaldehyde. (**B**) The MS/MS spectra from chromatographic peak in the CoCA extract. (**C**) The MS/MS spectra from chromatographic peak in the CoCU extract. (**D**) The MS/MS spectra from chromatographic peak in the CoNA extract. (**E**) The MS/MS spectra from chromatographic peak in the CoNU extract.

**Note**: The m/z values in red are the calculated ones. The m/z calculation was based on the relative atomic masses of C (12.0000), H (1.007825), O (15.994915)[1]

**Identification**: As seen in Fig. S1.18.1, the extract ion peak, MS/MS spectra, and characteristic pears were highly similar. Thus, the chromatographic peaks in the extracts (CoCA, CoCU, CoNA, CoNU) were identified as 3,4-dihydroxybenzaldehyde (CAS 139-85-5).

*Suppl. 1.19* Identification of 4-hydroxybenzoic acid (CAS 99-96-7, $C_7H_6O_3$, M.W. 138.12).

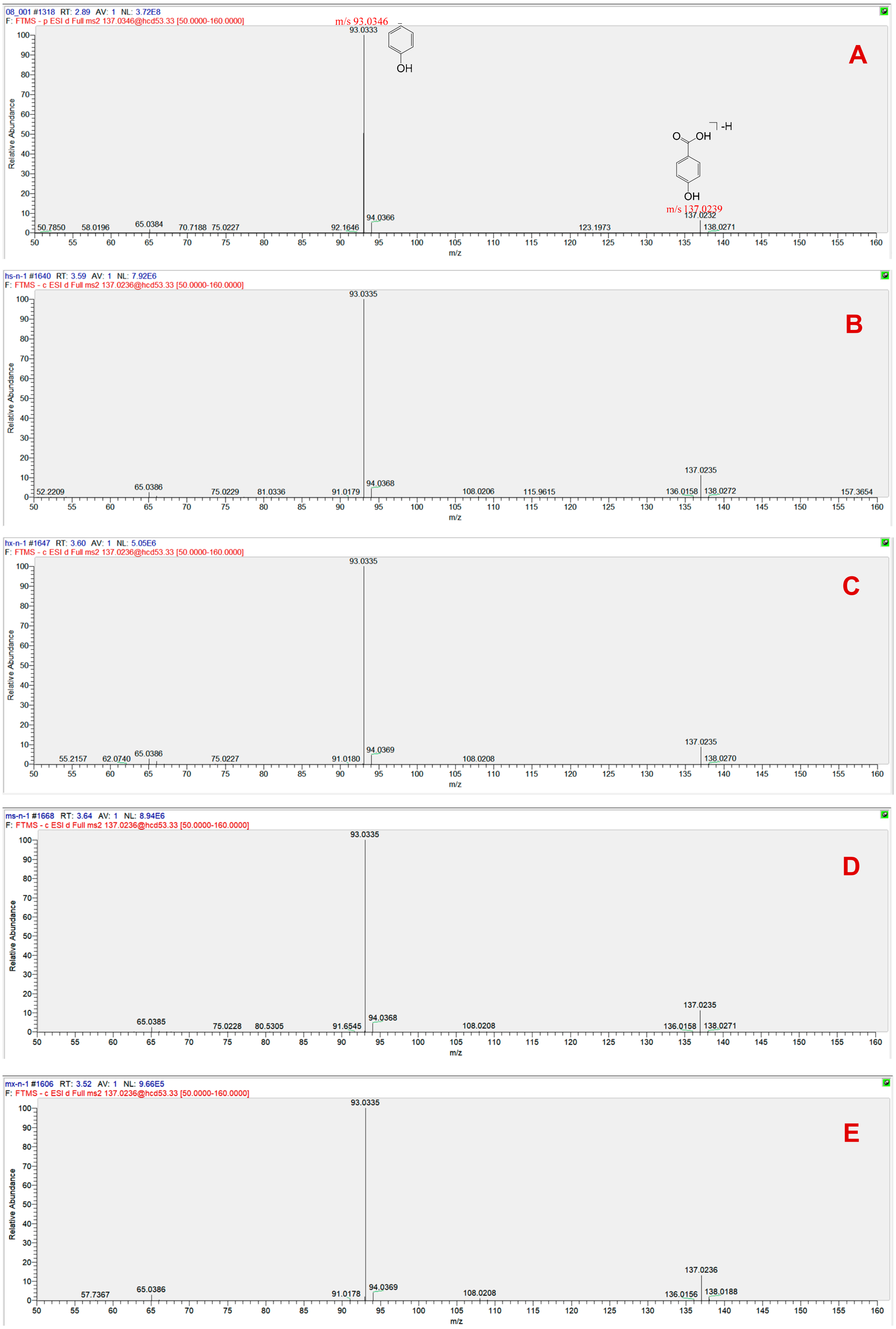


Fig. S1.19.1 The main results of 4-hydroxybenzoic acid (CAS 99-96-7, $C_7H_6O_3$) and its corresponding peak in the TIC diagram using UHPLC-Q-Orbitrap-MS/MS analysis. (**A**) The MS/MS fragments of

authentic standard 4-hydroxybenzoic acid. (**B**) The MS/MS spectra from chromatographic peak in the CoCA extract. (**C**) The MS/MS spectra from chromatographic peak in the CoCU extract. (**D**) The MS/MS spectra from chromatographic peak in the CoNA extract. (**E**) The MS/MS spectra from chromatographic peak in the CoNU extract.

**Note**: The m/z values in red are the calculated ones. The m/z calculation was based on the relative atomic masses of C (12.0000), H (1.007825), O (15.994915)[1]

**Identification**: As seen in Fig. S1.19.1, the extract ion peak, MS/MS spectra, and characteristic pears were highly similar. Thus, the chromatographic peaks in the extracts (CoCA, CoCU, CoNA, CoNU) were identified as 4-hydroxybenzoic acid (CAS 99-96-7).

*Suppl. 1.20* Identification of chlorogenic acid (CAS 327-97-9, $C_{16}H_{18}O_9$, M.W. 354.31 ).

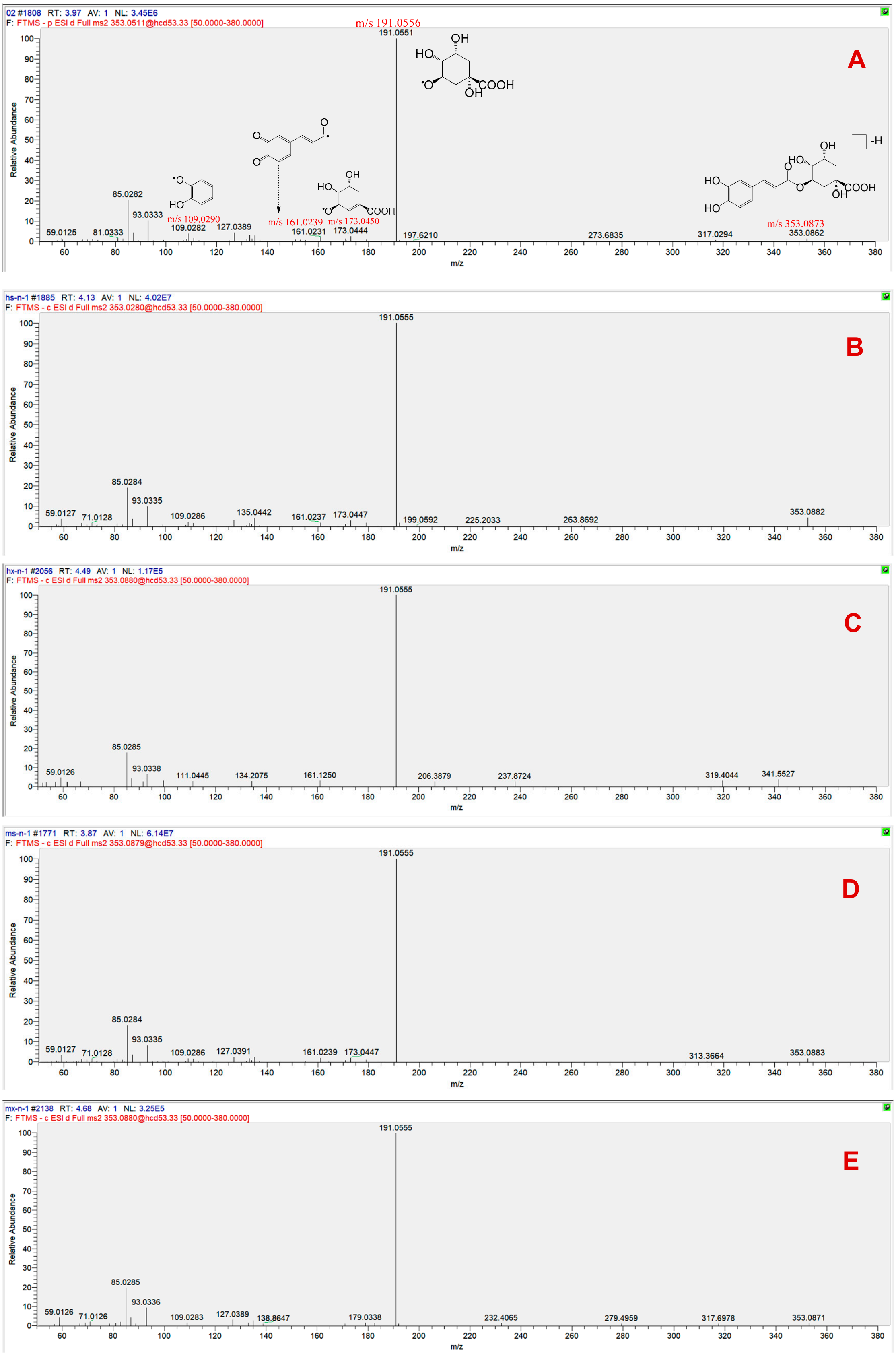


Fig. S1.20.1 The main results of chlorogenic acid (CAS 327-97-9, $C_{16}H_{18}O_9$) and its corresponding peak in the TIC diagram using UHPLC-Q-Orbitrap-MS/MS analysis. (**A**) The MS/MS fragments of

authentic standard chlorogenic acid. (**B**) The MS/MS spectra from chromatographic peak in the CoCA extract. (**C**) The MS/MS spectra from chromatographic peak in the CoCU extract. (**D**) The MS/MS spectra from chromatographic peak in the CoNA extract. (**E**) The MS/MS spectra from chromatographic peak in the CoNU extract.

**Note**: The m/z values in red are the calculated ones. The m/z calculation was based on the relative atomic masses of C (12.0000), H (1.007825), O (15.994915)[1]

**Identification**: As seen in Fig. S1.20.1, the extract ion peak, MS/MS spectra, and characteristic pears were highly similar. Thus, the chromatographic peaks in the extracts (CoCA, CoCU, CoNA, CoNU) were identified as chlorogenic acid (CAS 327-97-9).

*Suppl. 1.21* Identification of 3-hydroxybenzoic acid (CAS 99-06-9, $C_7H_6O_3$, M.W. 138.12 ).

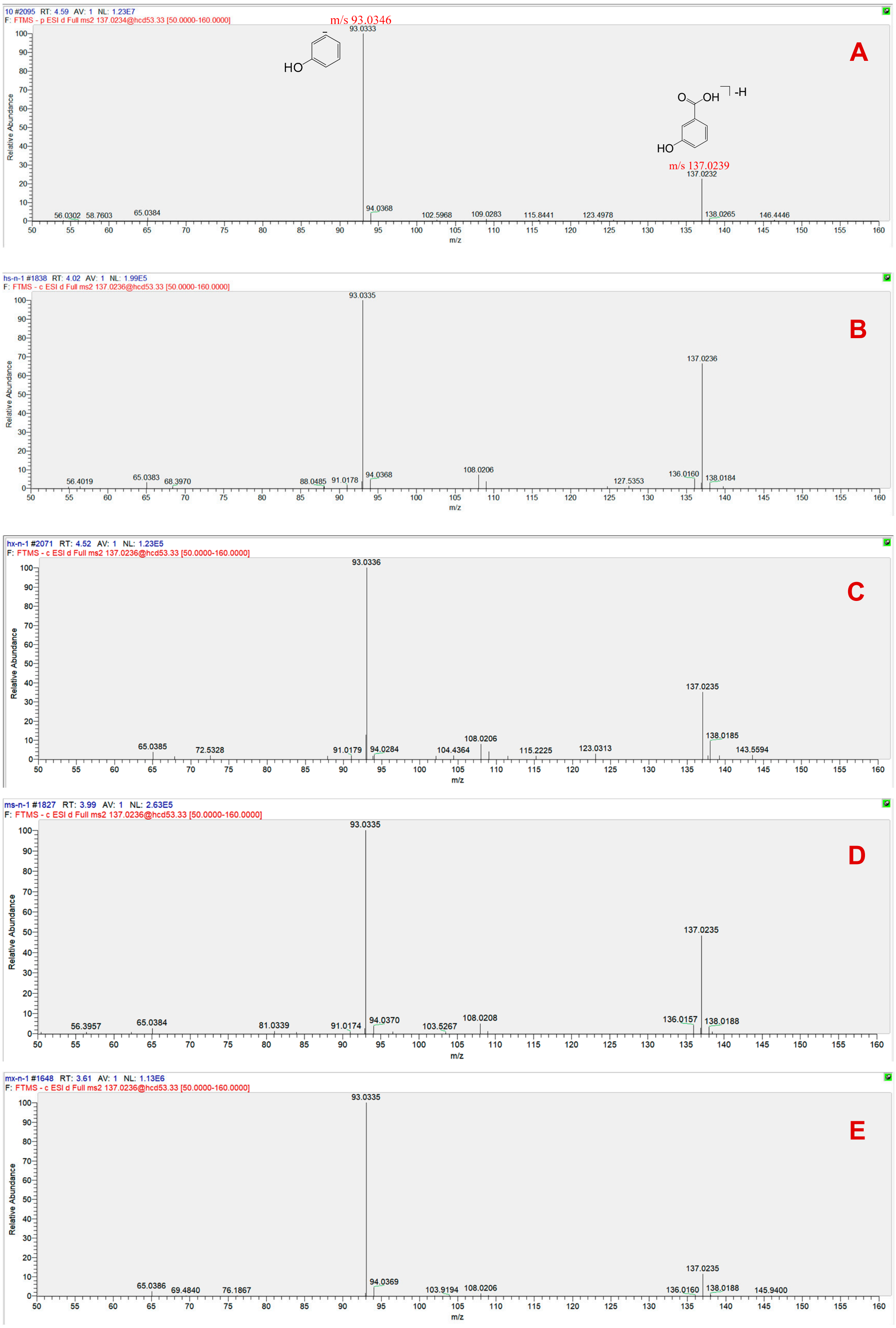


Fig. S1.21.1 The main results of 3-hydroxybenzoic acid (CAS 99-06-9, $C_7H_6O_3$) and its corresponding peak in the TIC diagram using UHPLC-Q-Orbitrap-MS/MS analysis. (**A**) The MS/MS fragments of

authentic standard 3-hydroxybenzoic acid. (**B**) The MS/MS spectra from chromatographic peak in the CoCA extract. (**C**) The MS/MS spectra from chromatographic peak in the CoCU extract. (**D**) The MS/MS spectra from chromatographic peak in the CoNA extract. (**E**) The MS/MS spectra from chromatographic peak in the CoNU extract.

**Note**: The m/z values in red are the calculated ones. The m/z calculation was based on the relative atomic masses of C (12.0000), H (1.007825), O (15.994915)[1]

**Identification**: As seen in Fig. S1.21.1, the extract ion peak, MS/MS spectra, and characteristic pears were highly similar. Thus, the chromatographic peaks in the extracts (CoCA, CoCU, CoNA, CoNU) were identified as 3-hydroxybenzoic acid (CAS 99-06-9).

*Suppl. 1.22* Identification of caffeic acid (CAS 331-39-5, $C_9H_8O_4$, M.W. 180.16).

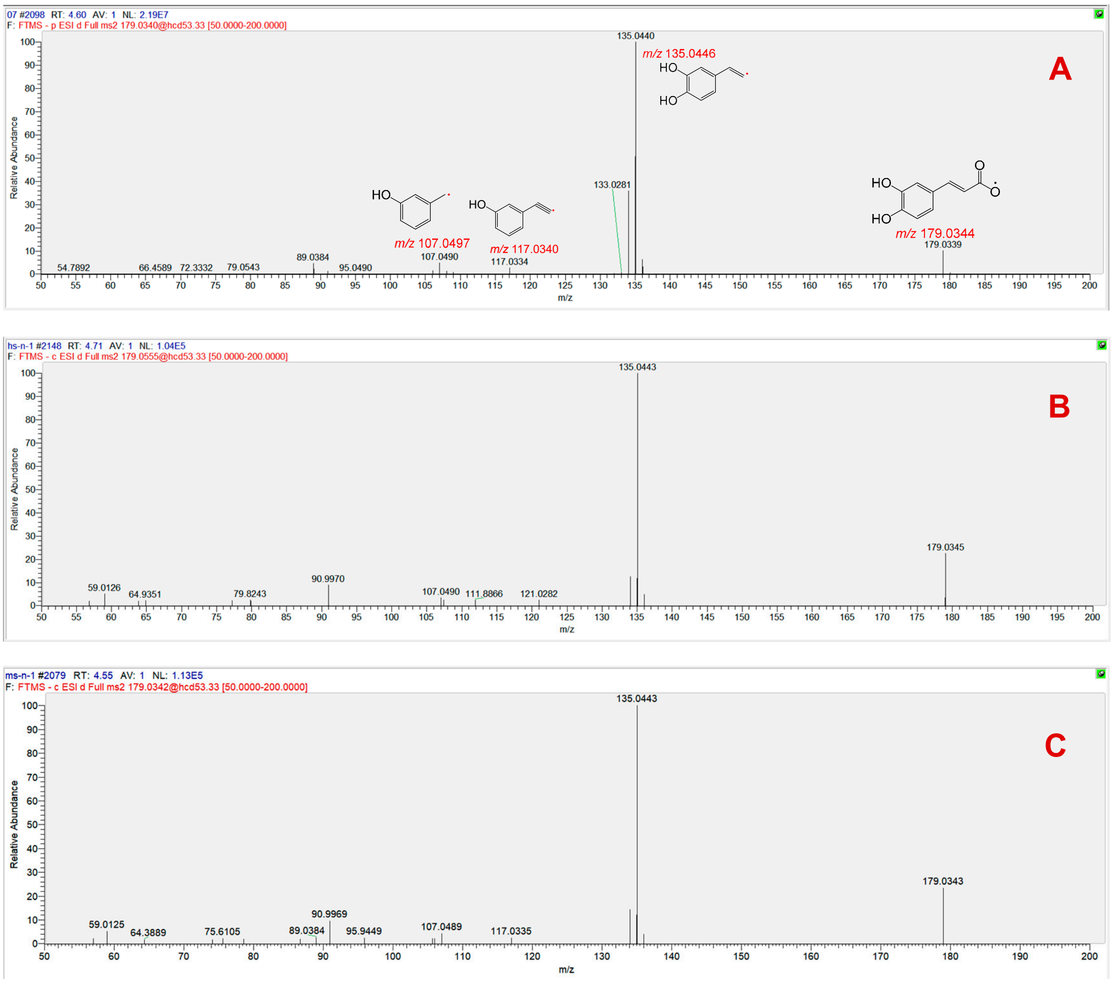


Fig. S1.22.1 The main results of caffeic acid (CAS 331-39-5, $C_9H_8O_4$) and its corresponding peak in the TIC diagram using UHPLC-Q-Orbitrap-MS/MS analysis. (**A**) The MS/MS fragments of authentic standard caffeic acid. (**B**) The MS/MS spectra from chromatographic peak in the CoCA extract. (**C**) The MS/MS spectra from chromatographic peak in the CoNA extract.

**Note**: The m/z values in red are the calculated ones. The m/z calculation was based on the relative atomic masses of C (12.0000), H (1.007825), O (15.994915)[1]

**Identification**: As seen in Fig. S1.22.1, the extract ion peak, MS/MS spectra, and characteristic pears were highly similar. Thus, the chromatographic peaks in the extracts (CoCA, CoNA) were identified as caffeic acid (CAS 331-39-5).

*Suppl. 1.23* Identification of cryptochlorogenic acid (CAS 905-99-7, $C_{16}H_{18}O_9$, M.W. 354.31).

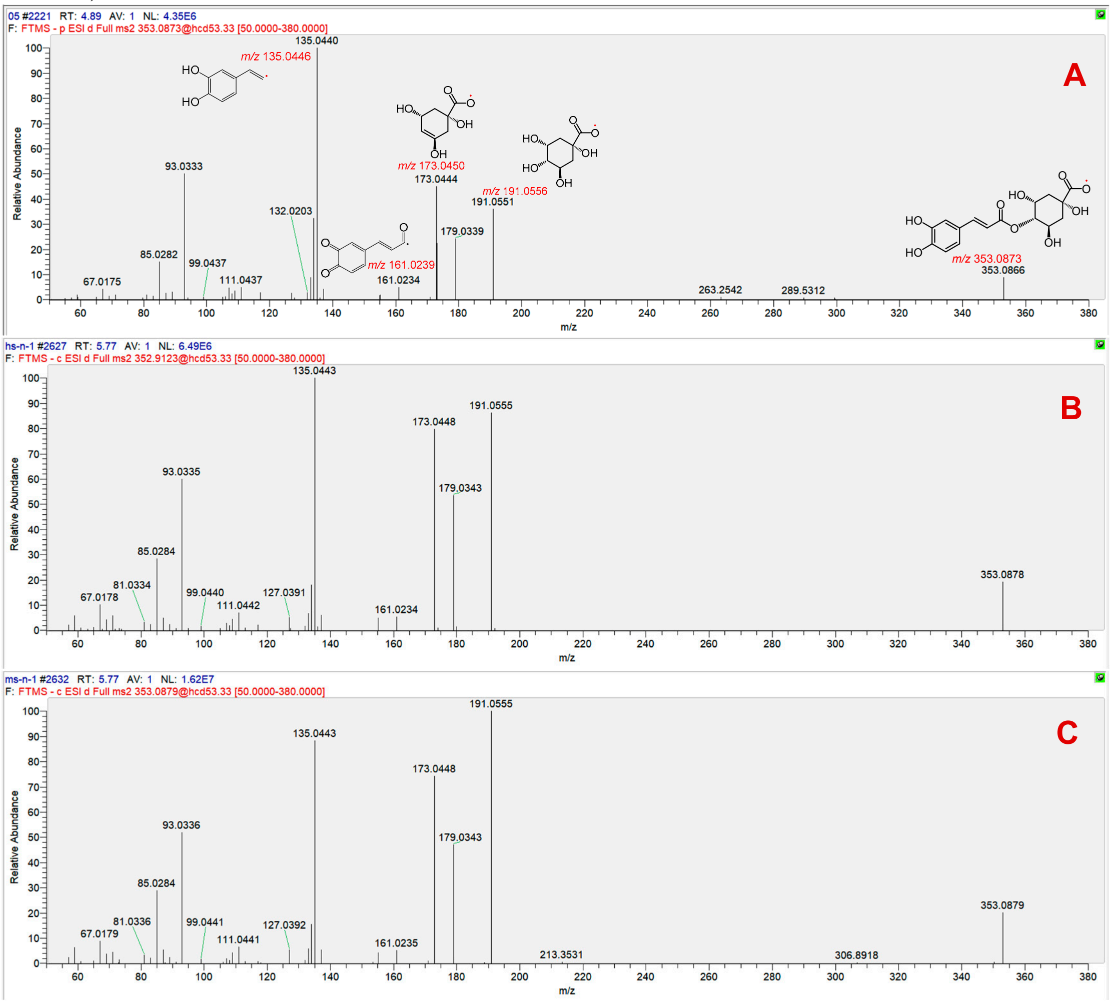

Fig. S1.23.1 The main results of cryptochlorogenic acid (CAS 905-99-7, $C_{16}H_{18}O_9$) and its corresponding peak in the TIC diagram using UHPLC-Q-Orbitrap-MS/MS analysis. (**A**) The MS/MS fragments of authentic standard cryptochlorogenic acid. (**B**) The MS/MS spectra from chromatographic peak in the CoCA extract. (**C**) The MS/MS spectra from chromatographic peak in the CoNA extract.

**Note**: The m/z values in red are the calculated ones. The m/z calculation was based on the relative atomic masses of C (12.0000), H (1.007825), O (15.994915)[1]

**Identification**: As seen in Fig. S1.23.1, the extract ion peak, MS/MS spectra, and characteristic pears were highly similar. Thus, the chromatographic peaks in the extracts (CoCA, CoNA) were identified as cryptochlorogenic acid (CAS 905-99-7).

*Suppl. 1.24* Identification of Cis-4-hydroxycinnamic acid (CAS 4501-31-9, $C_9H_8O_3$, M.W. 164.16).

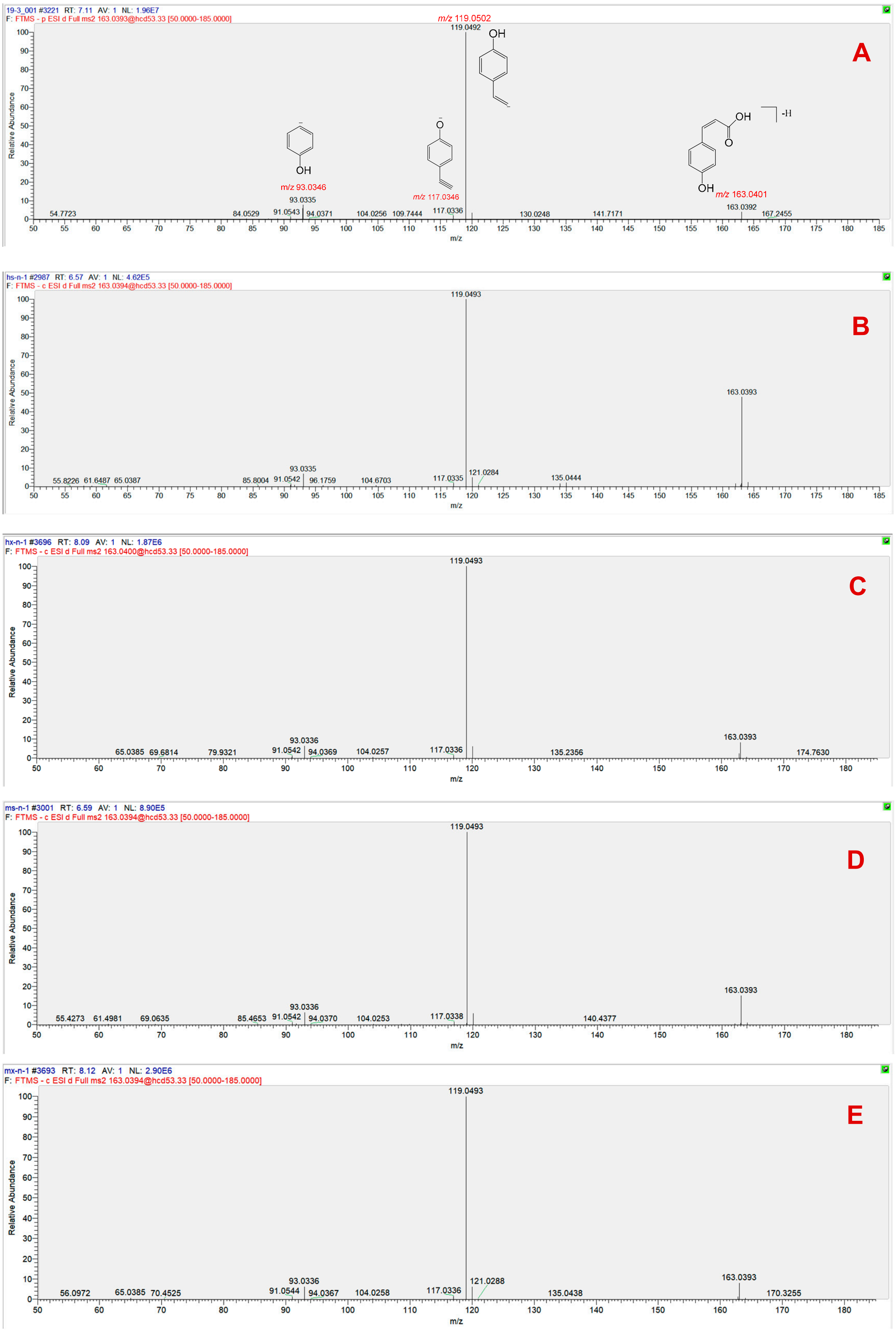


Fig. S1.24.1 The main results of Cis-4-hydroxycinnamic acid (CAS 4501-31-9, $C_9H_8O_3$) and its corresponding peak in the TIC diagram using UHPLC-Q-Orbitrap-MS/MS analysis. (**A**) The MS/MS

fragments of authentic standard Cis-4-hydroxycinnamic acid. (**B**) The MS/MS spectra from chromatographic peak in the CoCA extract. (**C**) The MS/MS spectra from chromatographic peak in the CoCU extract. (**D**) The MS/MS spectra from chromatographic peak in the CoNA extract. (**E**) The MS/MS spectra from chromatographic peak in the CoNU extract.

**Note**: The m/z values in red are the calculated ones. The m/z calculation was based on the relative atomic masses of C (12.0000), H (1.007825), O (15.994915)[1]

**Identification**: As seen in Fig. S1.24.1, the extract ion peak, MS/MS spectra, and characteristic pears were highly similar. Thus, the chromatographic peaks in the extracts (CoCA, CoCU, CoNA, CoNU) were identified as Cis-4-hydroxycinnamic acid (CAS 4501-31-9).

*Suppl. 1.25* Identification of daphnetin (CAS 486-35-1, $C_9H_6O_4$, M.W. 178.14 ).

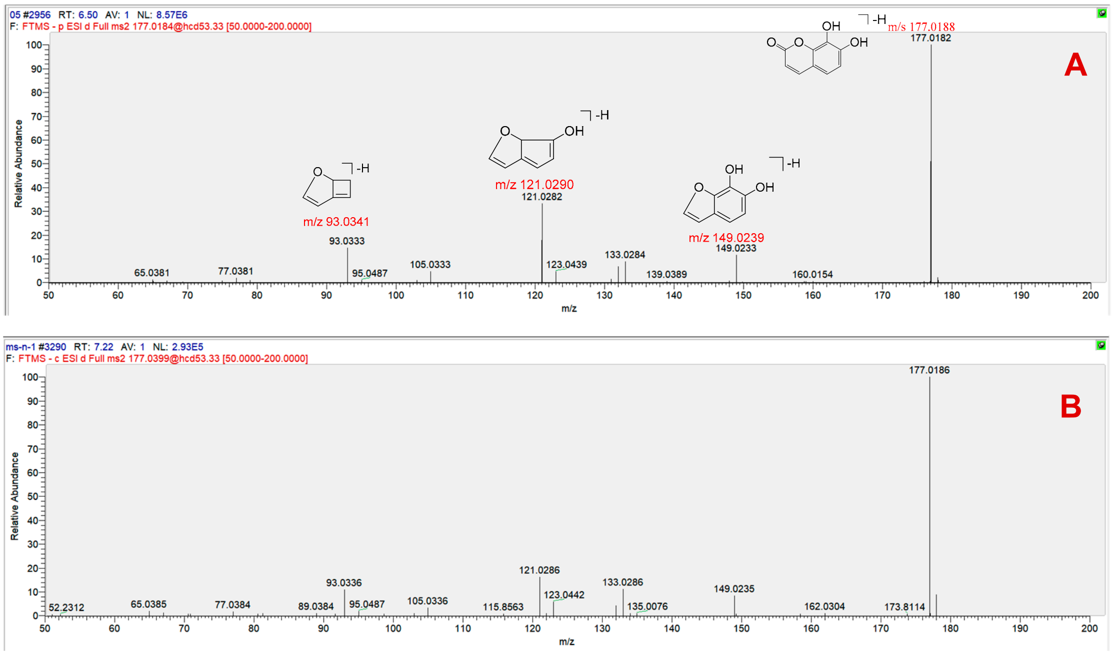


Fig. S1.25.1 The main results of daphnetin (CAS 486-35-1, $C_9H_6O_4$) and its corresponding peak in the TIC diagram using UHPLC-Q-Orbitrap-MS/MS analysis. (**A**) The MS/MS fragments of authentic standard daphnetin. (**B**) The MS/MS spectra from chromatographic peak in the CoNA extract.

**Note**: The m/z values in red are the calculated ones. The m/z calculation was based on the relative atomic masses of C (12.0000), H (1.007825), O (15.994915)[1]

**Identification**: As seen in Fig. S1.25.1, the extract ion peak, MS/MS spectra, and characteristic pears were highly similar. Thus, the chromatographic peaks in the extract (CoNA) were identified as daphnetin (CAS 486-35-1).

*Suppl. 1.26* Identification of syringic acid (CAS 530-57-4, $C_9H_{10}O_5$, M.W. 198.17).

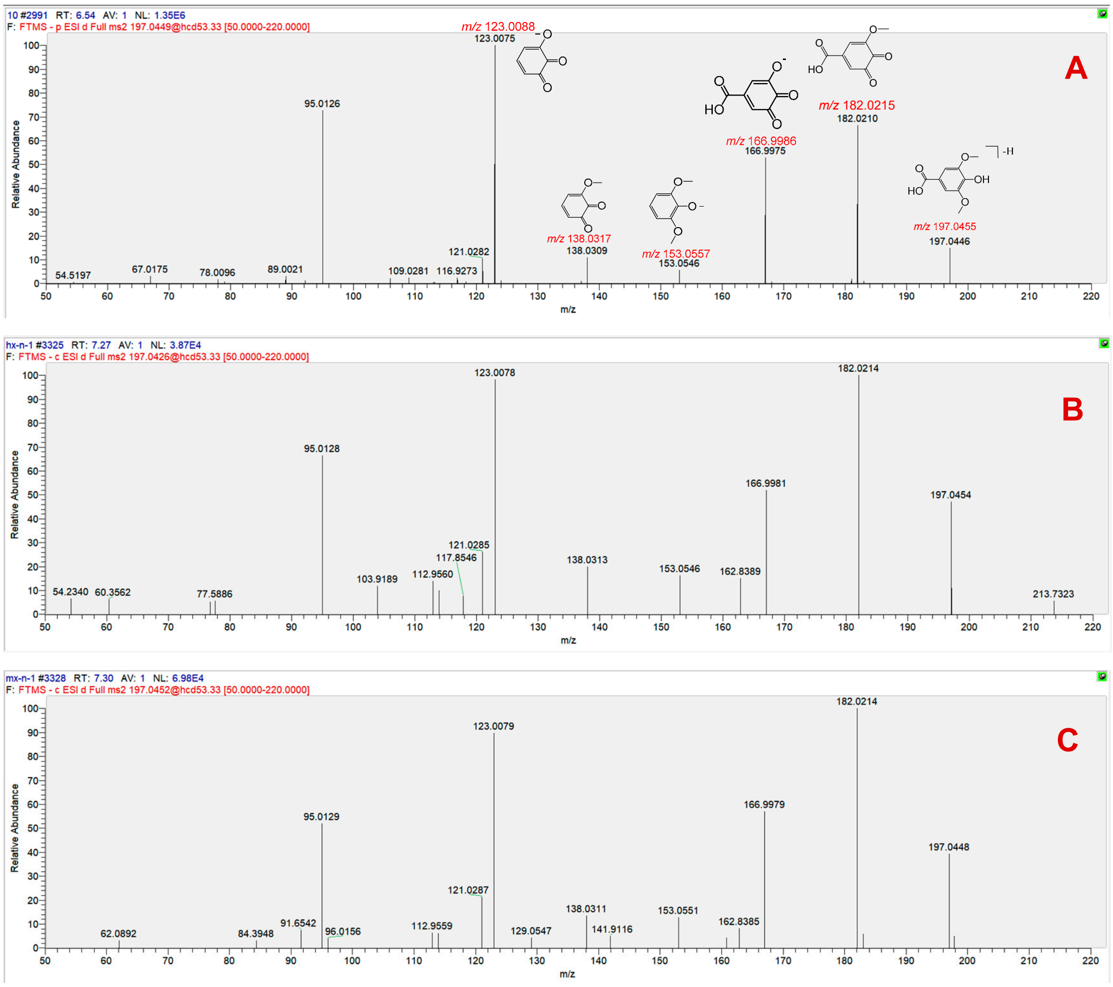


Fig. S1.26.1 The main results of syringic acid (CAS 530-57-4, $C_9H_{10}O_5$) and its corresponding peak in the TIC diagram using UHPLC-Q-Orbitrap-MS/MS analysis. (**A**) The MS/MS fragments of authentic standard syringic acid. (**B**) The MS/MS spectra from chromatographic peak in the CoCU extract. (**C**) The MS/MS spectra from chromatographic peak in the CoNU extract.

**Note**: The m/z values in red are the calculated ones. The m/z calculation was based on the relative atomic masses of C (12.0000), H (1.007825), O (15.994915)[1]

**Identification**: As seen in Fig. S1.26.1, the extract ion peak, MS/MS spectra, and characteristic pears were highly similar. Thus, the chromatographic peaks in the extracts (CoCU, CoNU) were identified as syringic acid (CAS 530-57-4).

*Suppl. 1.27* Identification of vicenin-2 (CAS 23666-13-9, $C_{27}H_{30}O_{15}$, M.W. 594.52).

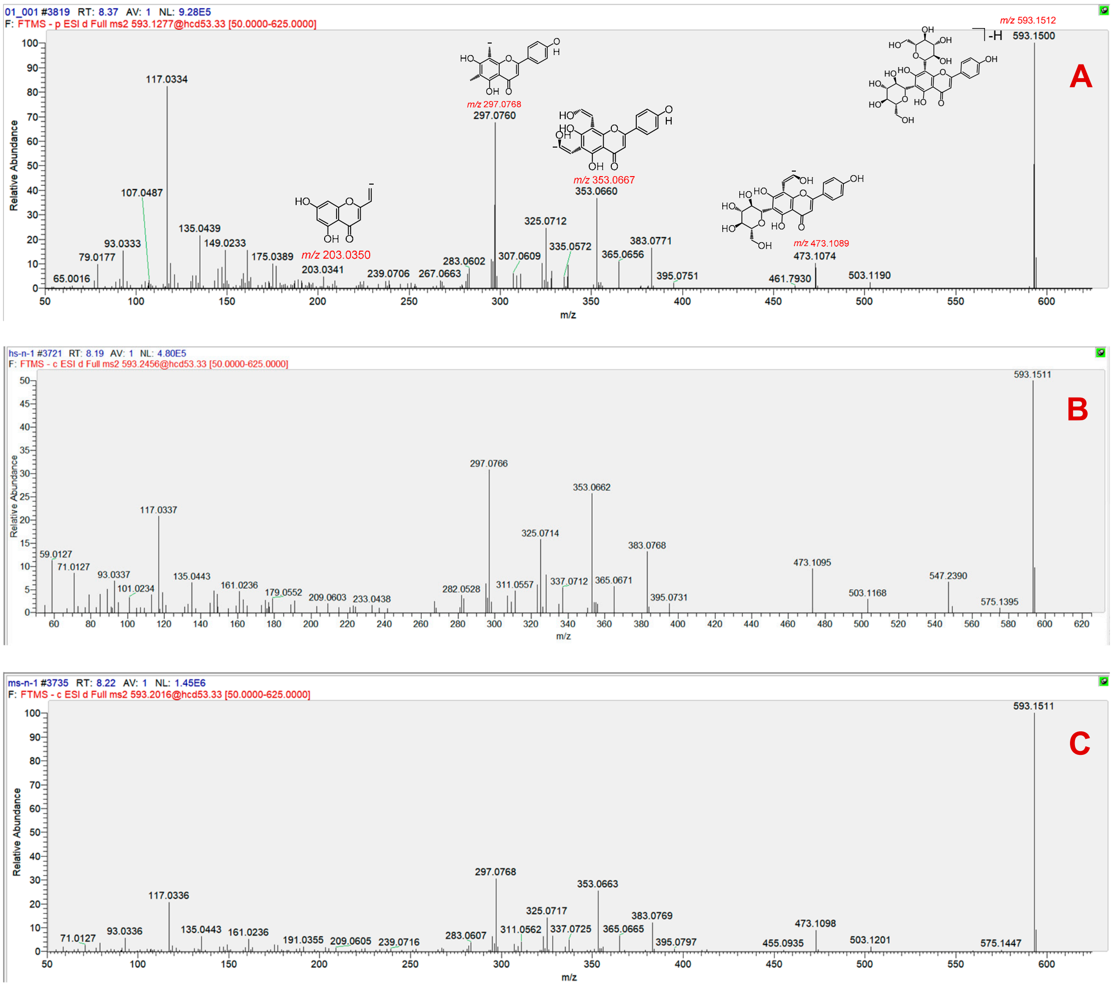


Fig. S1.27.1 The main results of vicenin-2 (CAS 23666-13-9, $C_{27}H_{30}O_{15}$) and its corresponding peak in the TIC diagram using UHPLC-Q-Orbitrap-MS/MS analysis. (**A**) The MS/MS fragments of authentic standard vicenin-2. (**B**) The MS/MS spectra from chromatographic peak in the CoCA extract. (**C**) The MS/MS spectra from chromatographic peak in the CoNA extract.

**Note**: The m/z values in red are the calculated ones. The m/z calculation was based on the relative atomic masses of C (12.0000), H (1.007825), O (15.994915)[1]

**Identification**: As seen in Fig. S1.27.1, the extract ion peak, MS/MS spectra, and characteristic pears were highly similar. Thus, the chromatographic peaks in the extracts (CoCA, CoNA) were identified as vicenin-2 (CAS 23666-13-9).

*Suppl. 1.28* Identification of (+) taxifolin (CAS 480-18-2, $C_{15}H_{12}O_7$, M.W. 304.25).

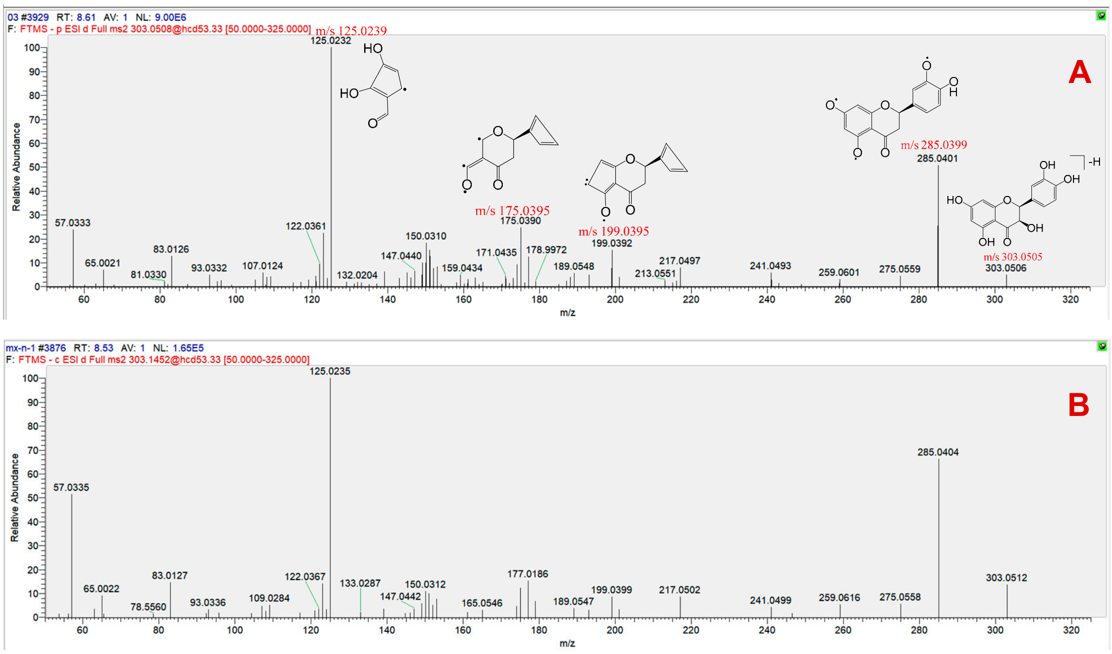


Fig. S1.28.1 The main results of (-) taxifolin (CAS 480-18-2, $C_{15}H_{12}O_7$) and its corresponding peak in the TIC diagram using UHPLC-Q-Orbitrap-MS/MS analysis. (**A**) The MS/MS fragments of authentic standard (+) taxifolin. (**B**) The MS/MS spectra from chromatographic peak in the CoNU extract.

**Note**: The m/z values in red are the calculated ones. The m/z calculation was based on the relative atomic masses of C (12.0000), H (1.007825), O (15.994915)[1]

**Identification**: As seen in Fig. S1.28.1, the extract ion peak, MS/MS spectra, and characteristic pears were highly similar. Thus, the chromatographic peaks in the extract (CoNU) were identified as (-) taxifolin (CAS 480-18-2).

*Suppl. 1.29* Identification of ferulic acid (CAS 1135-24-6, $C_{10}H_{10}O_4$, M.W. 194.19).

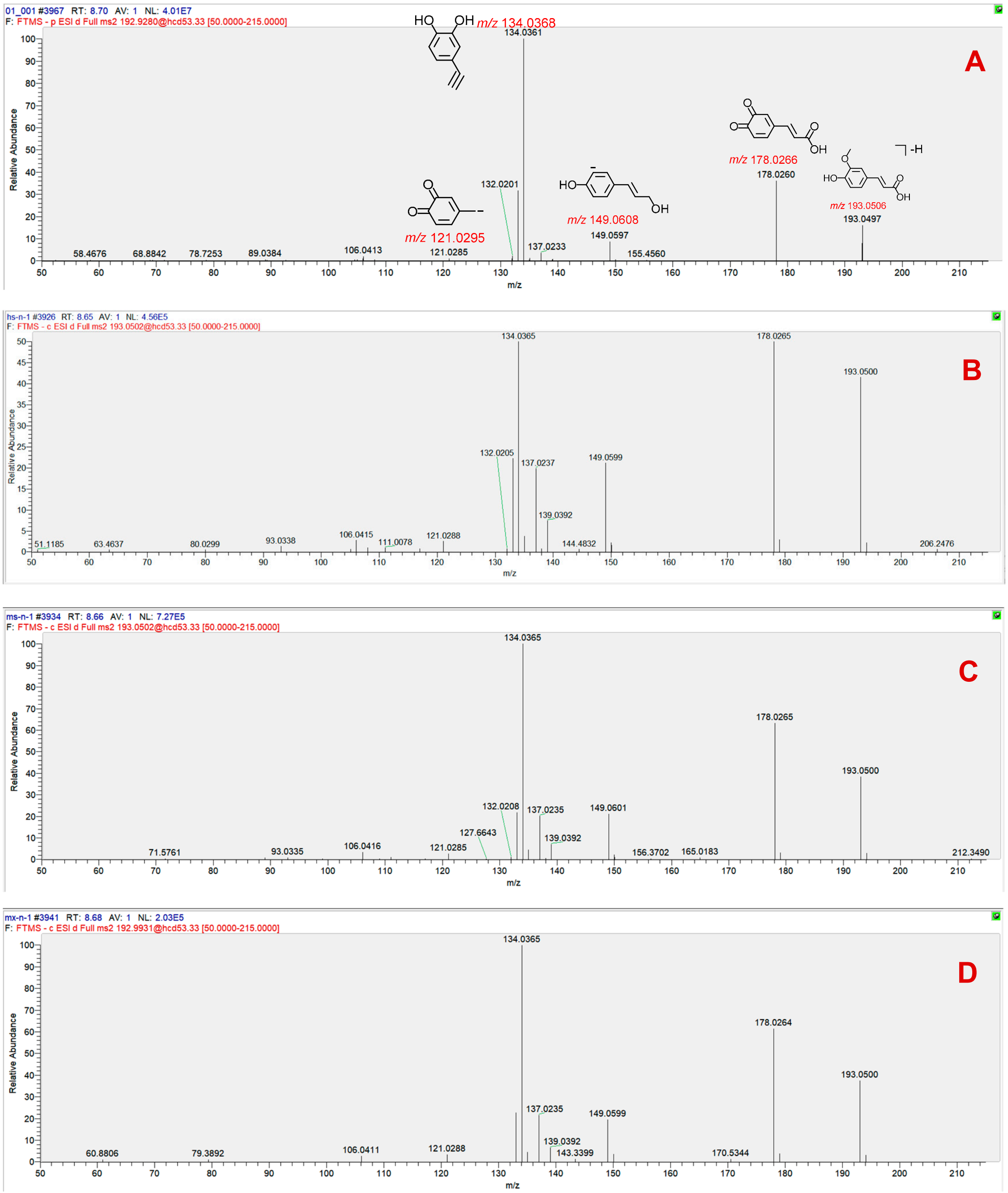


Fig. S1.29.1 The main results of ferulic acid (CAS 1135-24-6, $C_{10}H_{10}O_4$) and its corresponding peak in the TIC diagram using UHPLC-Q-Orbitrap-MS/MS analysis. (**A**) The MS/MS fragments of authentic standard ferulic acid. (**B**) The MS/MS spectra from chromatographic peak in the CoCA extract. (**C**) The MS/MS spectra from chromatographic peak in the CoNA extract. (**D**) The MS/MS spectra from chromatographic peak in the CoNU extract.

**Note**: The m/z values in red are the calculated ones. The m/z calculation was based on the relative atomic masses of C (12.0000), H (1.007825), O (15.994915)[1]

**Identification**: As seen in Fig. S1.29.1, the extract ion peak, MS/MS spectra, and characteristic pears were highly similar. Thus, the chromatographic peaks in the extracts (CoCA, CoNA, CoNU) were identified as ferulic acid (CAS 1135-24-6).

*Suppl. 1.30* Identification of orientin (CAS 28608-75-5, $C_{21}H_{20}O_{11}$, M.W. 448.38).

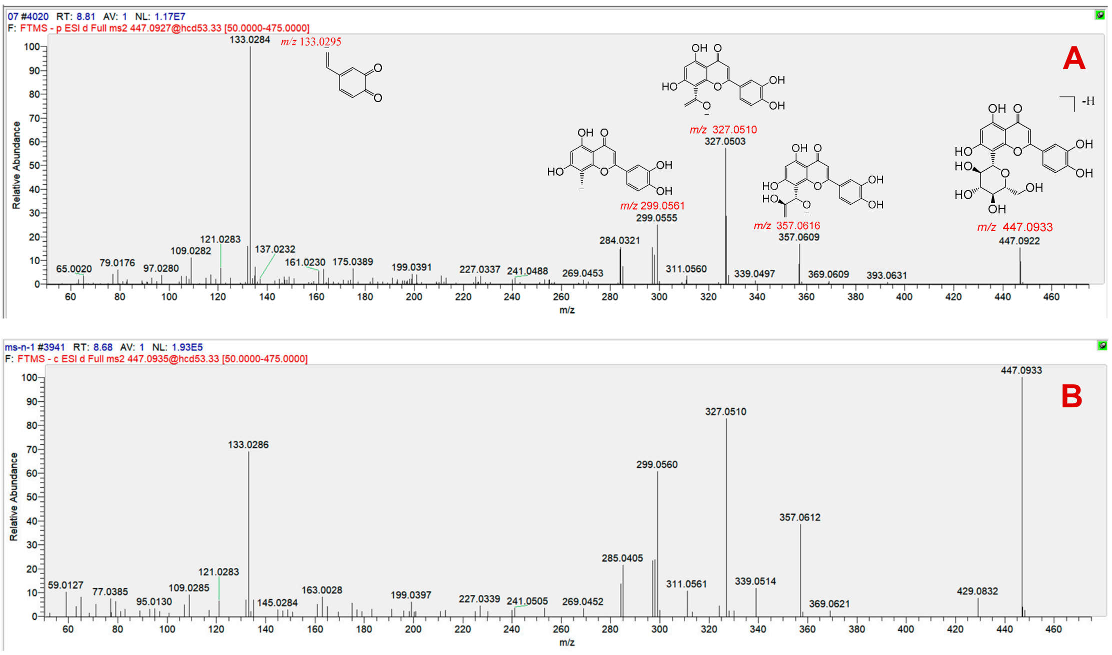


Fig. S1.30.1 The main results of orientin (CAS 28608-75-5, $C_{21}H_{20}O_{11}$) and its corresponding peak in the TIC diagram using UHPLC-Q-Orbitrap-MS/MS analysis. (**A**) The MS/MS fragments of authentic standard orientin. (**B**) The MS/MS spectra from chromatographic peak in the CoNA extract.

**Note**: The m/z values in red are the calculated ones. The m/z calculation was based on the relative atomic masses of C (12.0000), H (1.007825), O (15.994915)[1]

**Identification**: As seen in Fig. S1.30.1, the extract ion peak, MS/MS spectra, and characteristic pears were highly similar. Thus, the chromatographic peaks in the extract (CoNA) were identified as orientin (CAS 28608-75-5).

*Suppl. 1.31* Identification of eriodictyol 7-*O*-glucoside (CAS 38965-51-4, $C_{21}H_{22}O_{11}$, M.W. 450.39).

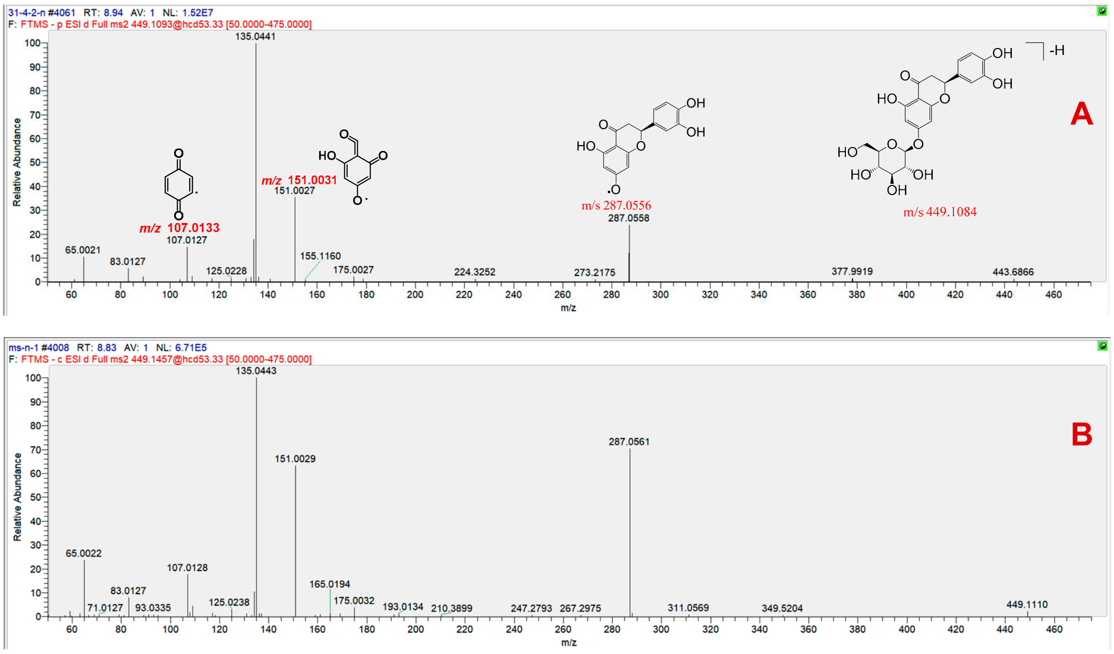


Fig. S1.31.1The main results of eriodictyol 7-*O*-glucoside (CAS 38965-51-4, $C_{21}H_{22}O_{11}$) and its corresponding peak in the TIC diagram using UHPLC-Q-Orbitrap-MS/MS analysis. (**A**) The MS/MS fragments of authentic standard eriodictyol 7-*O*-glucoside. (**B**) The MS/MS spectra from chromatographic peak in the CoNA extract.

**Note**: The m/z values in red are the calculated ones. The m/z calculation was based on the relative atomic masses of C (12.0000), H (1.007825), O (15.994915)[1]

**Identification**: As seen in Fig. S1.31.1, the extract ion peak, MS/MS spectra, and characteristic pears were highly similar. Thus, the chromatographic peaks in the extract (CoNA) were identified as eriodictyol 7-*O*-glucoside (CAS 38965-51-4).

*Suppl. 1.32* Identification of 5,7-dihydroxychromone (CAS 31721-94-5, $C_9H_6O_4$, M.W. 178.14 ).

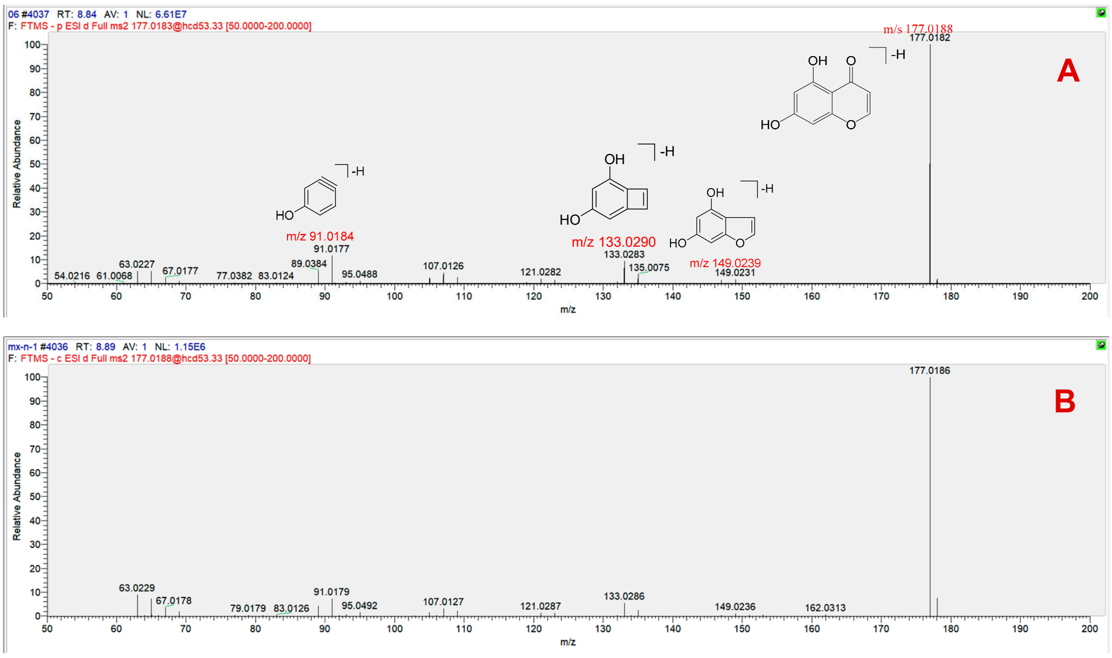


Fig. S1.32.1 The main results of 5,7-dihydroxychromone (CAS 31721-94-5, $C_9H_6O_4$) and its corresponding peak in the TIC diagram using UHPLC-Q-Orbitrap-MS/MS analysis. (**A**) The MS/MS fragments of authentic standard 5,7-dihydroxychromone. (**B**) The MS/MS spectra from chromatographic peak in the CoNU extract.

**Note**: The m/z values in red are the calculated ones. The m/z calculation was based on the relative atomic masses of C (12.0000), H (1.007825), O (15.994915)[1]

**Identification**: As seen in Fig. S1.32.1, the extract ion peak, MS/MS spectra, and characteristic pears were highly similar. Thus, the chromatographic peaks in the extract (CoNU) were identified as 5,7-dihydroxychromone (CAS 31721-94-5).

*Suppl. 1.33* Identification of coniferaldehyde (CAS 20649-42-7, $C_{10}H_{10}O_3$, M.W. 178.18).

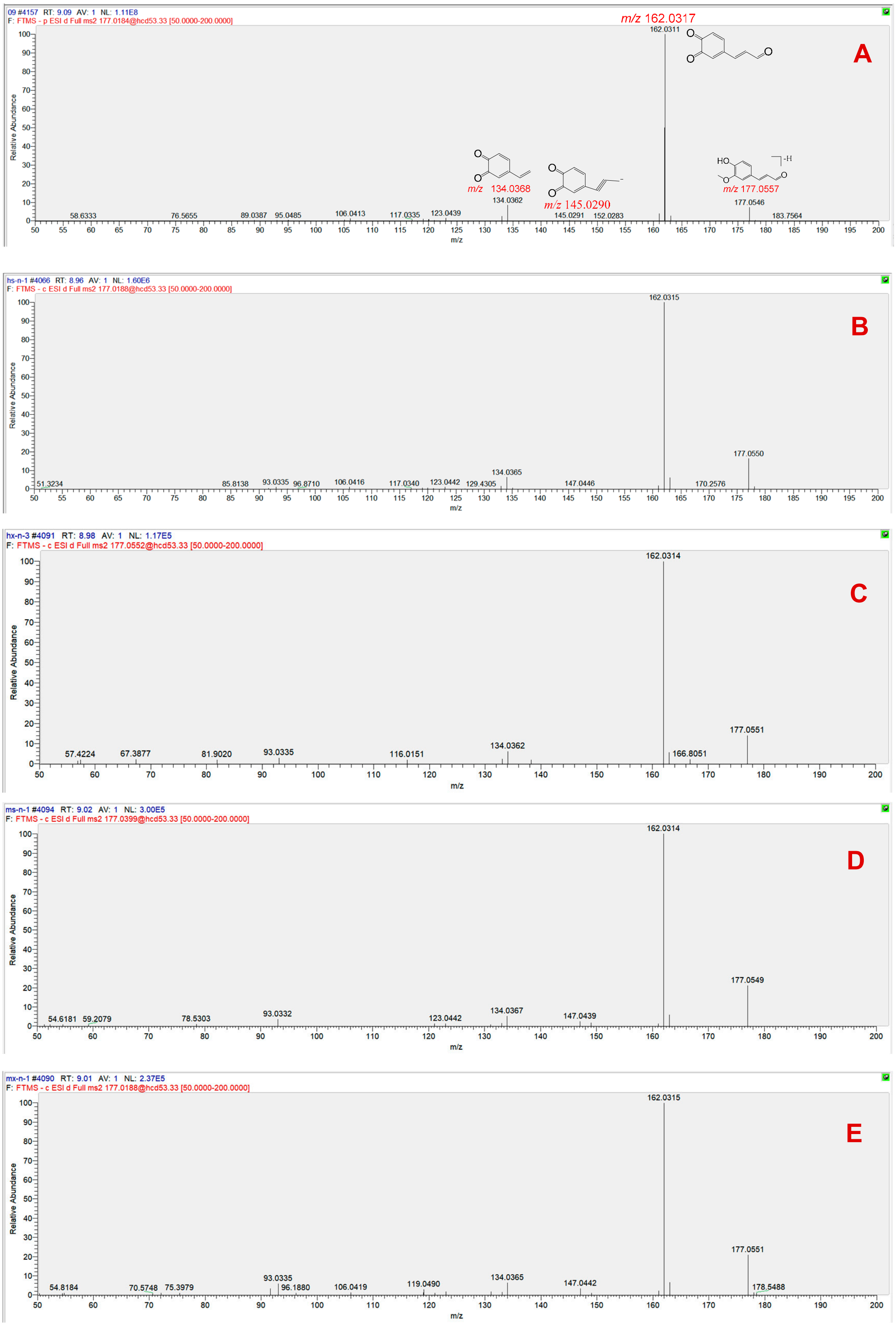


Fig. S1.33.1 The main results of coniferaldehyde (CAS 20649-42-7, $C_{10}H_{10}O_3$) and its corresponding peak in the TIC diagram using UHPLC-Q-Orbitrap-MS/MS analysis. (**A**) The MS/MS fragments of

authentic standard coniferaldehyde. (**B**) The MS/MS spectra from chromatographic peak in the CoCA extract. (**C**) The MS/MS spectra from chromatographic peak in the CoCU extract. (**D**) The MS/MS spectra from chromatographic peak in the CoNA extract. (**E**) The MS/MS spectra from chromatographic peak in the CoNU extract.

**Note**: The m/z values in red are the calculated ones. The m/z calculation was based on the relative atomic masses of C (12.0000), H (1.007825), O (15.994915)[1]

**Identification**: As seen in Fig. S1.33.1, the extract ion peak, MS/MS spectra, and characteristic pears were highly similar. Thus, the chromatographic peaks in the extracts (CoCA, CoCU, CoNA, CoNU) were identified as coniferaldehyde (CAS 20649-42-7).

*Suppl. 1.34* Identification of isochlorogenic acid B (CAS 14534-61-3, $C_{25}H_{24}O_{12}$, M.W.

516.455).

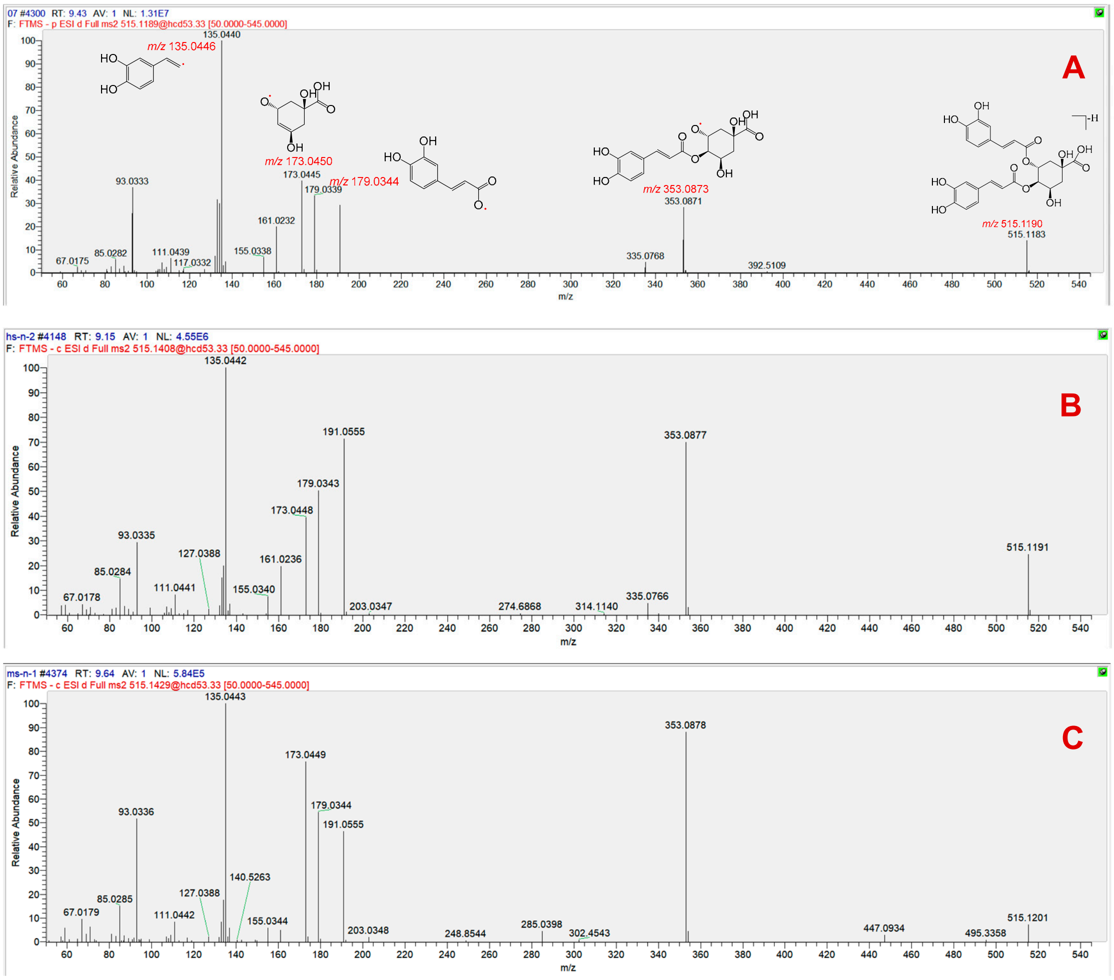


Fig. S1.34.1The main results of isochlorogenic acid B (CAS 14534-61-3, $C_{25}H_{24}O_{12}$) and its corresponding peak in the TIC diagram using UHPLC-Q-Orbitrap-MS/MS analysis. (**A**) The MS/MS fragments of authentic standard isochlorogenic acid B. (**B**) The MS/MS spectra from chromatographic peak in the CoCA extract. (**C**) The MS/MS spectra from chromatographic peak in the CoNA extract.

**Note**: The m/z values in red are the calculated ones. The m/z calculation was based on the relative atomic masses of C (12.0000), H (1.007825), O (15.994915)[1]

**Identification**: As seen in Fig. S1.34.1, the extract ion peak, MS/MS spectra, and characteristic pears were highly similar. Thus, the chromatographic peaks in the extracts (CoCA, CoNA) were identified as isochlorogenic acid B (CAS 14534-61-3).

*Suppl. 1.35* Identification of quercetin 3-*O*- β -*D*-glucuronide (CAS 22688-79-5,

$C_{21}H_{18}O_{13}$, M.W. 478.36 ).

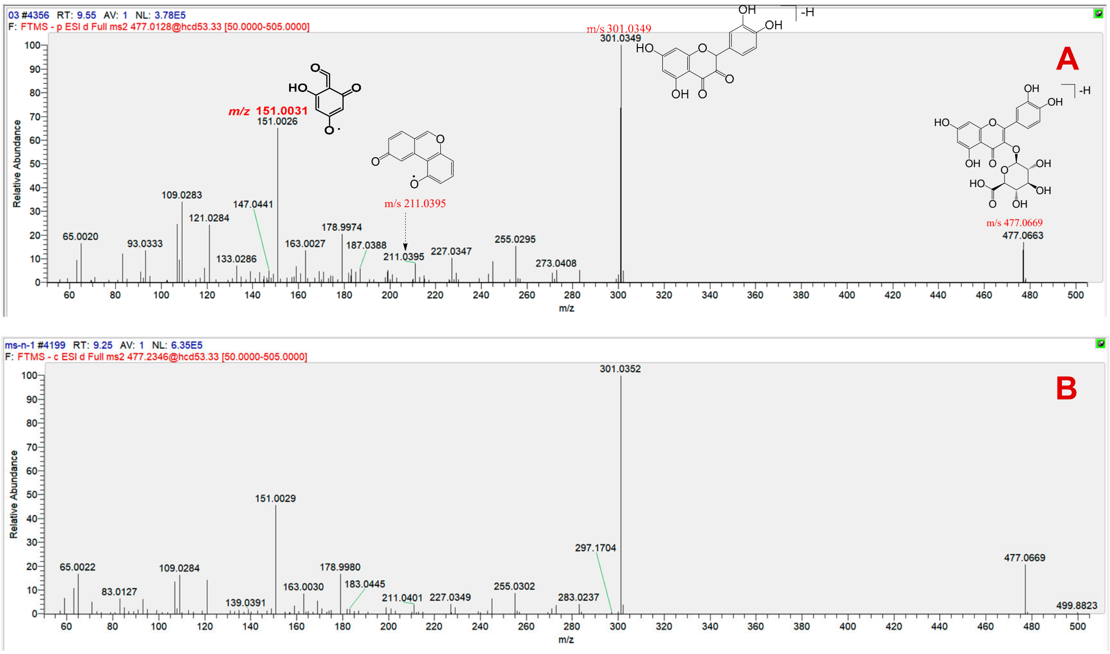


Fig. S1.35.1 The main results of quercetin 3-*O*-*β*-*D*-glucuronide (CAS 22688-79-5, $C_{21}H_{18}O_{13}$) and its corresponding peak in the TIC diagram using UHPLC-Q-Orbitrap-MS/MS analysis. (**A**) The MS/MS fragments of authentic standard quercetin 3-*O*-*β*-*D*-glucuronide. (**B**) The MS/MS spectra from chromatographic peak in the CoNA extract.

**Note**: The m/z values in red are the calculated ones. The m/z calculation was based on the relative atomic masses of C (12.0000), H (1.007825), O (15.994915)[1]

**Identification**: As seen in Fig. S1.35.1, the extract ion peak, MS/MS spectra, and characteristic pears were highly similar. Thus, the chromatographic peaks in the extract (CoNA) were identified as quercetin 3-O-β-D-glucuronide (CAS 22688-79-5).

*Suppl. 1.36* Identification of luteoloside (CAS 5373-11-5, $C_{21}H_{20}O_{11}$, M.W. 448.38).

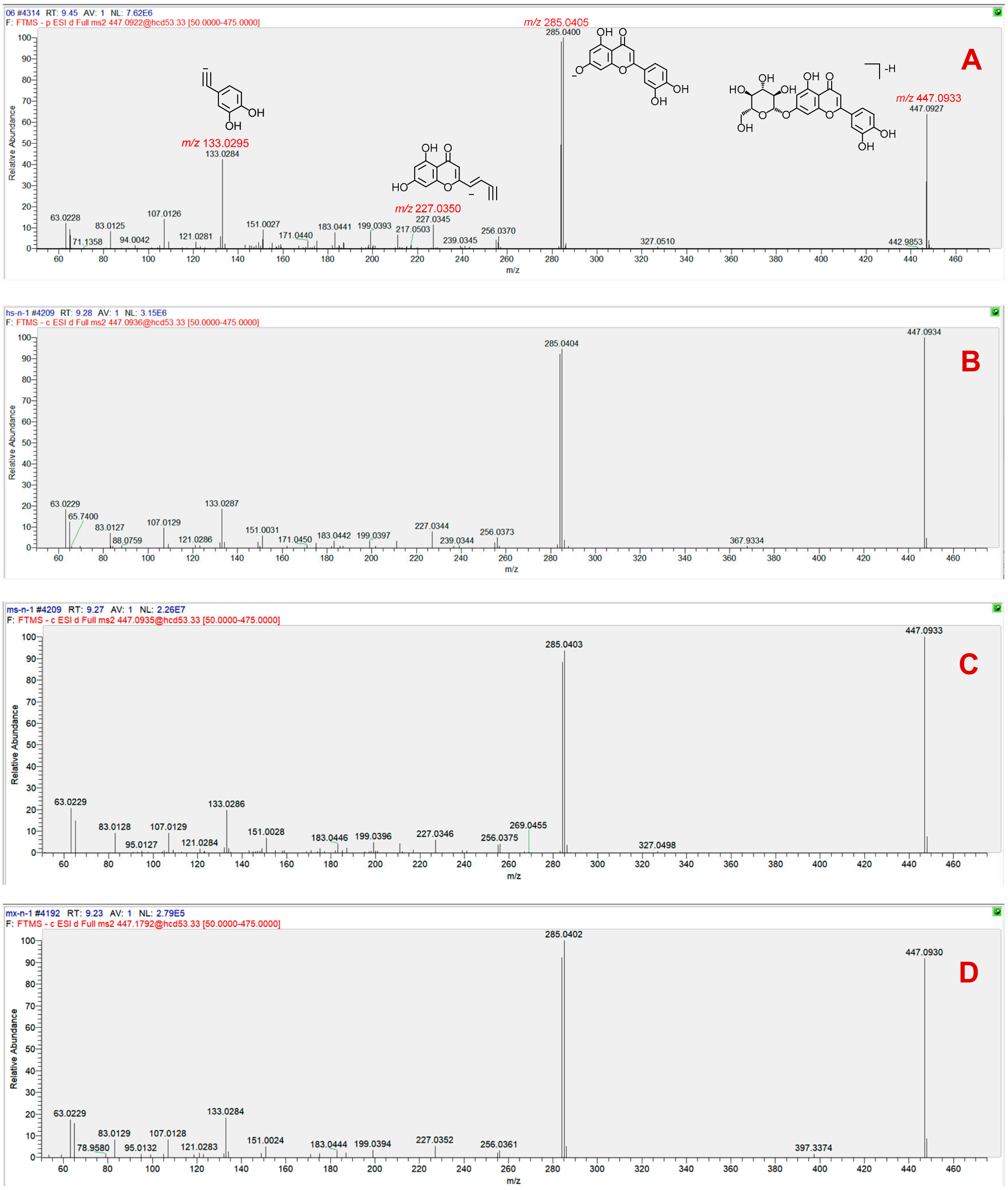


Fig. S1.36.1 The main results of luteoloside (CAS 5373-11-5, $C_{21}H_{20}O_{11}$) and its corresponding peak in the TIC diagram using UHPLC-Q-Orbitrap-MS/MS analysis. (**A**) The MS/MS fragments of authentic standard luteoloside. (**B**) The MS/MS spectra from chromatographic peak in the CoCA extract. (**C**) The MS/MS spectra from chromatographic peak in the CoNA extract. (**D**) The MS/MS spectra from chromatographic peak in the CoNU extract.

**Note**: The m/z values in red are the calculated ones. The m/z calculation was based on the relative atomic masses of C (12.0000), H (1.007825), O (15.994915)[1]

**Identification**: As seen in Fig. S1.36.1, the extract ion peak, MS/MS spectra, and characteristic pears were highly similar. Thus, the chromatographic peaks in the extracts (CoCA, CoNA, CoNU) were identified as luteoloside (CAS 5373-11-5).

*Suppl. 1.37* Identification of eleutheroside E1 (CAS 7374-79-0, $C_{28}H_{36}O_{13}$, M.W.

580.6).

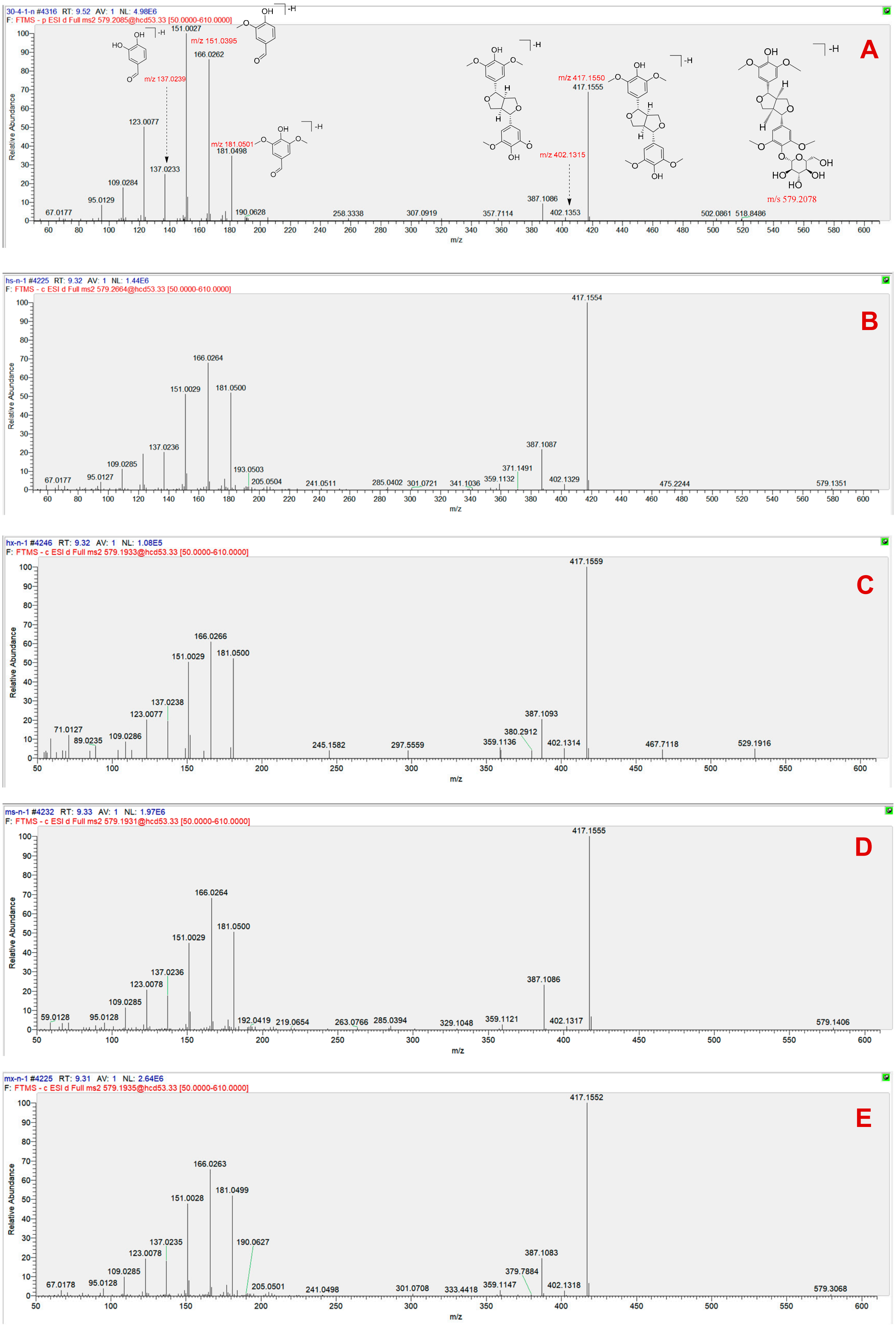


Fig. S1.37.1 The main results of eleutheroside E1 (CAS 7374-79-0, $C_{28}H_{36}O_{13}$) and its corresponding peak in the TIC diagram using UHPLC-Q-Orbitrap-MS/MS analysis. (**A**) The MS/MS fragments of authentic standard eleutheroside E1. (**B**) The MS/MS spectra from chromatographic peak in the CoCA

extract. (**C**) The MS/MS spectra from chromatographic peak in the CoCU extract. (**D**) The MS/MS spectra from chromatographic peak in the CoNA extract. (**E**) The MS/MS spectra from chromatographic peak in the CoNU extract.

**Note**: The m/z values in red are the calculated ones. The m/z calculation was based on the relative atomic masses of C (12.0000), H (1.007825), O (15.994915)[1]

**Identification**: As seen in Fig. S1.37.1, the extract ion peak, MS/MS spectra, and characteristic pears were highly similar. Thus, the chromatographic peaks in the extracts (CoCA, CoCU, CoNA, CoNU) were identified as eleutheroside E1 (CAS 7374-79-0).

*Suppl. 1.38* Identification of hyperoside (CAS 482-36-0, $C_{21}H_{20}O_{12}$, M.W. 464.379).

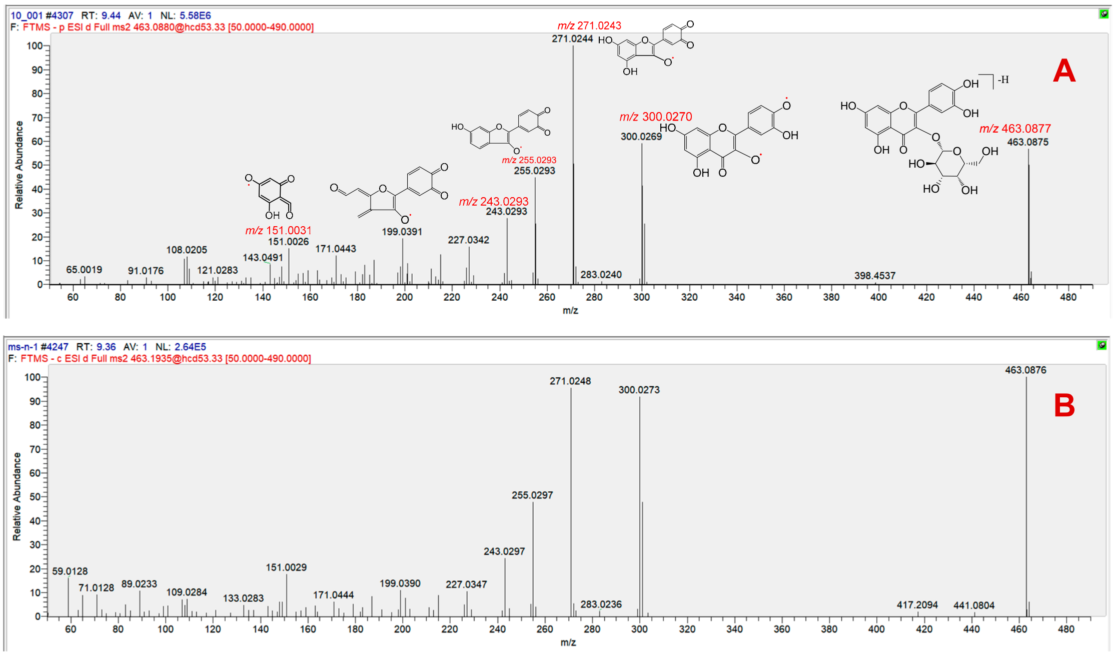


Fig. S1.38.1 The main results of hyperoside (CAS 482-36-0, $C_{21}H_{20}O_{12}$) and its corresponding peak in the TIC diagram using UHPLC-Q-Orbitrap-MS/MS analysis. (**A**) The MS/MS fragments of authentic standard hyperoside. (**B**) The MS/MS spectra from chromatographic peak in the CoNA extract.

**Note**: The m/z values in red are the calculated ones. The m/z calculation was based on the relative atomic masses of C (12.0000), H (1.007825), O (15.994915)[1]

**Identification**: As seen in Fig. S1.38.1, the extract ion peak, MS/MS spectra, and characteristic pears were highly similar. Thus, the chromatographic peaks in the extract (CoNA) were identified as hyperoside (CAS 482-36-0).

*Suppl. 1.39* Identification of *S*-naringenin-7-*O*-β-*D*-glucoside (CAS 529-55-5, $C_{21}H_{22}O_{10}$, M.W. 434.39).

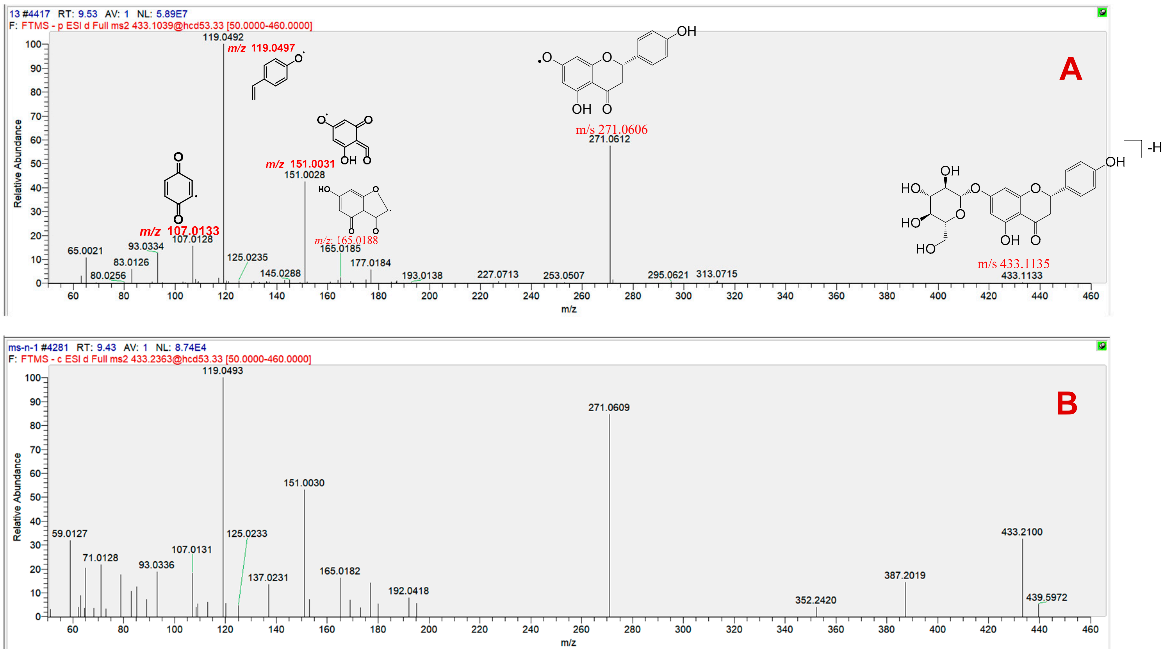


Fig. S1.39.1 The main results of *S*-naringenin-7-*O*-*β*-*D*-glucoside (CAS 529-55-5, $C_{21}H_{22}O_{10}$) and its corresponding peak in the TIC diagram using UHPLC-Q-Orbitrap-MS/MS analysis. (**A**) The MS/MS fragments of authentic standard *S*-naringenin-7-*O*-*β*-*D*-glucoside. (**B**) The MS/MS spectra from chromatographic peak in the CoNA extract.

**Note**: The m/z values in red are the calculated ones. The m/z calculation was based on the relative atomic masses of C (12.0000), H (1.007825), O (15.994915)[1]

**Identification**: As seen in Fig. S1.39.1, the extract ion peak, MS/MS spectra, and characteristic pears were highly similar. Thus, the chromatographic peaks in the extract (CoNA) were identified as *S*-naringenin-7-*O*-*β*-*D*-glucoside (CAS 529-55-5).

*Suppl. 1.40* Identification of isochlorogenic acid C (CAS 57378-72-0, $C_{25}H_{24}O_{12}$, M.W. 516.45).

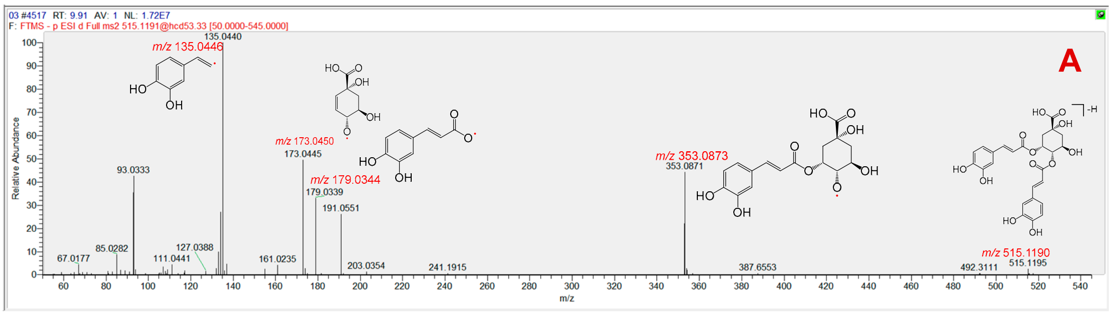


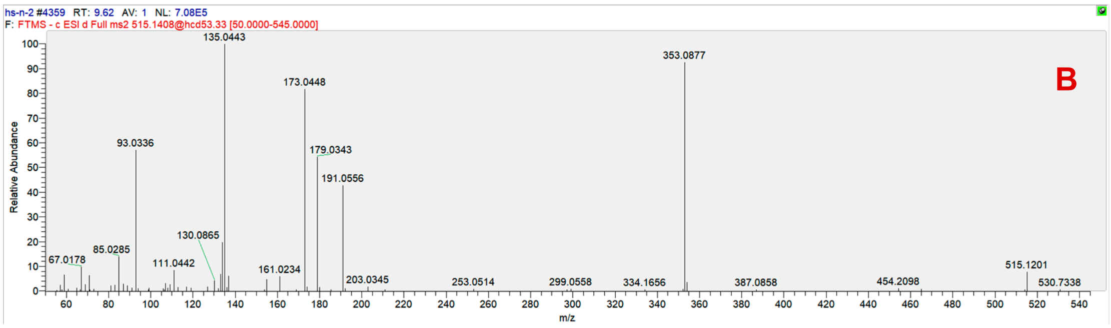


Fig. S1.40.1 The main results of isochlorogenic acid C (CAS 57378-72-0, $C_{25}H_{24}O_{12}$) and its corresponding peak in the TIC diagram using UHPLC-Q-Orbitrap-MS/MS analysis. (**A**) The MS/MS fragments of authentic standard isochlorogenic acid C. (**B**) The MS/MS spectra from chromatographic peak in the CoCA extract.

**Note**: The m/z values in red are the calculated ones. The m/z calculation was based on the relative atomic masses of C (12.0000), H (1.007825), O (15.994915)[1]

**Identification**: As seen in Fig. S1.40.1, the extract ion peak, MS/MS spectra, and characteristic pears were highly similar. Thus, the chromatographic peaks in the extract (CoCA) were identified as isochlorogenic acid C (CAS 57378-72-0).

*Suppl. 1.41* Identification of p-cresol (CAS 106-44-5, $C_7H_8O$, M.W. 108.13).

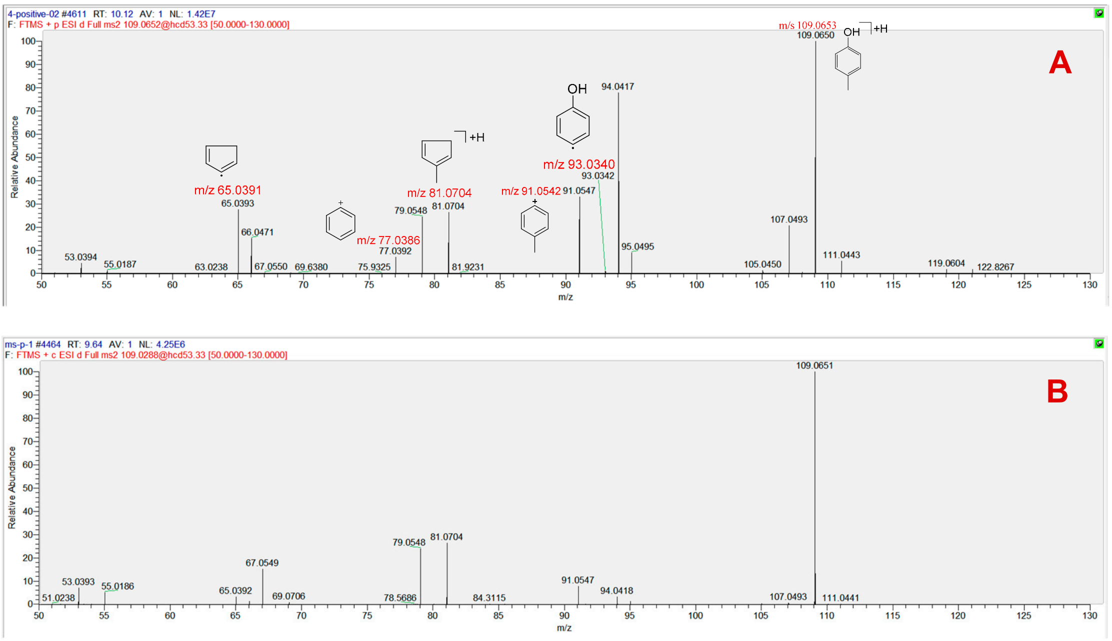


Fig. S1.41.1 The main results of p-cresol (CAS 106-44-5, $C_7H_8O$) and its corresponding peak in the TIC diagram using UHPLC-Q-Orbitrap-MS/MS analysis. (**A**) The MS/MS fragments of authentic standard p-cresol. (**B**) The MS/MS spectra from chromatographic peak in the CoNA extract.

**Note**: The m/z values in red are the calculated ones. The m/z calculation was based on the relative atomic masses of C (12.0000), H (1.007825), O (15.994915)[1]

**Identification**: As seen in Fig. S1.41.1, the extract ion peak, MS/MS spectra, and characteristic pears were highly similar. Thus, the chromatographic peaks in the extract (CoNA) were identified as p-cresol (CAS 106-44-5).

*Suppl. 1.42* Identification of apigenin 7-*O*-glucoside (CAS 578-74-5, $C_{21}H_{20}O_{10}$, M.W. 432.381).

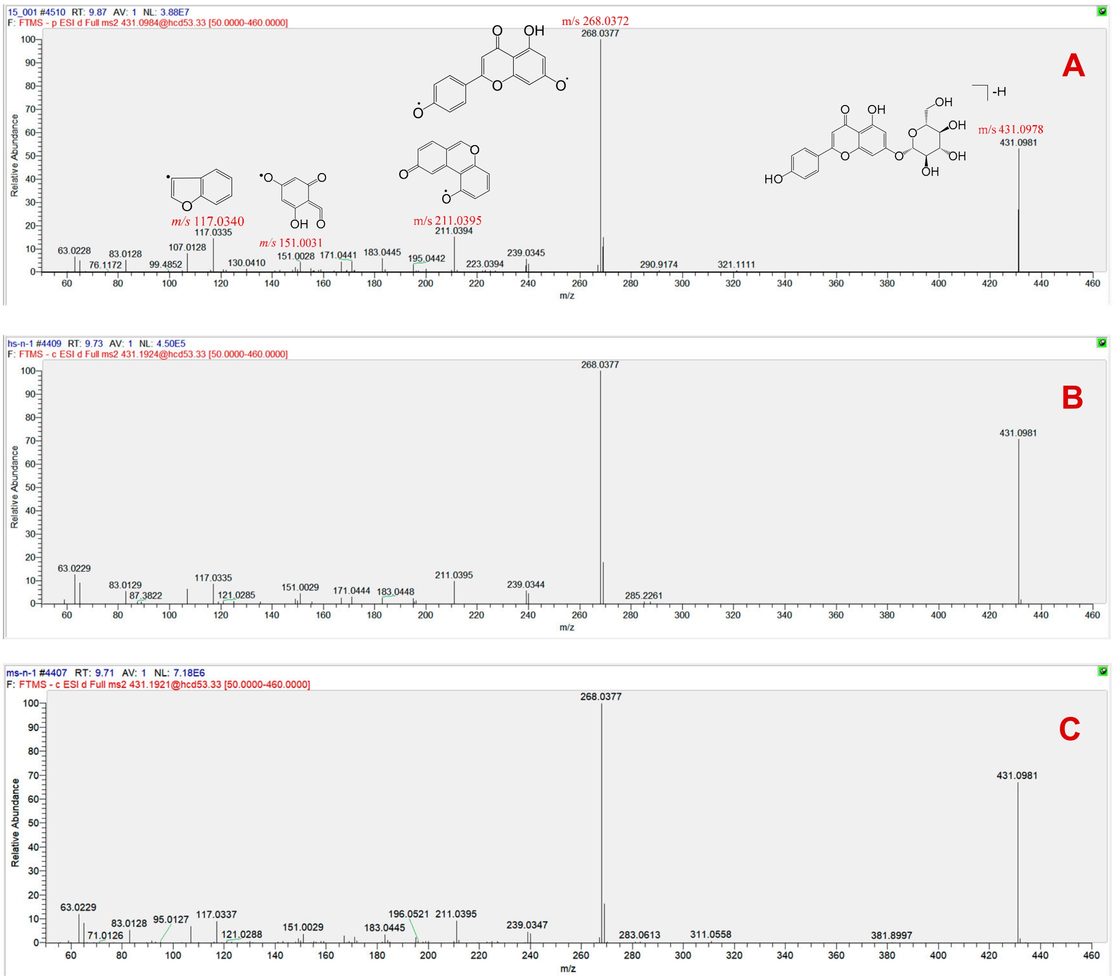

Fig. S1.42.1 The main results of apigenin 7-*O*-glucoside (CAS 578-74-5, $C_{21}H_{20}O_{10}$) and its corresponding peak in the TIC diagram using UHPLC-Q-Orbitrap-MS/MS analysis. (**A**) The MS/MS fragments of authentic standard apigenin 7-*O*-glucoside. (**B**) The MS/MS spectra from chromatographic peak in the CoCA extract. (**C**) The MS/MS spectra from chromatographic peak in the CoNA extract.

**Note**: The m/z values in red are the calculated ones. The m/z calculation was based on the relative atomic masses of C (12.0000), H (1.007825), O (15.994915)[1]

**Identification**: As seen in Fig. S1.42.1, the extract ion peak, MS/MS spectra, and characteristic pears were highly similar. Thus, the chromatographic peaks in the extracts (CoCA, CoNA) were identified as apigenin 7-*O*-glucoside (CAS 578-74-5).

*Suppl. 1.43* Identification of isochlorogenic acid A (CAS 2450-53-5, $C_{25}H_{24}O_{12}$, M.W. 516.45 ).

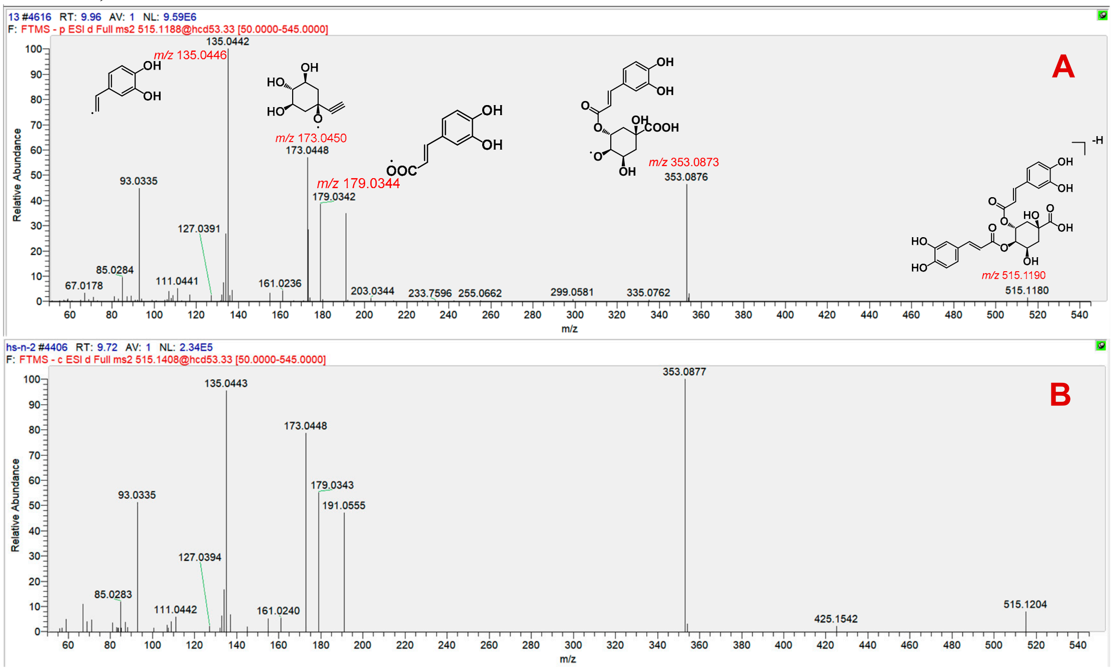


Fig. S1.43.1 The main results of isochlorogenic acid A (CAS 2450-53-5, $C_{25}H_{24}O_{12}$) and its corresponding peak in the TIC diagram using UHPLC-Q-Orbitrap-MS/MS analysis. (**A**) The MS/MS fragments of authentic standard isochlorogenic acid A. (**B**) The MS/MS spectra from chromatographic peak in the CoCA extract.

**Note**: The m/z values in red are the calculated ones. The m/z calculation was based on the relative atomic masses of C (12.0000), H (1.007825), O (15.994915)[1]

**Identification**: As seen in Fig. S1.43.1, the extract ion peak, MS/MS spectra, and characteristic pears were highly similar. Thus, the chromatographic peaks in the extract (CoCA) were identified as isochlorogenic acid A (CAS 2450-53-5).

*Suppl. 1.44* Identification of okanin (CAS 484-76-4, $C_{15}H_{12}O_6$, M.W. 288.3).

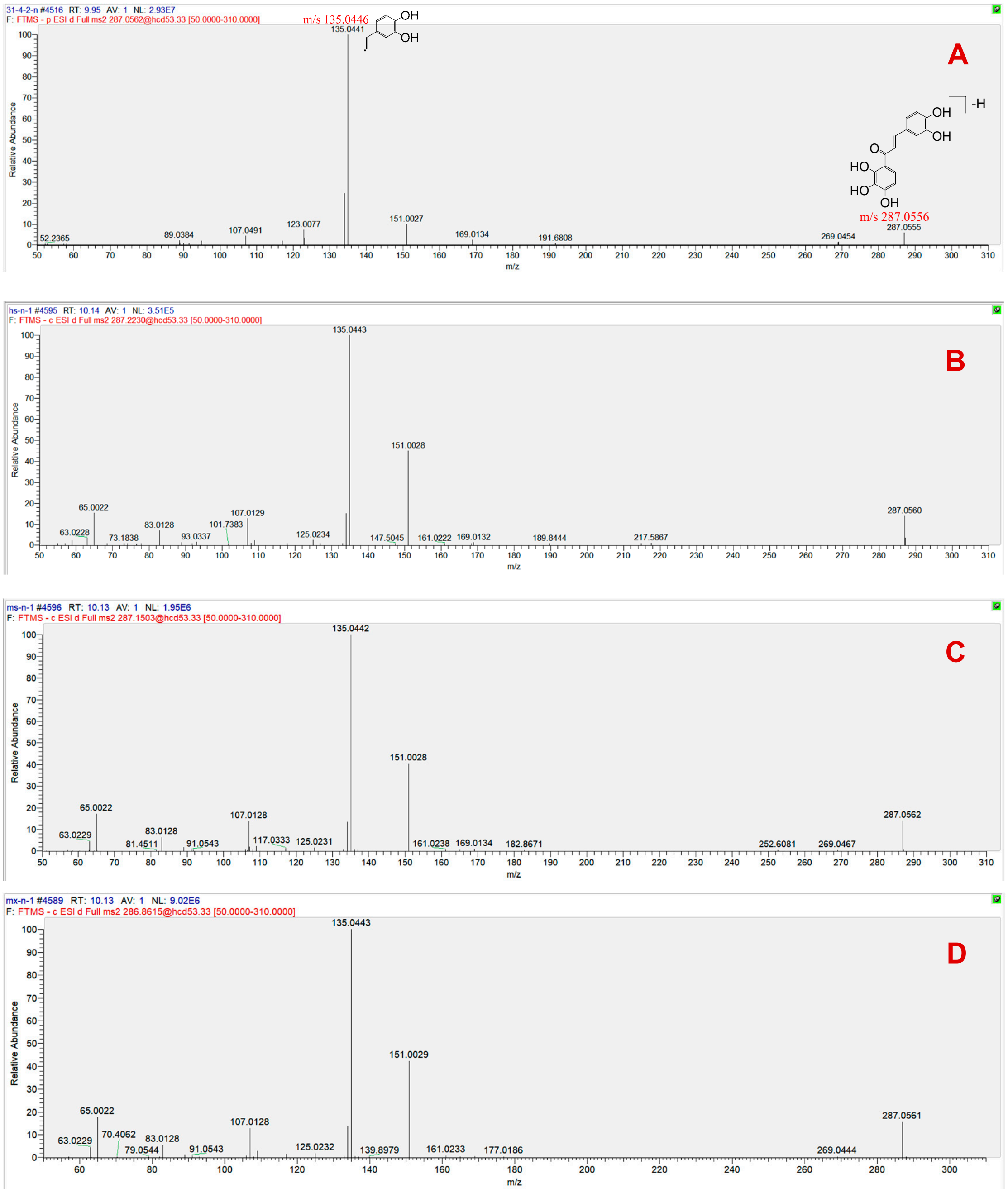


Fig. S1.44.1 The main results of okanin (CAS 484-76-4, $C_{15}H_{12}O_6$) and its corresponding peak in the TIC diagram using UHPLC-Q-Orbitrap-MS/MS analysis. (**A**) The MS/MS fragments of authentic standard okanin. (**B**) The MS/MS spectra from chromatographic peak in the CoCA extract. (**C**) The MS/MS spectra from chromatographic peak in the CoNA extract. (**D**) The MS/MS spectra from chromatographic peak in the CoNU extract.

**Note**: The m/z values in red are the calculated ones. The m/z calculation was based on the relative atomic masses of C (12.0000), H (1.007825), O (15.994915)[1]

**Identification**: As seen in Fig. S1.44.1, the extract ion peak, MS/MS spectra, and characteristic pears were highly similar. Thus, the chromatographic peaks in the extracts (CoCA, CoNA, CoNU) were identified as okanin (CAS 484-76-4).

*Suppl. 1.45* Identification of lobetyolin (CAS 136085-37-5, $C_{20}H_{28}O_8$, M.W. 396.4).

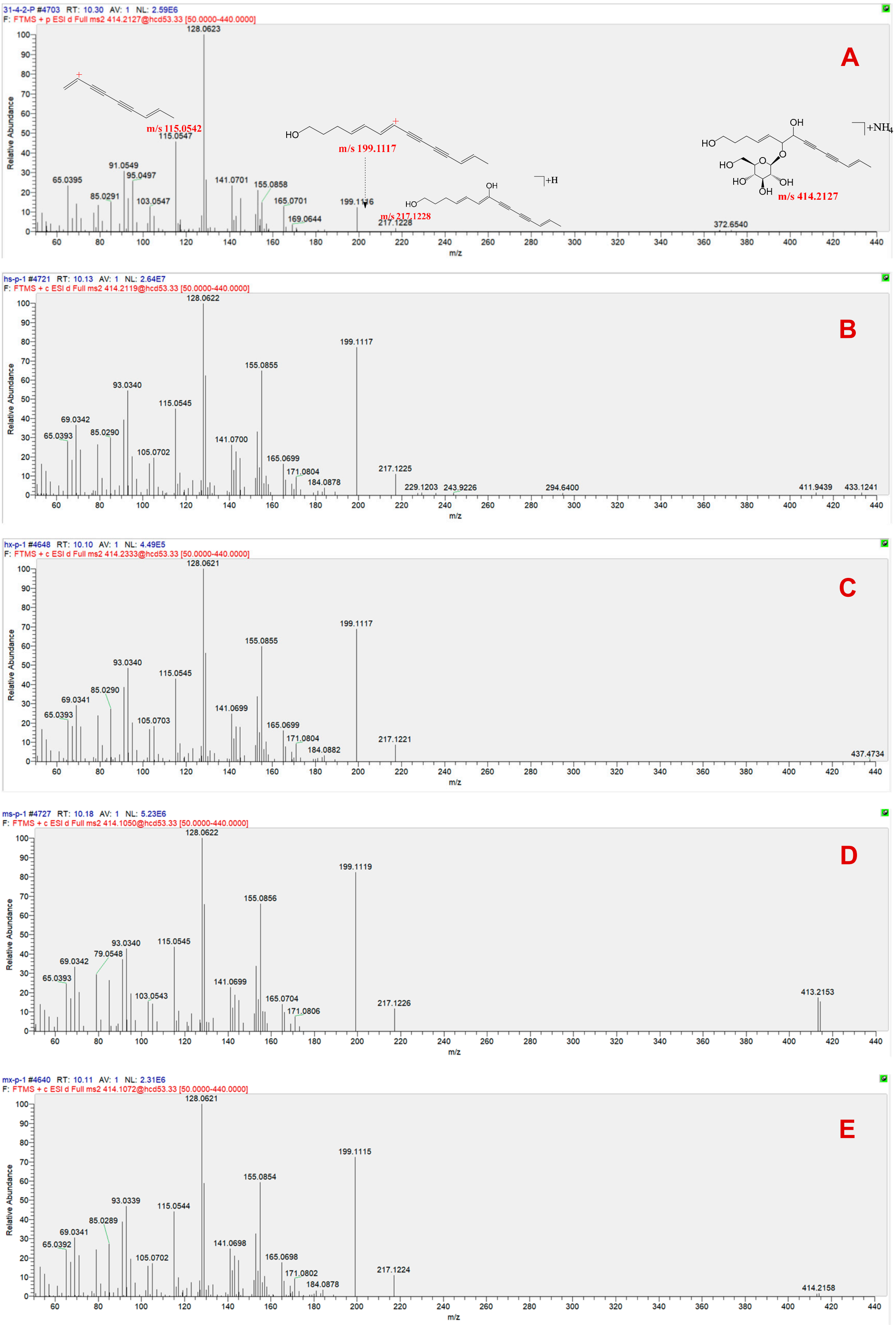


Fig. S1.45.1 The main results of lobetyolin (CAS 136085-37-5, $C_{20}H_{28}O_8$) and its corresponding peak in the TIC diagram using UHPLC-Q-Orbitrap-MS/MS analysis. (**A**) The MS/MS fragments of authentic standard lobetyolin. (**B**) The MS/MS spectra from chromatographic peak in the CoCA

extract. (**C**) The MS/MS spectra from chromatographic peak in the CoCU extract. (**D**) The MS/MS spectra from chromatographic peak in the CoNA extract. (**E**) The MS/MS spectra from chromatographic peak in the CoNU extract.

**Note**: The m/z values in red are the calculated ones. The m/z calculation was based on the relative atomic masses of C (12.0000), H (1.007825), O (15.994915)[1]

**Identification**: As seen in Fig. S1.45.1, the extract ion peak, MS/MS spectra, and characteristic pears were highly similar. Thus, the chromatographic peaks in the extracts (CoCA, CoCU, CoNA, CoNU) were identified as lobetyolin (CAS 136085-37-5).

*Suppl. 1.46* Identification of viscidulin I (CAS 92519-95-4, $C_{15}H_{10}O_7$, M.W. 302.24).

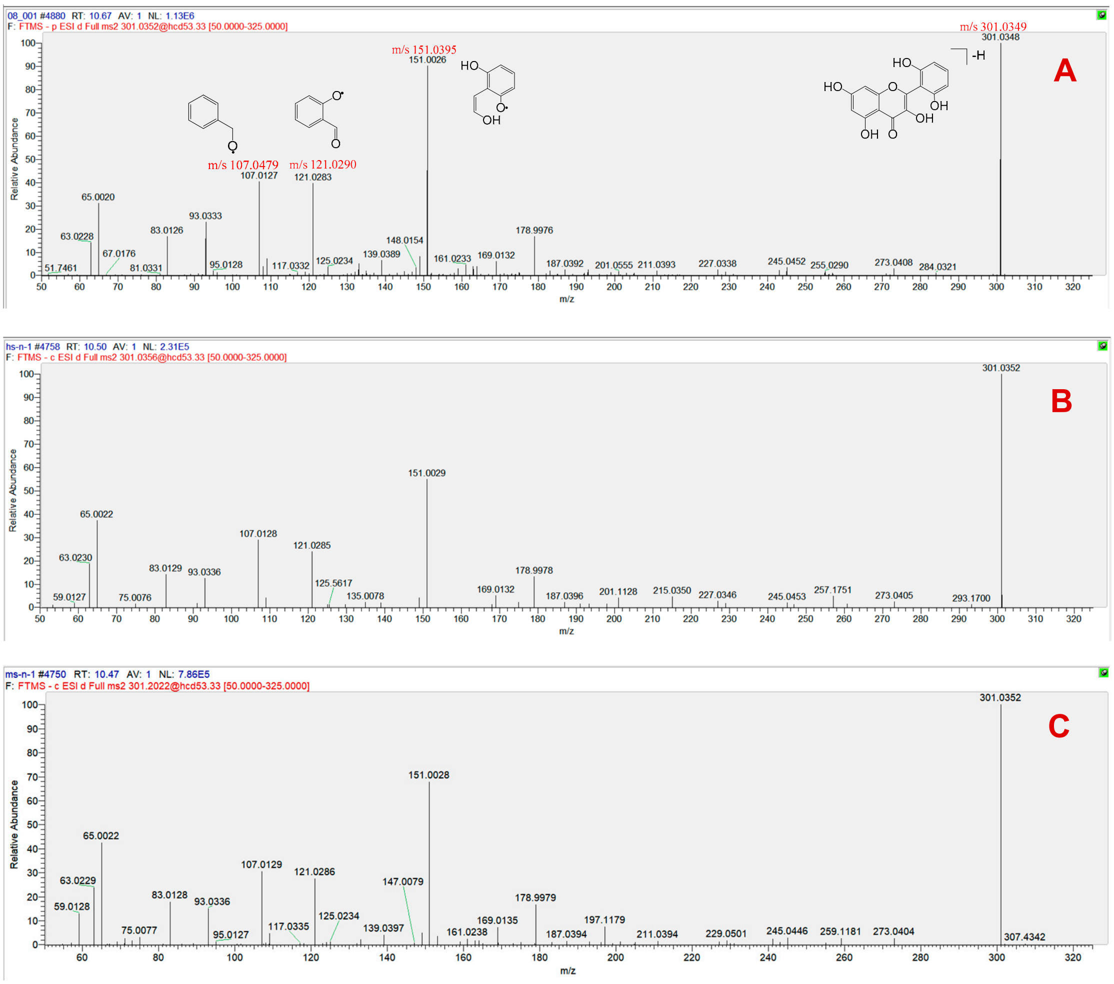


Fig. S1.46.1 The main results of viscidulin I (CAS 92519-95-4, $C_{15}H_{10}O_7$) and its corresponding peak in the TIC diagram using UHPLC-Q-Orbitrap-MS/MS analysis. (**A**) The MS/MS fragments of authentic standard viscidulin I. (**B**) The MS/MS spectra from chromatographic peak in the CoCA extract. (**C**) The MS/MS spectra from chromatographic peak in the CoNA extract.

**Note**: The m/z values in red are the calculated ones. The m/z calculation was based on the relative atomic masses of C (12.0000), H (1.007825), O (15.994915)[1]

**Identification**: As seen in Fig. S1.46.1, the extract ion peak, MS/MS spectra, and characteristic pears were highly similar. Thus, the chromatographic peaks in the extracts (CoCA, CoNA) were identified as viscidulin I (CAS 92519-95-4).

*Suppl. 1.47* Identification of luteolin (CAS 491-70-3, $C_{15}H_{10}O_6$, M.W. 286.24).

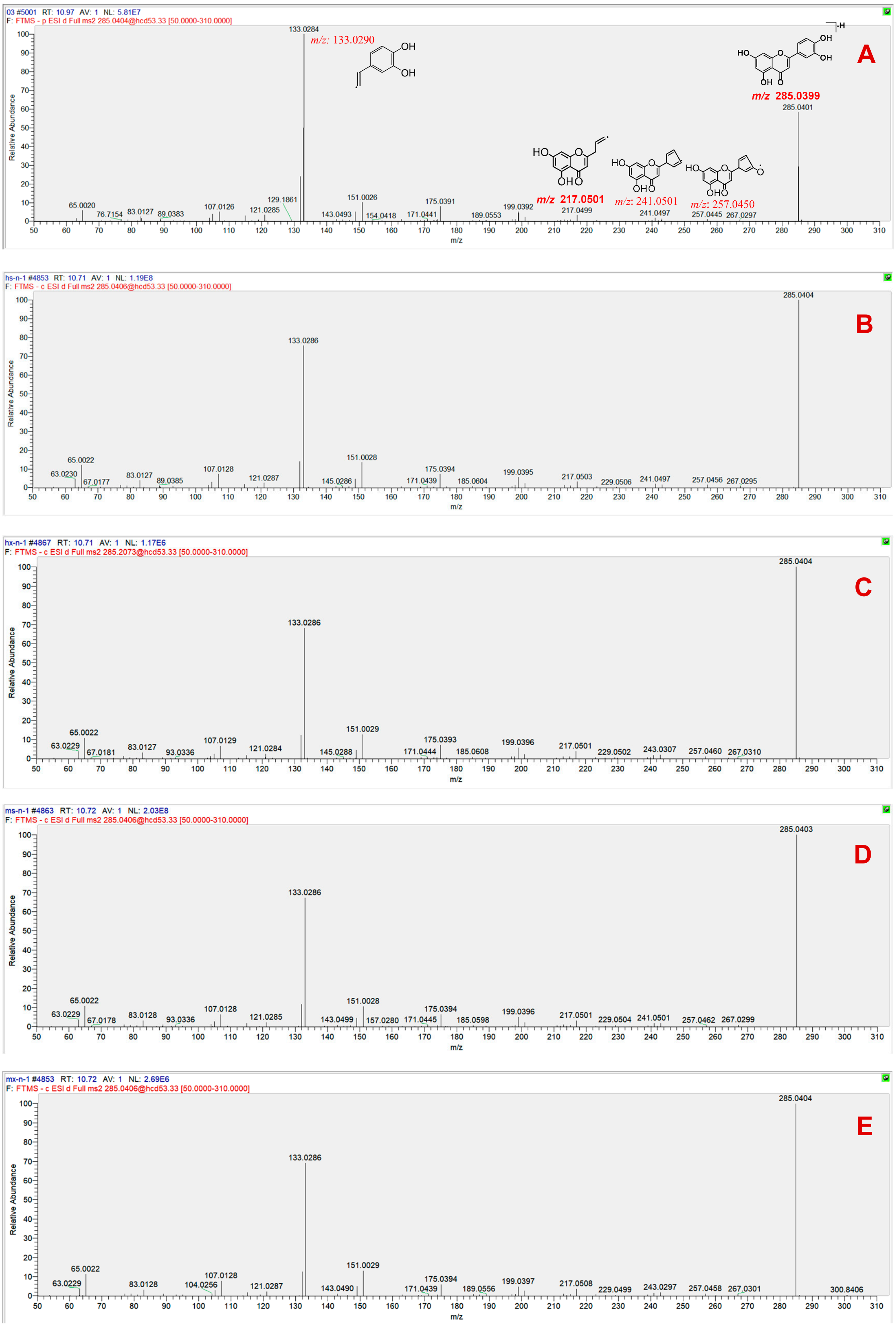


Fig. S1.47.1 The main results of luteolin (CAS 491-70-3, $C_{15}H_{10}O_6$) and its corresponding peak in the TIC diagram using UHPLC-Q-Orbitrap-MS/MS analysis. (**A**) The MS/MS fragments of authentic standard luteolin. (**B**) The MS/MS spectra from chromatographic peak in the CoCA extract. (**C**) The

MS/MS spectra from chromatographic peak in the CoCU extract. (**D**) The MS/MS spectra from chromatographic peak in the CoNA extract. (**E**) The MS/MS spectra from chromatographic peak in the CoNU extract.

**Note**: The m/z values in red are the calculated ones. The m/z calculation was based on the relative atomic masses of C (12.0000), H (1.007825), O (15.994915)[1]

**Identification**: As seen in Fig. S1.47.1, the extract ion peak, MS/MS spectra, and characteristic pears were highly similar. Thus, the chromatographic peaks in the extracts (CoCA, CoCU, CoNA, CoNU) were identified as luteolin (CAS 491-70-3).

*Suppl. 1.48* Identification of *S*-naringenin (CAS 480-41-1, $C_{15}H_{12}O_5$, M.W. 272.25).

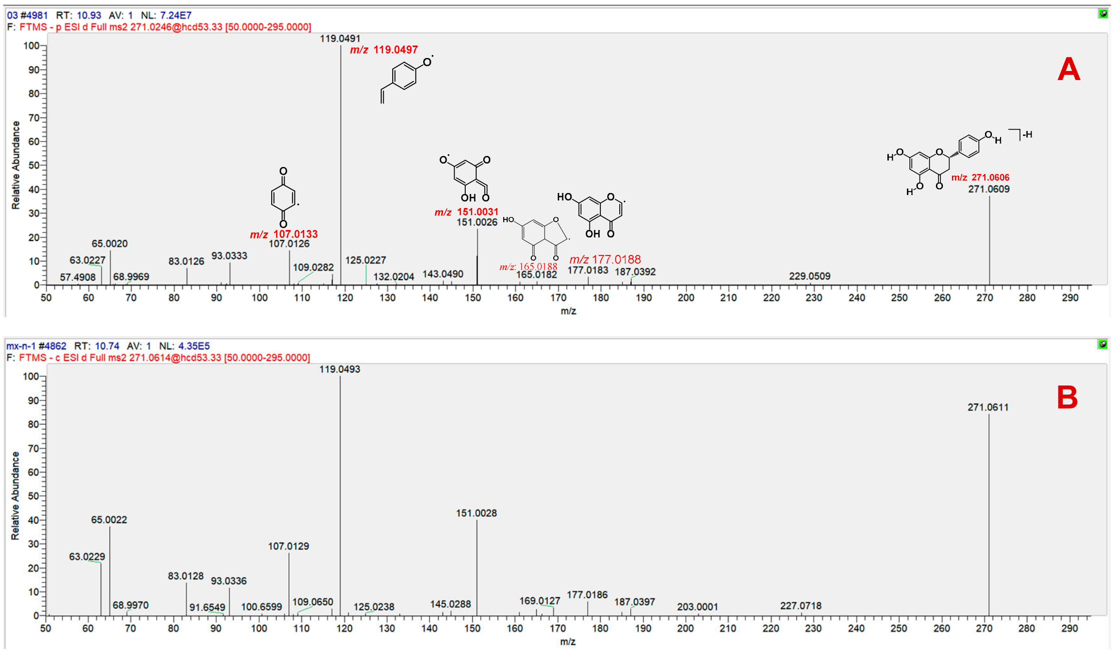


Fig. S1.48.1 The main results of *S*-naringenin (CAS 480-41-1, $C_{15}H_{12}O_5$) and its corresponding peak in the TIC diagram using UHPLC-Q-Orbitrap-MS/MS analysis. (**A**) The MS/MS fragments of authentic standard *S*-naringenin. (**B**) The MS/MS spectra from chromatographic peak in the CoNU extract.

**Note**: The m/z values in red are the calculated ones. The m/z calculation was based on the relative atomic masses of C (12.0000), H (1.007825), O (15.994915)[1]

**Identification**: As seen in Fig. S1.48.1, the extract ion peak, MS/MS spectra, and characteristic pears were highly similar. Thus, the chromatographic peaks in the extract (CoNU) were identified as S-naringenin (CAS 480-41-1).

*Suppl. 1.49* Identification of diosmetin (CAS 520-34-3, $C_{16}H_{12}O_6$, M.W. 300.267).

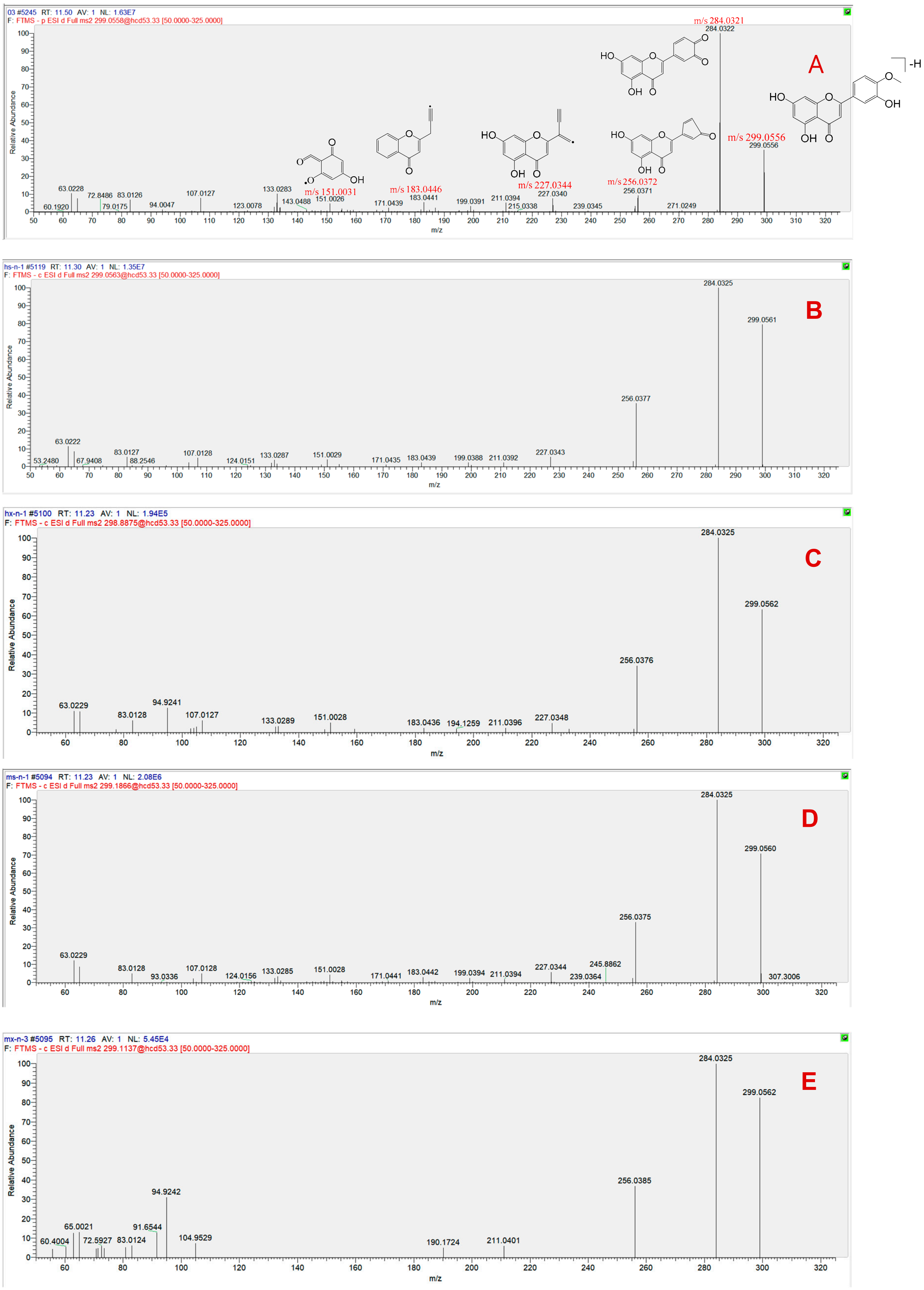

Fig. S1.49.1 The main results of diosmetin (CAS 520-34-3, $C_{16}H_{12}O_6$) and its corresponding peak in the TIC diagram using UHPLC-Q-Orbitrap-MS/MS analysis. (**A**) The MS/MS fragments of authentic

standard diosmetin. (**B**) The MS/MS spectra from chromatographic peak in the CoCA extract. (**C**) The MS/MS spectra from chromatographic peak in the CoCU extract. (**D**) The MS/MS spectra from chromatographic peak in the CoNA extract. (**E**) The MS/MS spectra from chromatographic peak in the CoNU extract.

**Note**: The m/z values in red are the calculated ones. The m/z calculation was based on the relative atomic masses of C (12.0000), H (1.007825), O (15.994915)[1]

**Identification**: As seen in Fig. S1.49.1, the extract ion peak, MS/MS spectra, and characteristic pears were highly similar. Thus, the chromatographic peaks in the extracts (CoCA, CoCU, CoNA, CoNU) were identified as diosmetin (CAS 520-34-3).

*Suppl. 1.50* Identification of apigenin (CAS 520-36-5, $C_{15}H_{10}O_5$, M.W. 270.24) .

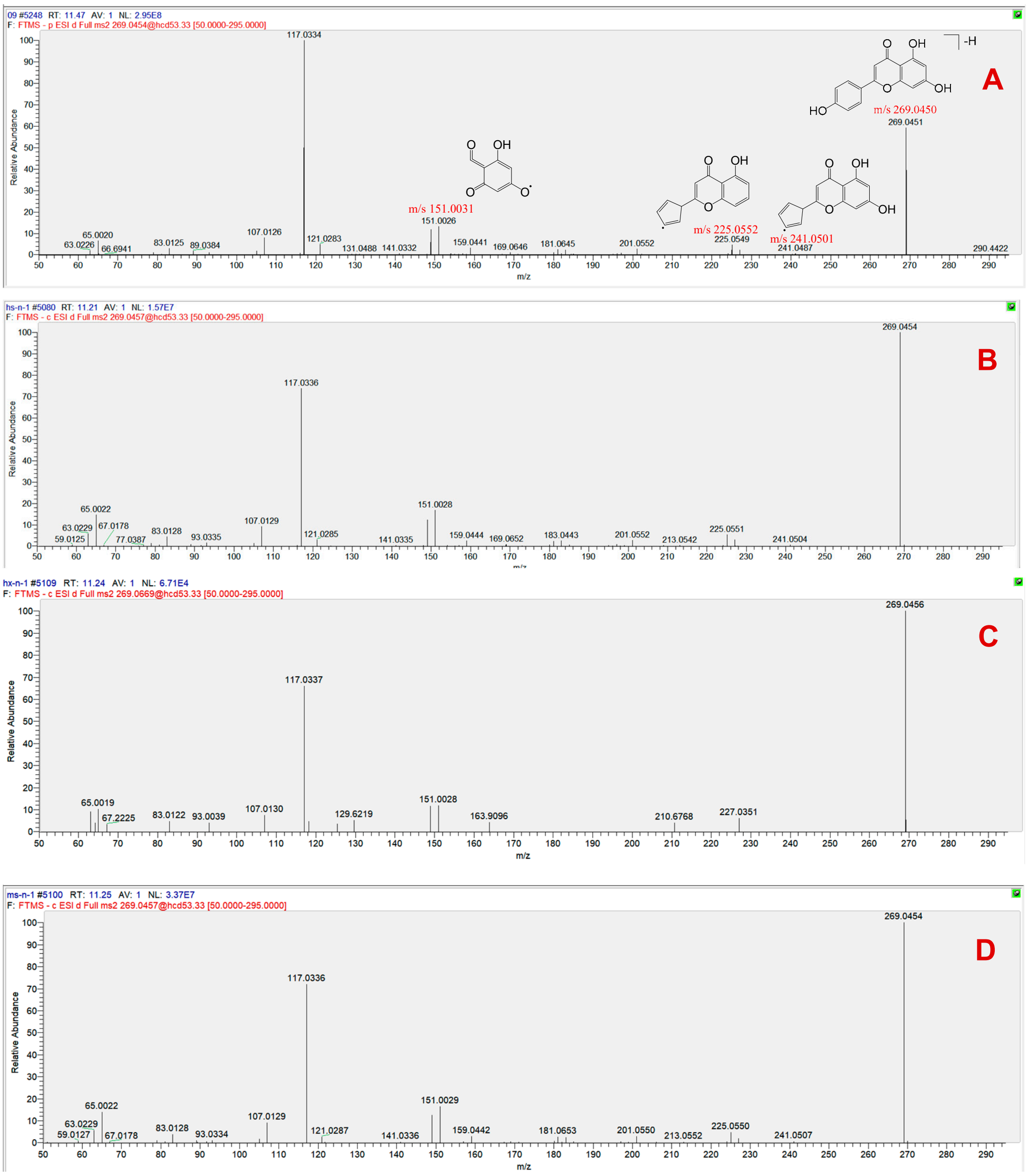


Fig. S1.50.1 The main results of apigenin (CAS 520-36-5, $C_{15}H_{10}O_5$) and its corresponding peak in the TIC diagram using UHPLC-Q-Orbitrap-MS/MS analysis. (**A**) The MS/MS fragments of authentic standard apigenin. (**B**) The MS/MS spectra from chromatographic peak in the CoCA extract. (**C**) The MS/MS spectra from chromatographic peak in the CoCU extract. (**D**) The MS/MS spectra from chromatographic peak in the CoNA extract.

**Note**: The m/z values in red are the calculated ones. The m/z calculation was based on the relative atomic masses of C (12.0000), H (1.007825), O (15.994915)[1]

**Identification**: As seen in Fig. S1.50.1, the extract ion peak, MS/MS spectra, and characteristic pears were highly similar. Thus, the chromatographic peaks in the extracts (CoCA, CoCU, CoNA) were identified as apigenin (CAS 520-36-5).

*Suppl. 1.51* Identification of chrysoeriol (CAS 491-71-4, $C_{16}H_{12}O_6$, M.W. 300.263).

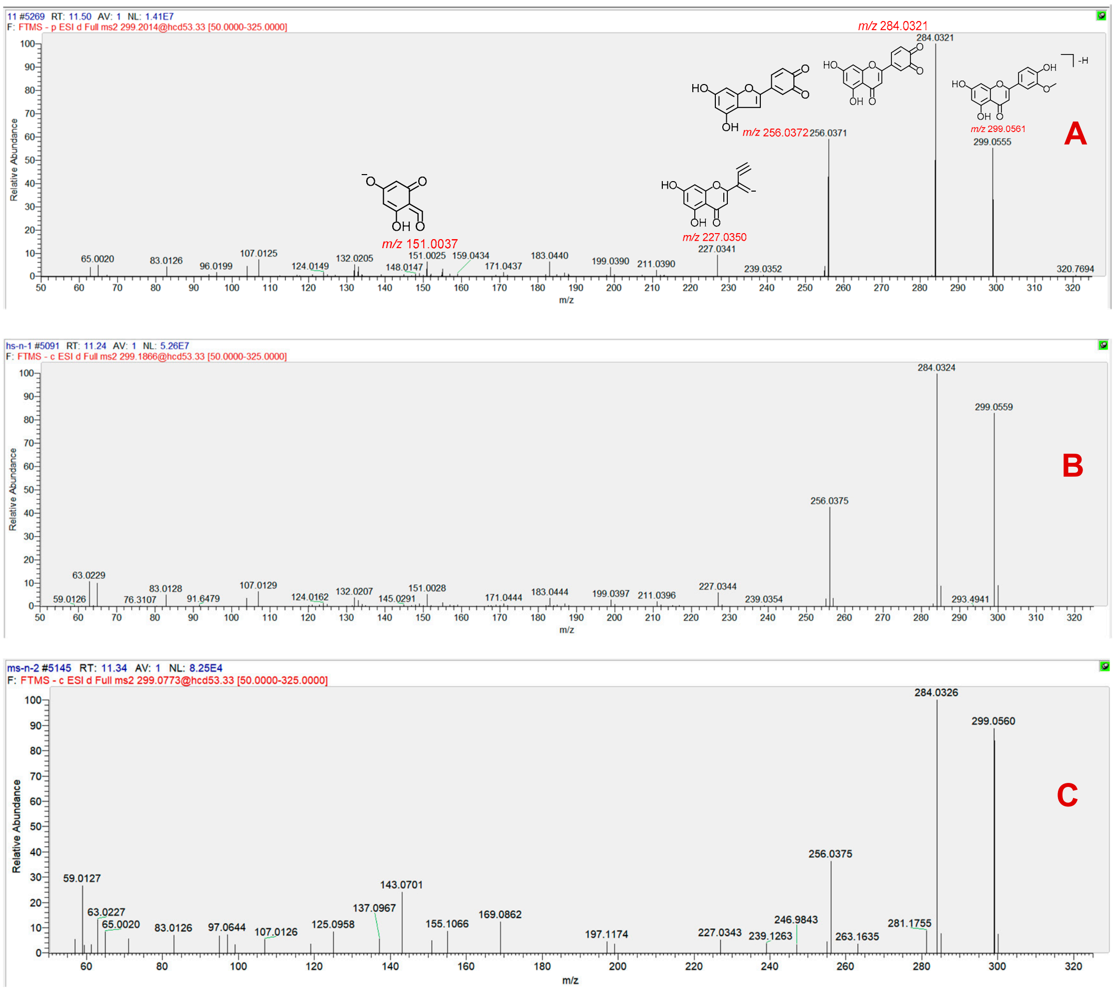


Fig. S1.51.1 The main results of chrysoeriol (CAS 491-71-4, $C_{16}H_{12}O_6$) and its corresponding peak in the TIC diagram using UHPLC-Q-Orbitrap-MS/MS analysis. (**A**) The MS/MS fragments of authentic standard chrysoeriol. (**B**) The MS/MS spectra from chromatographic peak in the CoCA extract. (**C**) The MS/MS spectra from chromatographic peak in the CoNA extract.

**Note**: The m/z values in red are the calculated ones. The m/z calculation was based on the relative atomic masses of C (12.0000), H (1.007825), O (15.994915)[1]

**Identification**: As seen in Fig. S1.51.1, the extract ion peak, MS/MS spectra, and characteristic pears were highly similar. Thus, the chromatographic peaks in the extracts (CoCA, CoNA) were identified as chrysoeriol (CAS 491-71-4).

*Suppl. 1.52* Identification of imperatorin (CAS 482-44-0, $C_{16}H_{14}O_4$, M.W. 270.284).

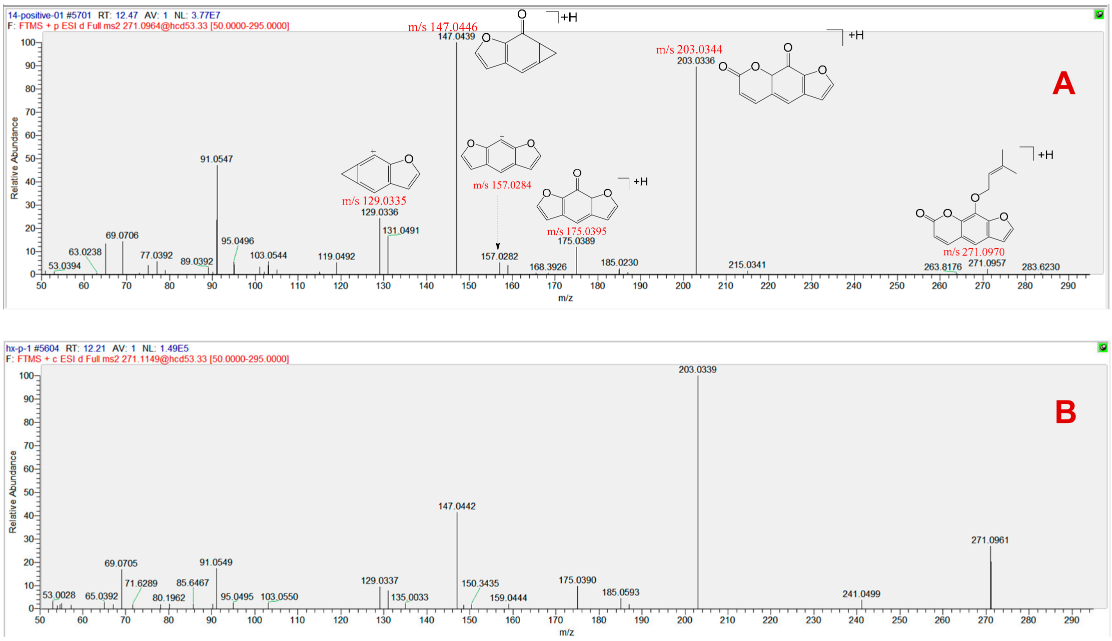


Fig. S1.52.1 The main results of imperatorin (CAS 482-44-0, $C_{16}H_{14}O_4$) and its corresponding peak in the TIC diagram using UHPLC-Q-Orbitrap-MS/MS analysis. (**A**) The MS/MS fragments of authentic standard imperatorin. (**B**) The MS/MS spectra from chromatographic peak in the CoCU extract.

**Note**: The m/z values in red are the calculated ones. The m/z calculation was based on the relative atomic masses of C (12.0000), H (1.007825), O (15.994915)[1]

**Identification**: As seen in Fig. S1.52.1, the extract ion peak, MS/MS spectra, and characteristic pears were highly similar. Thus, the chromatographic peaks in the extract (CoCU) were identified as imperatorin (CAS 482-44-0).

*Suppl. 1.53* Identification of chrysin (CAS 480-40-0, $C_{15}H_{10}O_4$, M.W. 254.24 ).

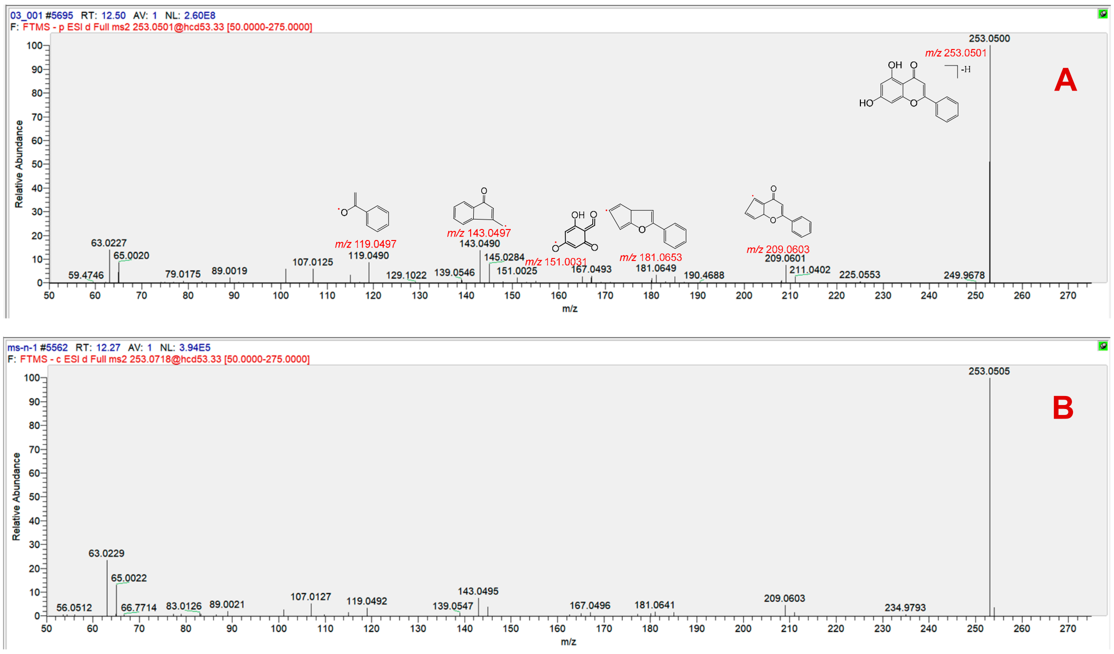


Fig. S1.53.1 The main results of chrysin (CAS 480-40-0, $C_{15}H_{10}O_4$) and its corresponding peak in the TIC diagram using UHPLC-Q-Orbitrap-MS/MS analysis. (**A**) The MS/MS fragments of authentic standard chrysin. (**B**) The MS/MS spectra from chromatographic peak in the CoNA extract.

**Note**: The m/z values in red are the calculated ones. The m/z calculation was based on the relative atomic masses of C (12.0000), H (1.007825), O (15.994915)[1]

**Identification**: As seen in Fig. S1.53.1, the extract ion peak, MS/MS spectra, and characteristic pears were highly similar. Thus, the chromatographic peaks in the extract (CoNA) were identified as chrysin (CAS 480-40-0).

*Suppl. 1.54* Identification of atractylenolide III (CAS 73030-71-4, $C_{15}H_{20}O_3$, M.W. 248.32).

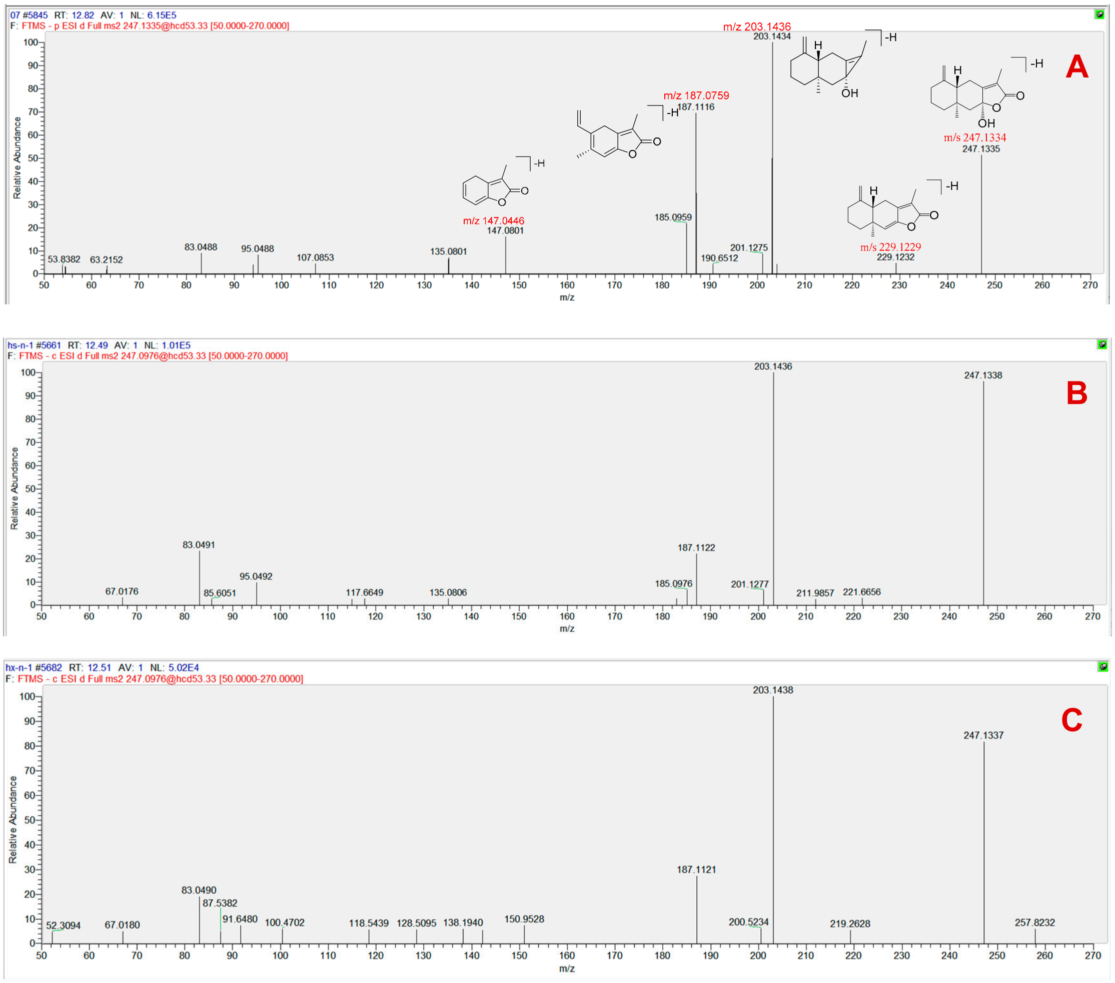


Fig. S1.54.1 The main results of atractylenolide III (CAS 73030-71-4, $C_{15}H_{20}O_3$) and its corresponding peak in the TIC diagram using UHPLC-Q-Orbitrap-MS/MS analysis. (**A**) The MS/MS fragments of authentic standard atractylenolide III. (**B**) The MS/MS spectra from chromatographic peak in the CoCA extract. (**C**) The MS/MS spectra from chromatographic peak in the CoCU extract.

**Note**: The m/z values in red are the calculated ones. The m/z calculation was based on the relative atomic masses of C (12.0000), H (1.007825), O (15.994915)[1]

**Identification**: As seen in Fig. S1.54.1, the extract ion peak, MS/MS spectra, and characteristic pears were highly similar. Thus, the chromatographic peaks in the extracts (CoCA, CoCU) were identified as atractylenolide III (CAS 73030-71-4).

*Suppl. 1.55* Identification of 6-gingerol (CAS 23513-14-6, $C_{17}H_{26}O_4$, M.W. 294.39).

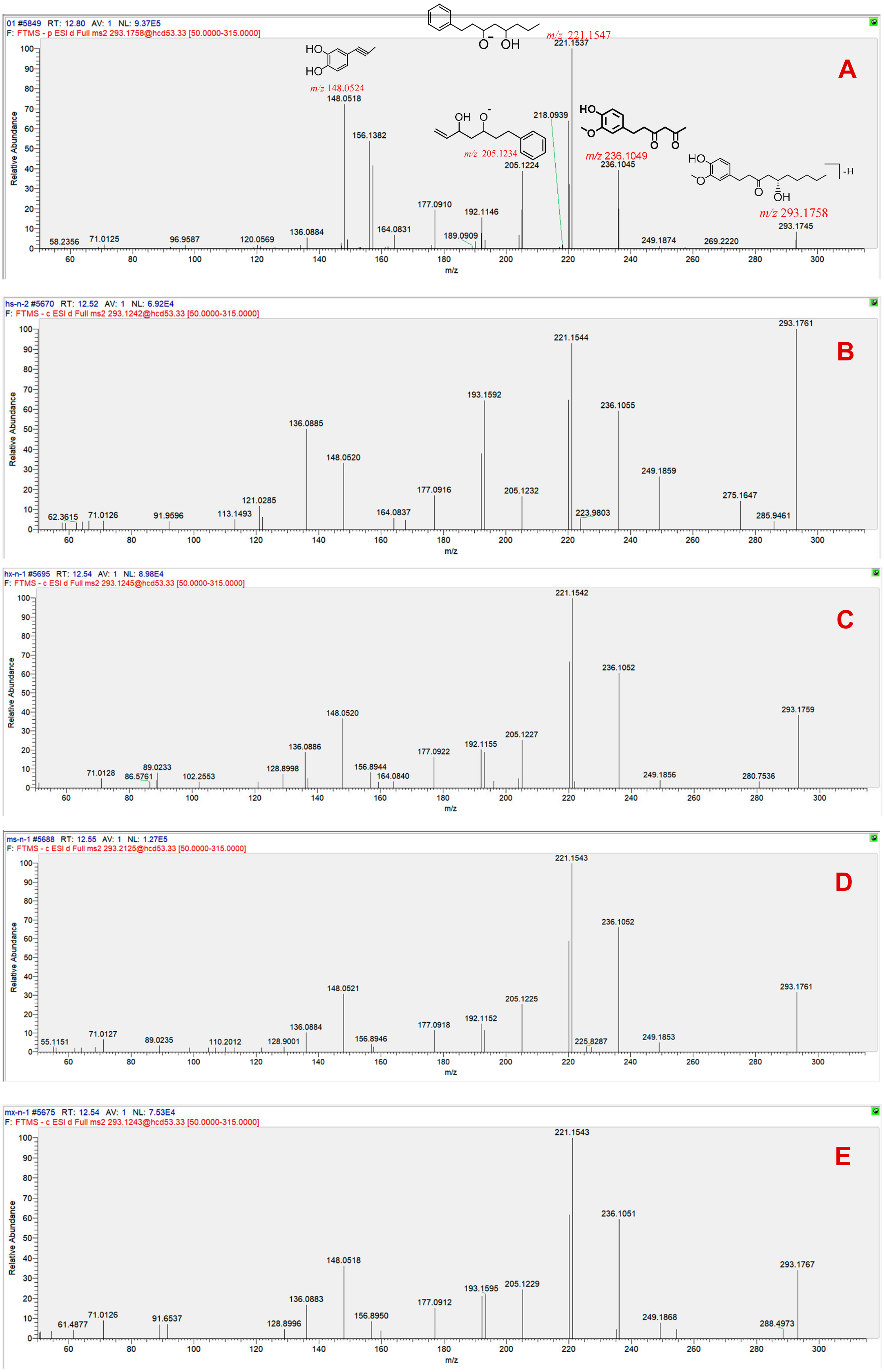


Fig. S1.55.1 The main results of 6-gingerol (CAS 23513-14-6, $C_{17}H_{26}O_4$) and its corresponding peak in

the TIC diagram using UHPLC-Q-Orbitrap-MS/MS analysis. (**A**) The MS/MS fragments of authentic standard 6-gingerol. (**B**) The MS/MS spectra from chromatographic peak in the CoCA extract. (**C**) The MS/MS spectra from chromatographic peak in the CoCU extract. (**D**) The MS/MS spectra from chromatographic peak in the CoNA extract. (**E**) The MS/MS spectra from chromatographic peak in the CoNU extract.

**Note**: The m/z values in red are the calculated ones. The m/z calculation was based on the relative atomic masses of C (12.0000), H (1.007825), O (15.994915)[1]

**Identification**: As seen in Fig. S1.55.1, the extract ion peak, MS/MS spectra, and characteristic pears were highly similar. Thus, the chromatographic peaks in the extracts (CoCA, CoCU, CoNA, CoNU) were identified as 6-gingerol (CAS 23513-14-6).

*Suppl. 1.56* Identification of costunolide (CAS 553-21-9, $C_{15}H_{20}O_2$, M.W. 232.32 ).

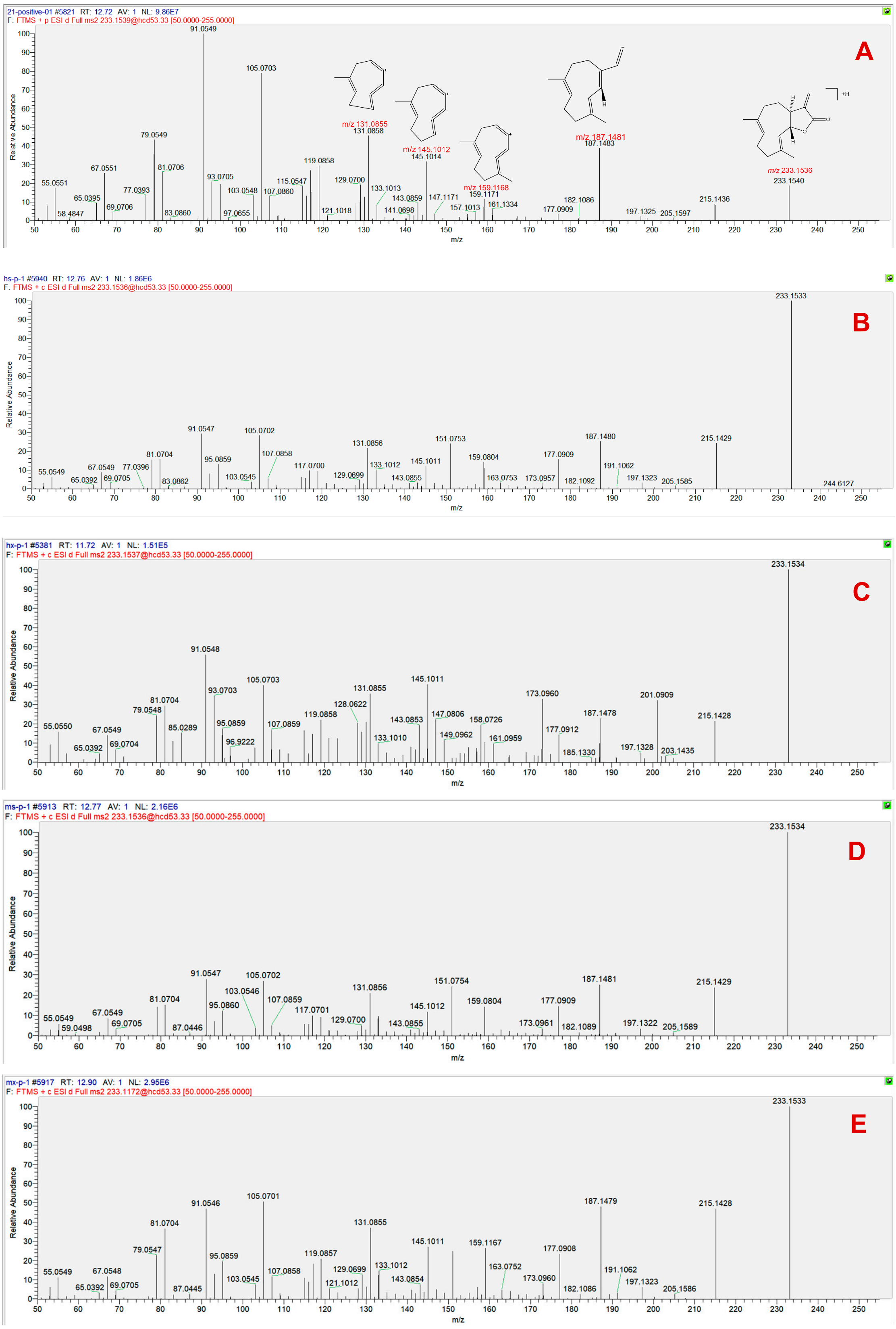


Fig. S1.56.1 The main results of costunolide (CAS 553-21-9, $C_{15}H_{20}O_2$) and its corresponding peak in the TIC diagram using UHPLC-Q-Orbitrap-MS/MS analysis. (**A**) The MS/MS fragments of authentic standard costunolide. (**B**) The MS/MS spectra from chromatographic peak in the CoCA extract. (**C**) The MS/MS spectra from chromatographic peak in the CoCU extract. (**D**) The MS/MS spectra from

chromatographic peak in the CoNA extract. (**E**) The MS/MS spectra from chromatographic peak in the CoNU extract.

**Note**: The m/z values in red are the calculated ones. The m/z calculation was based on the relative atomic masses of C (12.0000), H (1.007825), O (15.994915)[1]

**Identification**: As seen in Fig. S1.56.1, the extract ion peak, MS/MS spectra, and characteristic pears were highly similar. Thus, the chromatographic peaks in the extracts (CoCA, CoCU, CoNA, CoNU) were identified as costunolide (CAS 553-21-9).

*Suppl. 1.57* Identification of alantolactone (CAS 546-43-0, $C_{15}H_{20}O_2$, M.W. 232.32).

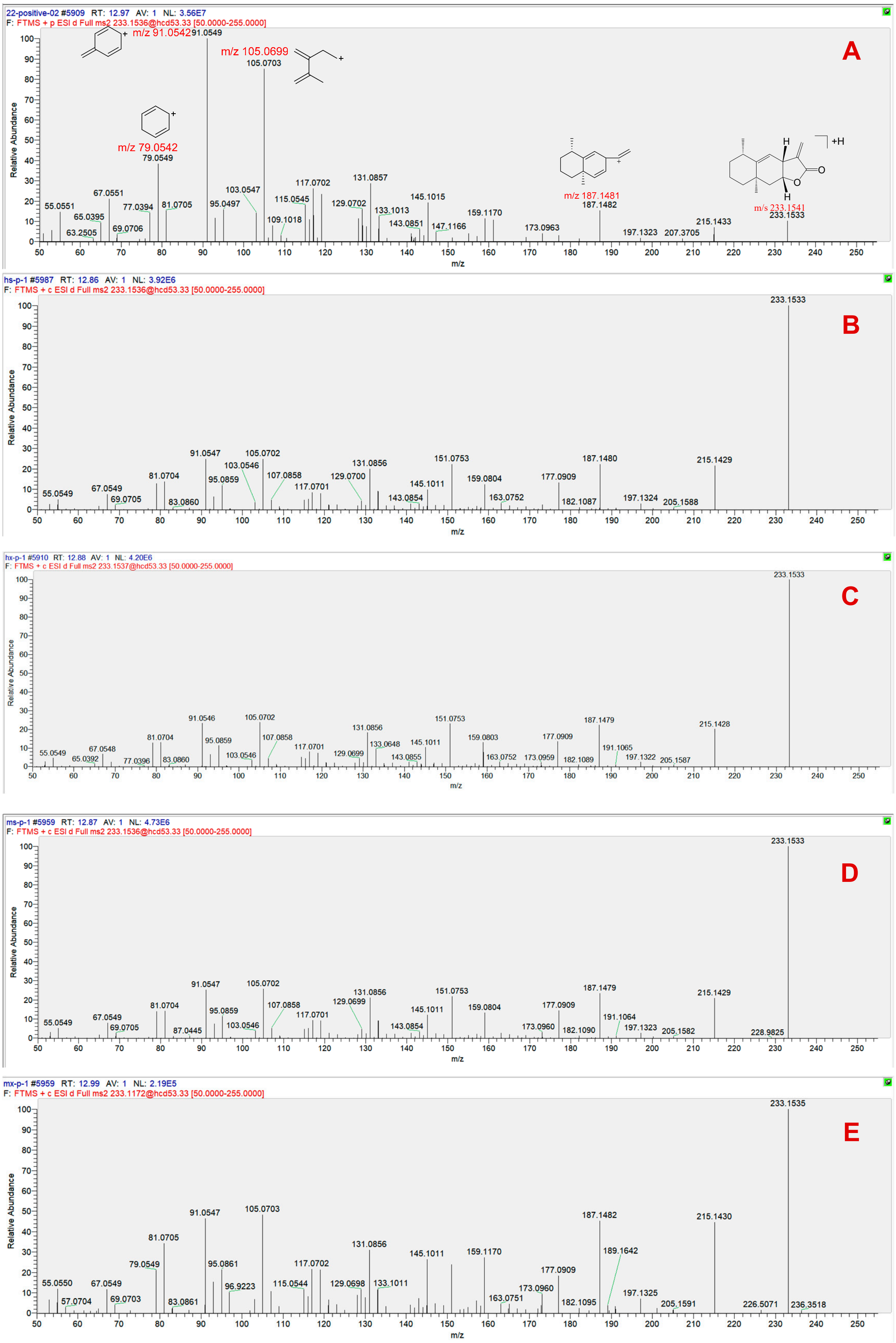


Fig. S1.57.1 The main results of alantolactone (CAS 546-43-0, $C_{15}H_{20}O_2$) and its corresponding peak in the TIC diagram using UHPLC-Q-Orbitrap-MS/MS analysis. (**A**) The MS/MS fragments of authentic standard alantolactone. (**B**) The MS/MS spectra from chromatographic peak in the CoCA extract. (**C**) The MS/MS spectra from chromatographic peak in the CoCU extract. (**D**) The MS/MS spectra from

chromatographic peak in the CoNA extract. (**E**) The MS/MS spectra from chromatographic peak in the CoNU extract.

**Note**: The m/z values in red are the calculated ones. The m/z calculation was based on the relative atomic masses of C (12.0000), H (1.007825), O (15.994915)[1]

**Identification**: As seen in Fig. S1.57.1, the extract ion peak, MS/MS spectra, and characteristic pears were highly similar. Thus, the chromatographic peaks in the extracts (CoCA, CoCU, CoNA, CoNU) were identified as alantolactone (CAS 546-43-0).

*Suppl. 1.58* Identification of zerumbone (CAS 471-05-6, $C_{15}H_{22}O$, M.W. 218.33).

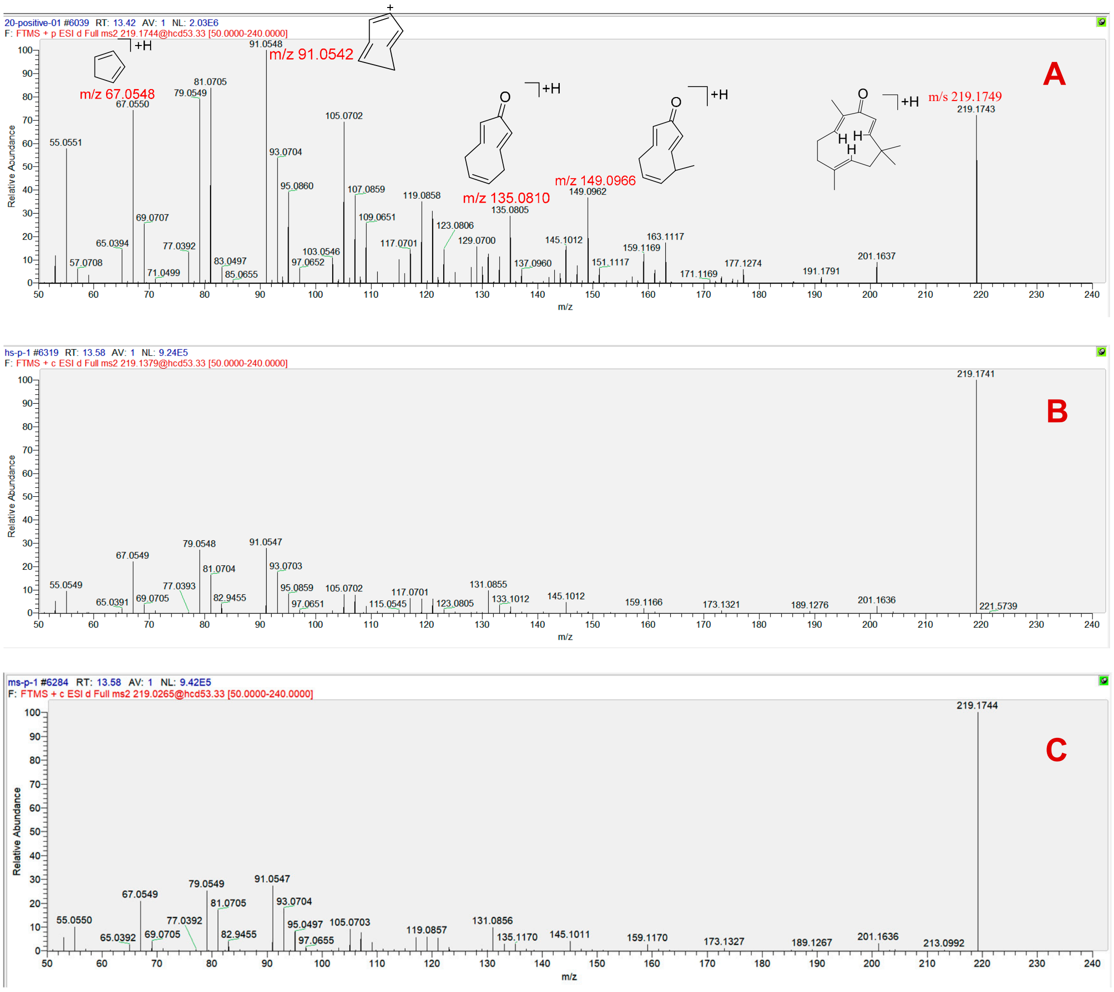


Fig. S1.58.1 The main results of zerumbone (CAS 471-05-6, $C_{15}H_{22}O$) and its corresponding peak in the TIC diagram using UHPLC-Q-Orbitrap-MS/MS analysis. (**A**) The MS/MS fragments of authentic standard zerumbone. (**B**) The MS/MS spectra from chromatographic peak in the CoCA extract. (**C**) The MS/MS spectra from chromatographic peak in the CoNA extract.

**Note**: The m/z values in red are the calculated ones. The m/z calculation was based on the relative atomic masses of C (12.0000), H (1.007825), O (15.994915)[1]

**Identification**: As seen in Fig. S1.58.1, the extract ion peak, MS/MS spectra, and characteristic pears were highly similar. Thus, the chromatographic peaks in the extracts (CoCA, CoNA) were identified as zerumbone (CAS 471-05-6).

*Suppl. 1.59* Identification of emodin (CAS 518-82-1, $C_{15}H_{10}O_5$, M.W. 270.24).

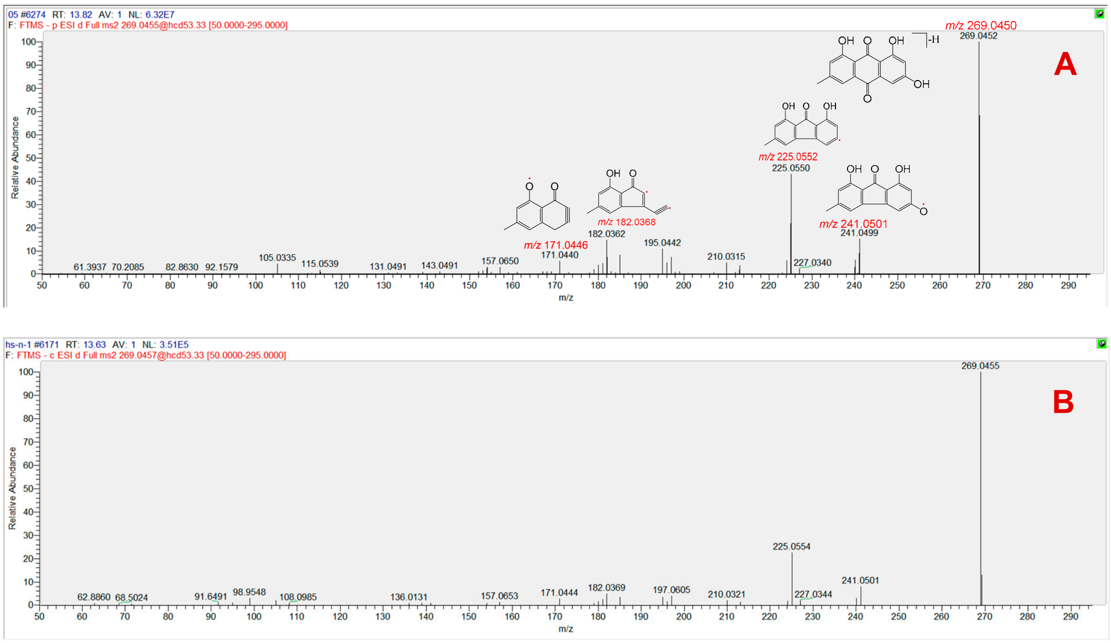


Fig. S1.59.1 The main results of emodin (CAS 518-82-1, $C_{15}H_{10}O_5$) and its corresponding peak in the TIC diagram using UHPLC-Q-Orbitrap-MS/MS analysis. (**A**) The MS/MS fragments of authentic standard emodin. (**B**) The MS/MS spectra from chromatographic peak in the CoCA extract.

**Note**: The m/z values in red are the calculated ones. The m/z calculation was based on the relative atomic masses of C (12.0000), H (1.007825), O (15.994915)[1]

**Identification**: As seen in Fig. S1.59.1, the extract ion peak, MS/MS spectra, and characteristic pears were highly similar. Thus, the chromatographic peaks in the extract (CoCA) were identified as emodin (CAS 518-82-1).

*Suppl. 1.60* Identification of agrimol B (CAS 55576-66-4, $C_{37}H_{46}O_{12}$, M.W. 696.31).

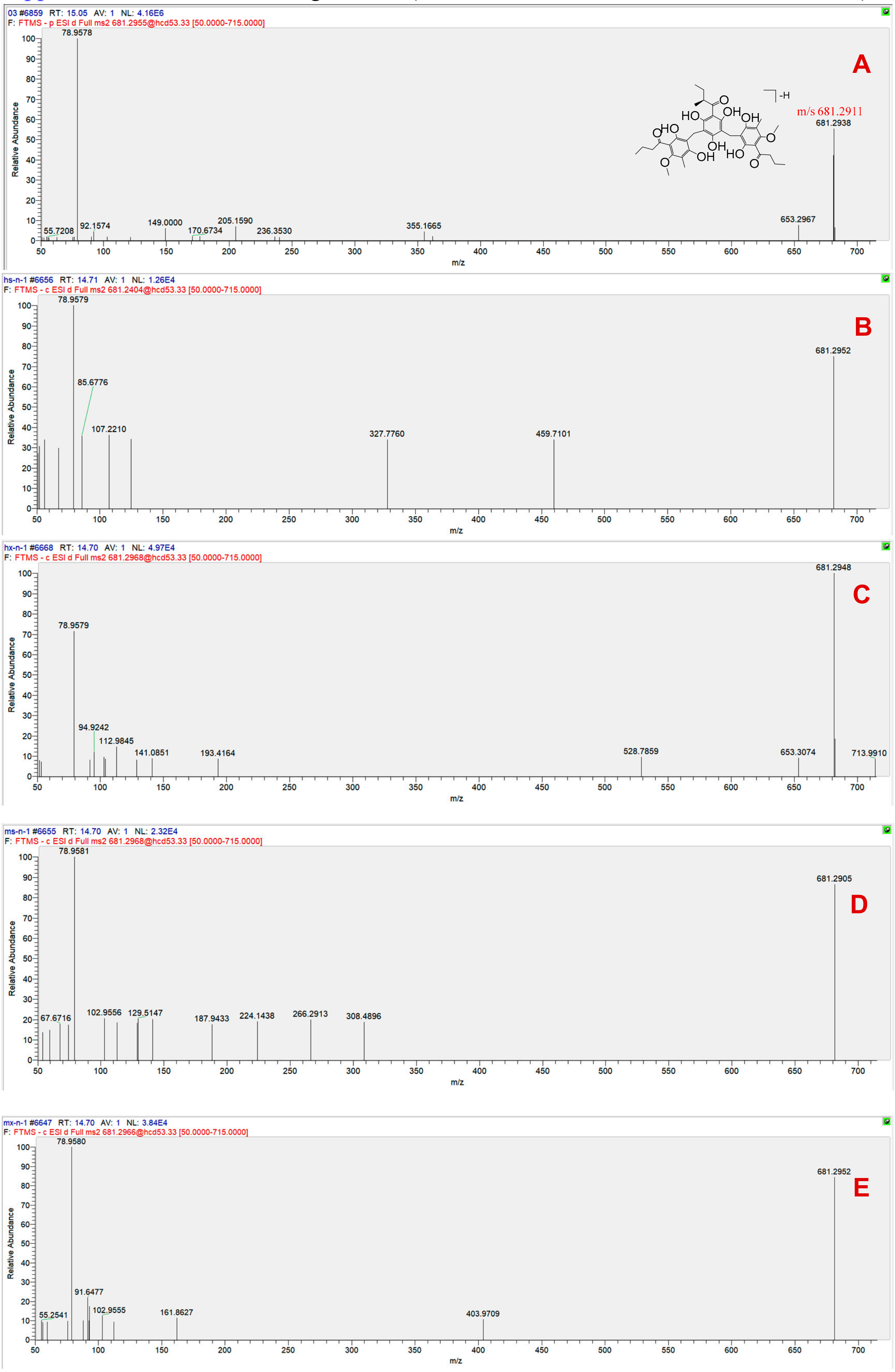


Fig. S1.60.1 The main results of agrimol B (CAS 55576-66-4, $C_{37}H_{46}O_{12}$) and its corresponding peak in the TIC diagram using UHPLC-Q-Orbitrap-MS/MS analysis. (**A**) The MS/MS fragments of authentic

standard agrimol B. (**B**) The MS/MS spectra from chromatographic peak in the CoCA extract. (**C**) The MS/MS spectra from chromatographic peak in the CoCU extract. (**D**) The MS/MS spectra from chromatographic peak in the CoNA extract. (**E**) The MS/MS spectra from chromatographic peak in the CoNU extract.

**Note**: The m/z values in red are the calculated ones. The m/z calculation was based on the relative atomic masses of C (12.0000), H (1.007825), O (15.994915)[1]

**Identification**: As seen in Fig. S1.60.1, the extract ion peak, MS/MS spectra, and characteristic pears were highly similar. Thus, the chromatographic peaks in the extracts (CoCA, CoCU, CoNA, CoNU) were identified as agrimol B (CAS 55576-66-4).

*Suppl. 1.61* Identification of $\alpha$-linolenic acid (CAS 463-40-1, $C_{18}H_{30}O_2$, M.W.

278.43 ).

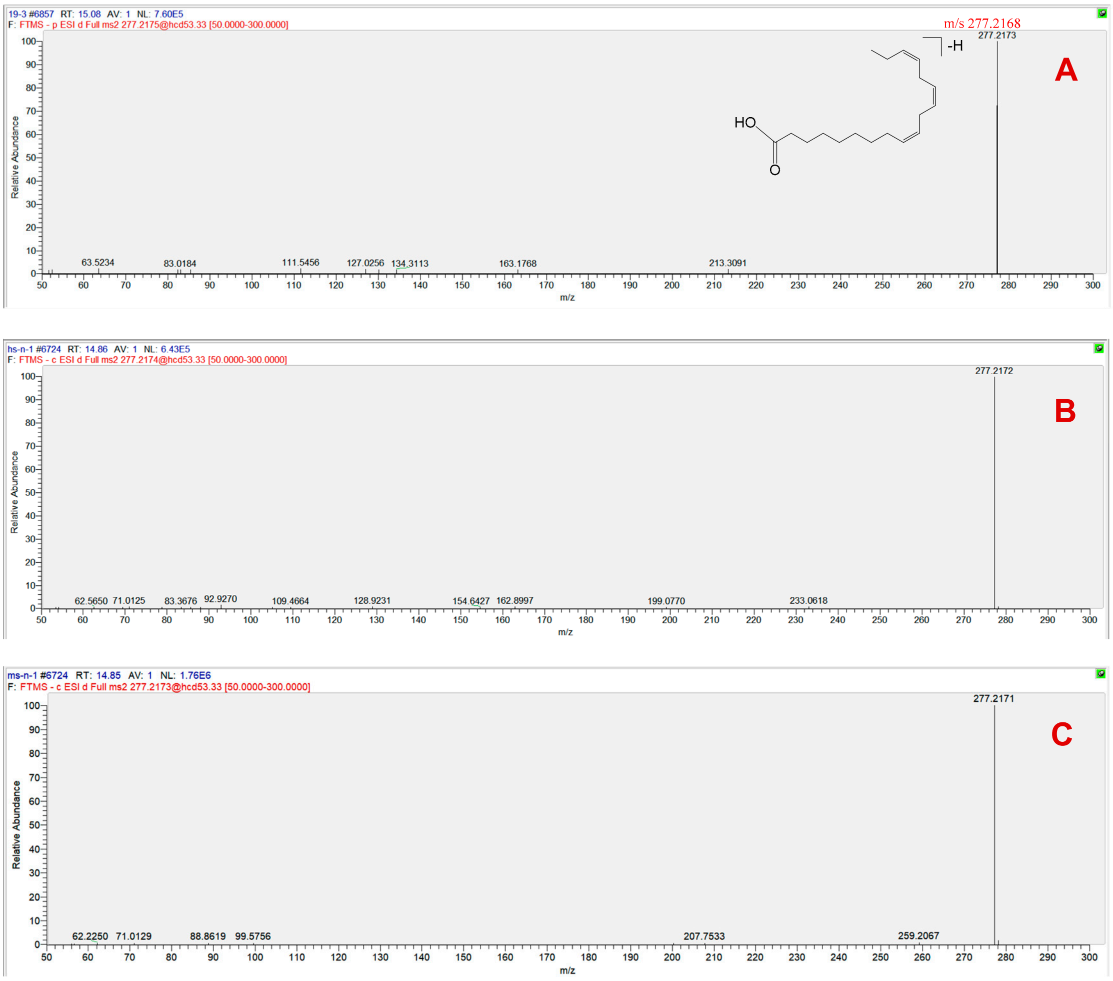


Fig. S1.61.1 The main results of α-linolenic acid (CAS 463-40-1, $C_{18}H_{30}O_2$) and its corresponding peak in the TIC diagram using UHPLC-Q-Orbitrap-MS/MS analysis. (**A**) The MS/MS fragments of authentic standard α-linolenic acid. (**B**) The MS/MS spectra from chromatographic peak in the CoCA extract. (**C**) The MS/MS spectra from chromatographic peak in the CoNA extract.

**Note**: The m/z values in red are the calculated ones. The m/z calculation was based on the relative atomic masses of C (12.0000), H (1.007825), O (15.994915)[1]

**Identification**: As seen in Fig. S1.61.1, the extract ion peak, MS/MS spectra, and characteristic pears were highly similar. Thus, the chromatographic peaks in the extracts (CoCA, CoNA) were identified as α-linolenic acid (CAS 463-40-1).

*Suppl. 1.62* Identification of linoleic acid (CAS 60-33-3, $C_{18}H_{32}O_2$, M.W. 280.45 ).

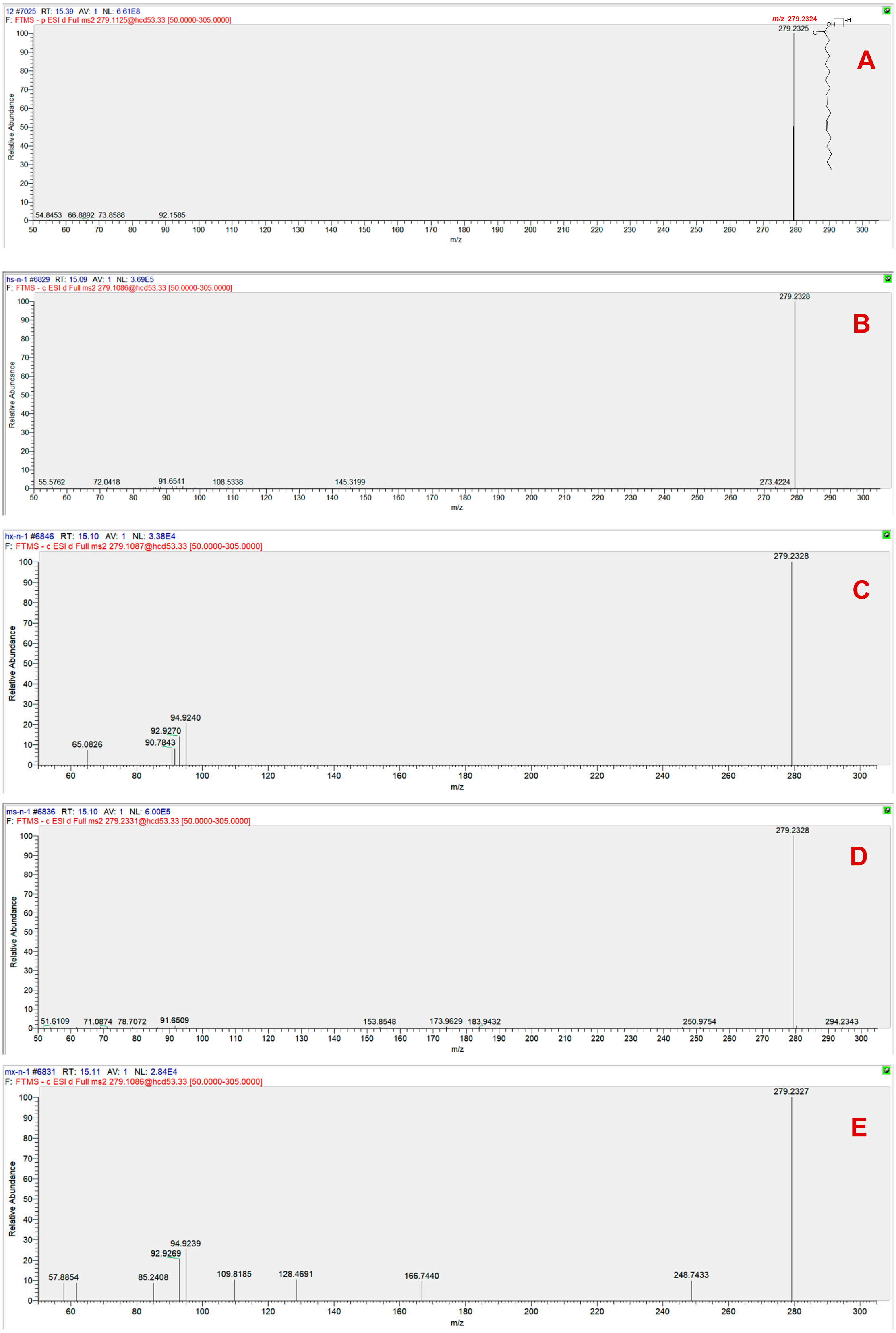


Fig. S1.62.1 The main results of linoleic acid (CAS 60-33-3, $C_{18}H_{32}O_2$) and its corresponding peak in the TIC diagram using UHPLC-Q-Orbitrap-MS/MS analysis. (**A**) The MS/MS fragments of authentic standard linoleic acid. (**B**) The MS/MS spectra from chromatographic peak in the CoCA extract. (**C**) The MS/MS spectra from chromatographic peak in the CoCU extract. (**D**) The MS/MS spectra from

chromatographic peak in the CoNA extract. (**E**) The MS/MS spectra from chromatographic peak in the CoNU extract.

**Note**: The m/z values in red are the calculated ones. The m/z calculation was based on the relative atomic masses of C (12.0000), H (1.007825), O (15.994915)[1]

**Identification**: As seen in Fig. S1.62.1, the extract ion peak, MS/MS spectra, and characteristic pears were highly similar. Thus, the chromatographic peaks in the extracts (CoCA, CoCU, CoNA, CoNU) were identified as linoleic acid (CAS 60-33-3).

*Suppl. 1.63* Identification of (9Z,12Z)-N-benzyloctadeca-9,12-dienamide (CAS 18286-71-0, $C_{25}H_{39}NO$, M.W. 369.6).

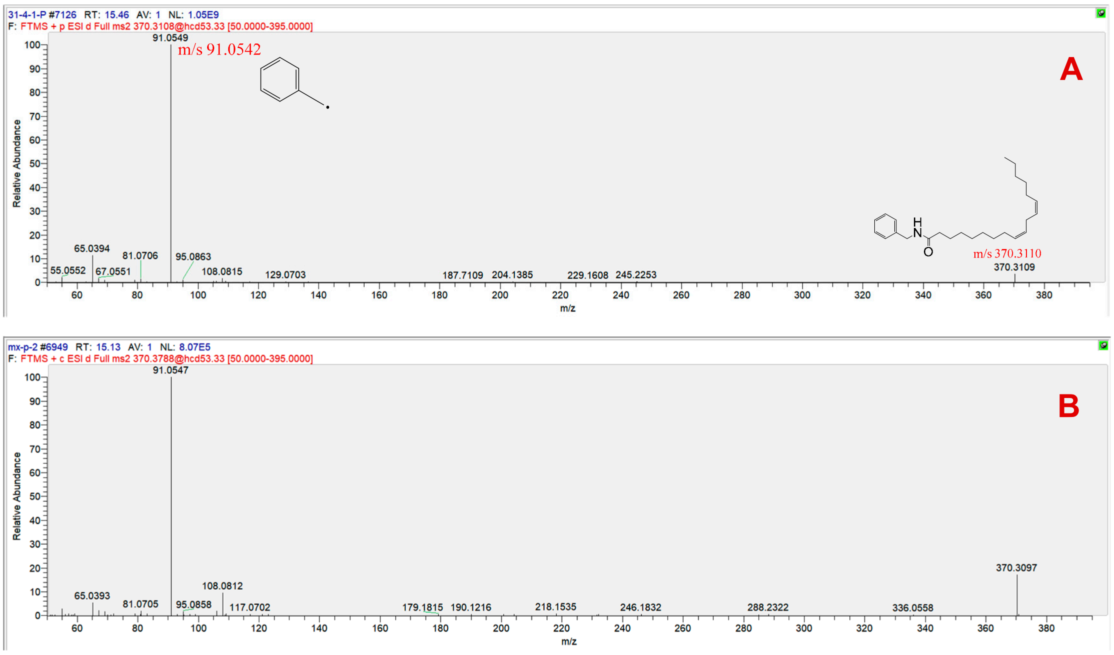


Fig. S1.63.1 The main results of (9Z,12Z)-N-benzyloctadeca-9,12-dienamide (CAS 18286-71-0, $C_{25}H_{39}NO$) and its corresponding peak in the TIC diagram using UHPLC-Q-Orbitrap-MS/MS analysis. (**A**) The MS/MS fragments of authentic standard (9Z,12Z)-N-benzyloctadeca-9,12-dienamide. (**B**) The MS/MS spectra from chromatographic peak in the CoNU extract.

**Note**: The m/z values in red are the calculated ones. The m/z calculation was based on the relative atomic masses of C (12.0000), H (1.007825), O (15.994915), and N (14.003074)[1]

**Identification**: As seen in Fig. S1.63.1, the extract ion peak, MS/MS spectra, and characteristic pears were highly similar. Thus, the chromatographic peaks in the extract (CoNU) were identified as (9Z,12Z)-N-benzyloctadeca-9,12-dienamide (CAS 18286-71-0).

*Suppl. 1.64* Identification of palmitic acid (CAS 57-10-3, $C_{16}H_{32}O_2$, M.W. 256.43).

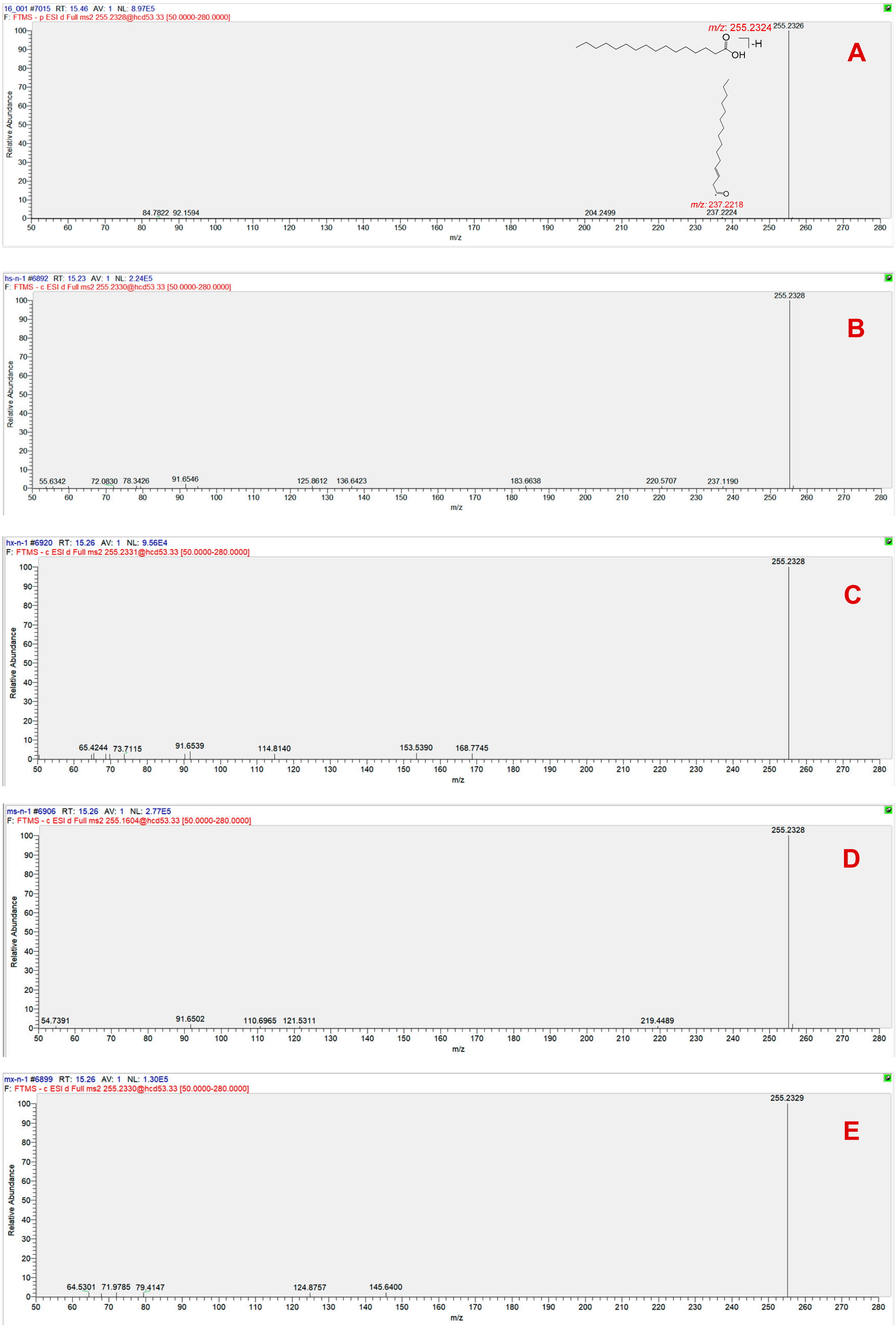


Fig. S1.64.1 The main results of palmitic acid (CAS 57-10-3, $C_{16}H_{32}O_2$) and its corresponding peak in the TIC diagram using UHPLC-Q-Orbitrap-MS/MS analysis. (**A**) The MS/MS fragments of authentic standard palmitic acid. (**B**) The MS/MS spectra from chromatographic peak in the CoCA extract. (**C**) The MS/MS spectra from chromatographic peak in the CoCU extract. (**D**) The MS/MS spectra from

chromatographic peak in the CoNA extract. (**E**) The MS/MS spectra from chromatographic peak in the CoNU extract.

**Note**: The m/z values in red are the calculated ones. The m/z calculation was based on the relative atomic masses of C (12.0000), H (1.007825), O (15.994915)[1]

**Identification**: As seen in Fig. S1.64.1, the extract ion peak, MS/MS spectra, and characteristic pears were highly similar. Thus, the chromatographic peaks in the extracts (CoCA, CoCU, CoNA, CoNU) were identified as palmitic acid (CAS 57-10-3).

*Suppl. 1.65* Identification of oleic acid (CAS 112-80-1, $C_{18}H_{34}O_2$, M.W. 282.46 ).

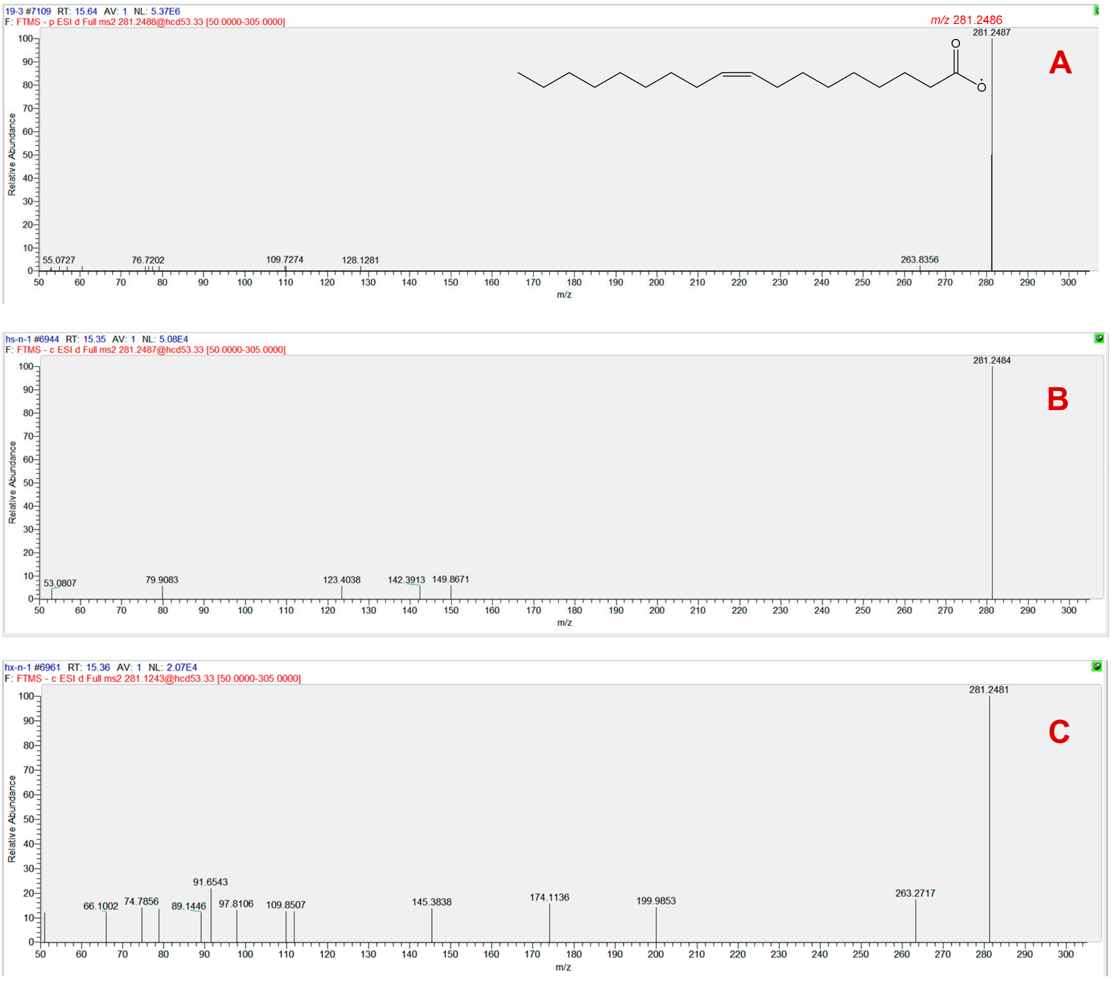


Fig. S1.65.1 The main results of oleic acid (CAS 112-80-1, $C_{18}H_{34}O_2$) and its corresponding peak in the TIC diagram using UHPLC-Q-Orbitrap-MS/MS analysis. (**A**) The MS/MS fragments of authentic standard oleic acid. (**B**) The MS/MS spectra from chromatographic peak in the CoCA extract. (**C**) The MS/MS spectra from chromatographic peak in the CoCU extract.

**Note**: The m/z values in red are the calculated ones. The m/z calculation was based on the relative atomic masses of C (12.0000), H (1.007825), O (15.994915)[1]

**Identification**: As seen in Fig. S1.65.1, the extract ion peak, MS/MS spectra, and characteristic pears were highly similar. Thus, the chromatographic peaks in the extracts (CoCA, CoCU) were identified as oleic acid (CAS 112-80-1).

*Suppl. 1.66* Identification of stearic acid (CAS 57-11-4, $C_{18}H_{36}O_2$, M.W. 284.484 ).

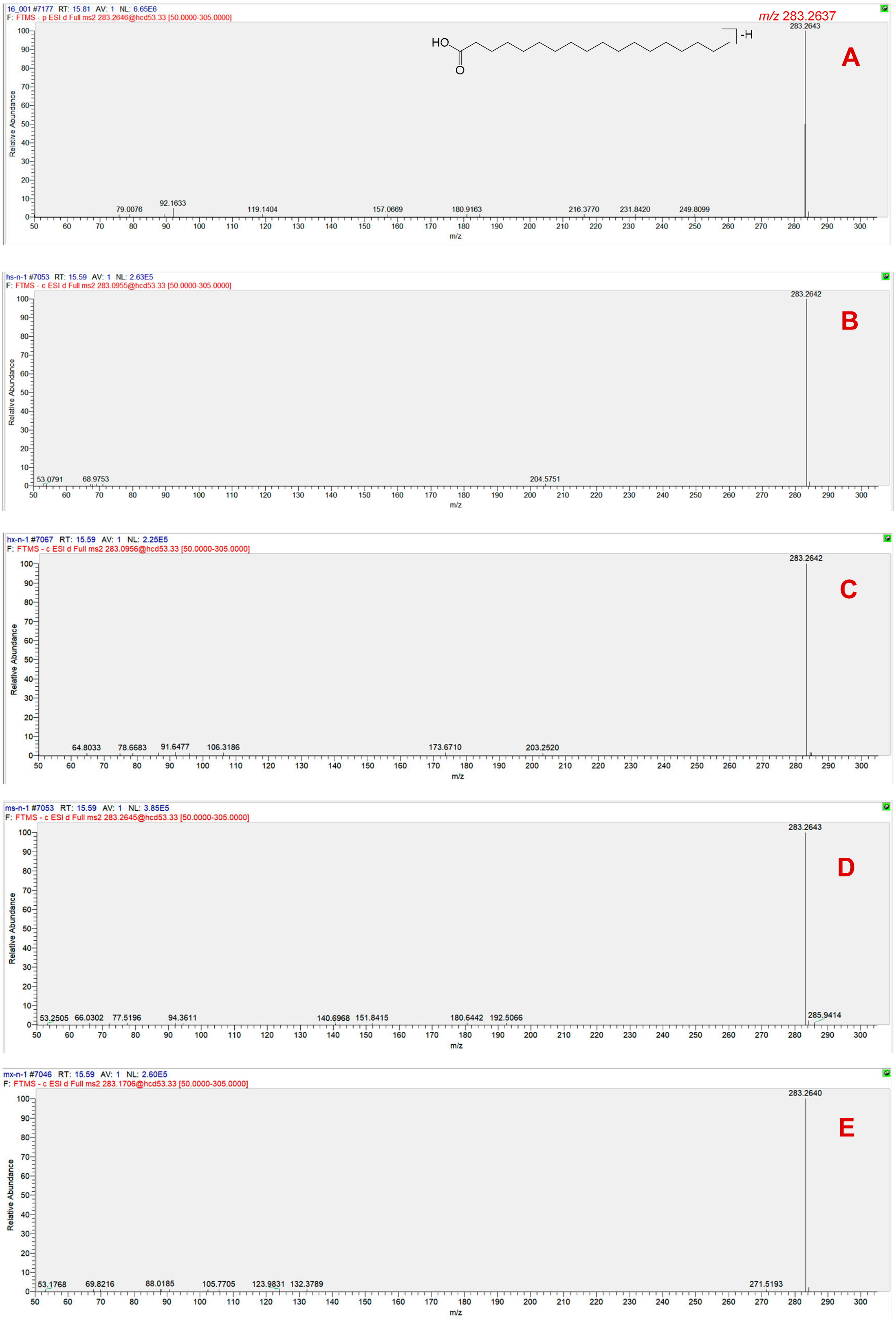


Fig. S1.66.1 The main results of stearic acid (CAS 57-11-4, $C_{18}H_{36}O_2$) and its corresponding peak in the TIC diagram using UHPLC-Q-Orbitrap-MS/MS analysis. (**A**) The MS/MS fragments of authentic standard stearic acid. (**B**) The MS/MS spectra from chromatographic peak in the CoCA extract. (**C**) The MS/MS spectra from chromatographic peak in the CoCU extract. (**D**) The MS/MS spectra from

chromatographic peak in the CoNA extract. (**E**) The MS/MS spectra from chromatographic peak in the CoNU extract.

**Note**: The m/z values in red are the calculated ones. The m/z calculation was based on the relative atomic masses of C (12.0000), H (1.007825), O (15.994915)[1]

**Identification**: As seen in Fig. S1.66.1, the extract ion peak, MS/MS spectra, and characteristic pears were highly similar. Thus, the chromatographic peaks in the extracts (CoCA, CoCU, CoNA, CoNU) were identified as stearic acid (CAS 57-11-4).

**References**:

[1] Jürgen H. Gross, *Mass spectrometry*. 2013, Beijing: Science press.
