# Supplementary material for: Simultaneous Quantification of 66 Compounds in Two Tibetan Codonopsis Species Reveals Four Chemical Features by Database-Enabled UHPLC-Q-Orbitrap-MS/MS Analysis
Source: Molecules. 2024 Nov 3;29(21):5203. doi: 10.3390/molecules29215203 (PMC11547486; doi:10.3390/molecules29215203)
Supplement: Supplementary file 1 [file molecules-29-05203-s001.zip › Supplementary data S2. The total ion chromatogram.pdf]

Suppl. 2 The total ion chromatogram

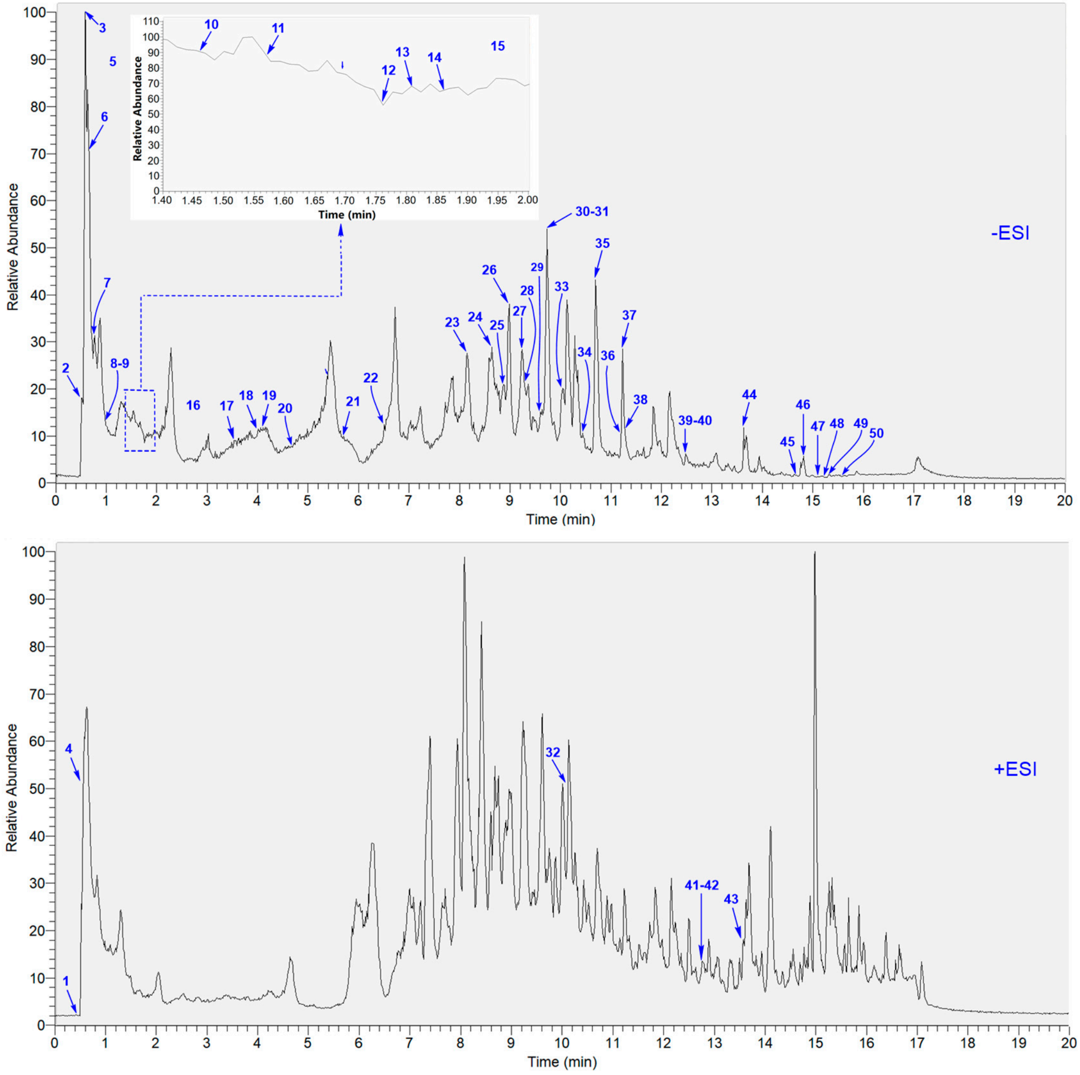

Fig. S2.1 The TIC chromatogram of CoCA was obtained using database-aided UHPLC-Q-orbitrap-MS/MS analysis. The upper section shows the results and further separation of compound 50 for negative ion mode, while the lower section displays the results for positive ion mode.

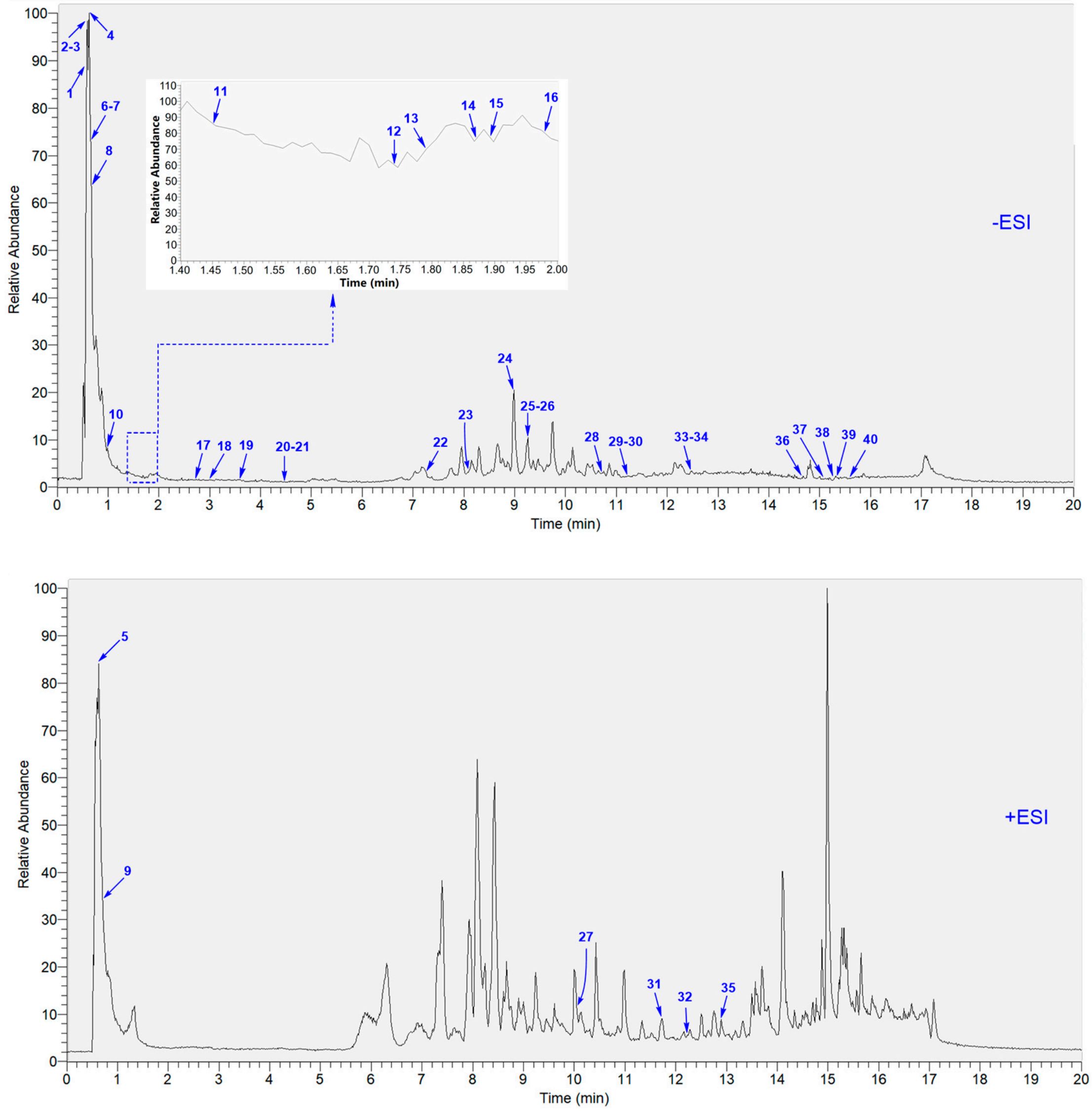

Fig. S2.2 The TIC chromatogram of CoCU was obtained using database-aided UHPLC-Q-Orbitrap-MS/MS analysis. The upper section shows the results and further separation of compound 40 for negative ion mode, while the lower section displays the results for positive ion mode.

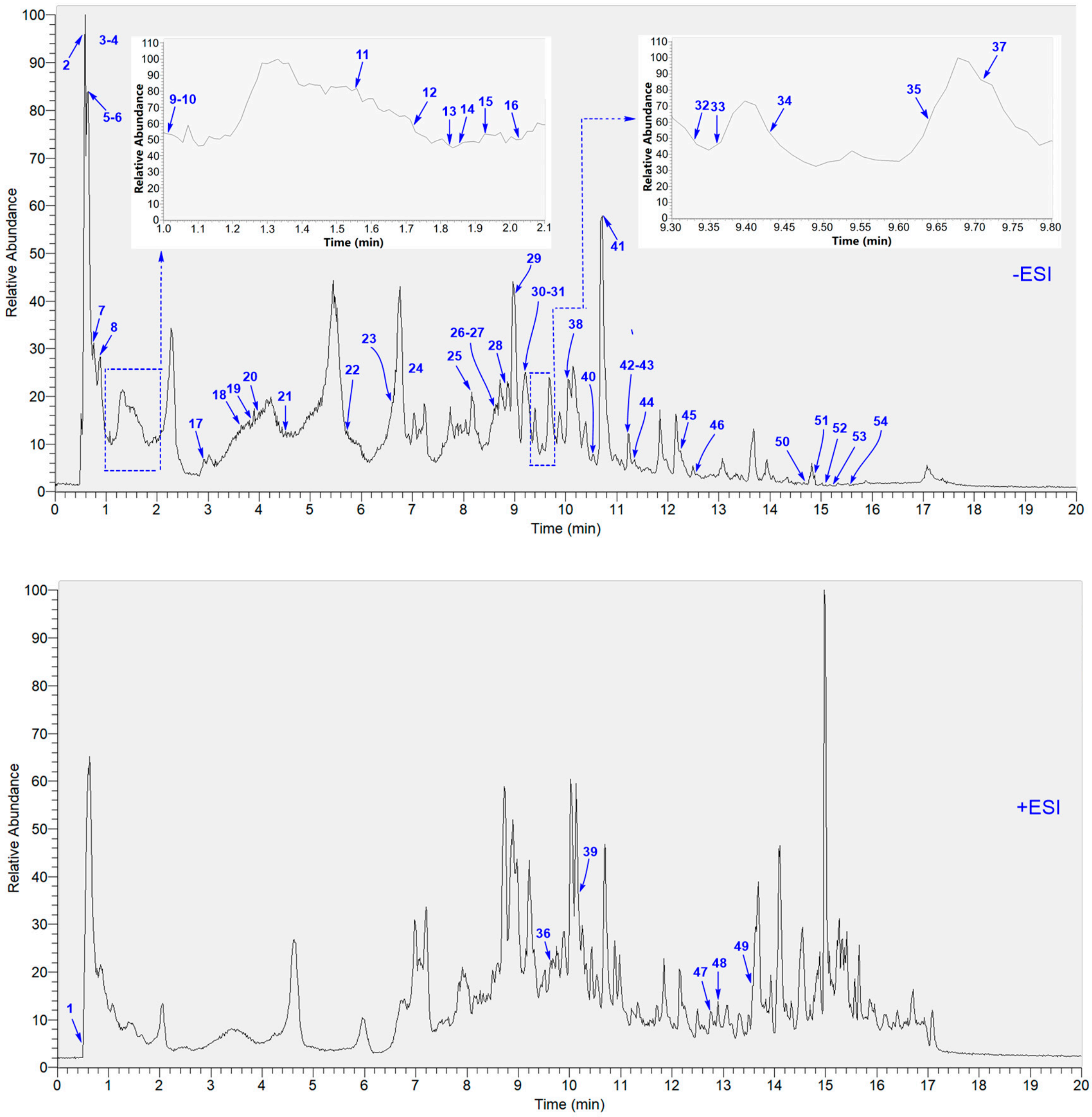

Fig. S2.3 The TIC chromatogram of CoNA was obtained using database-aided UHPLC-Q-orbitrap-MS/MS analysis. The upper section shows the results and further separation of compound 54 for negative ion mode, while the lower section displays the results for positive ion mode.

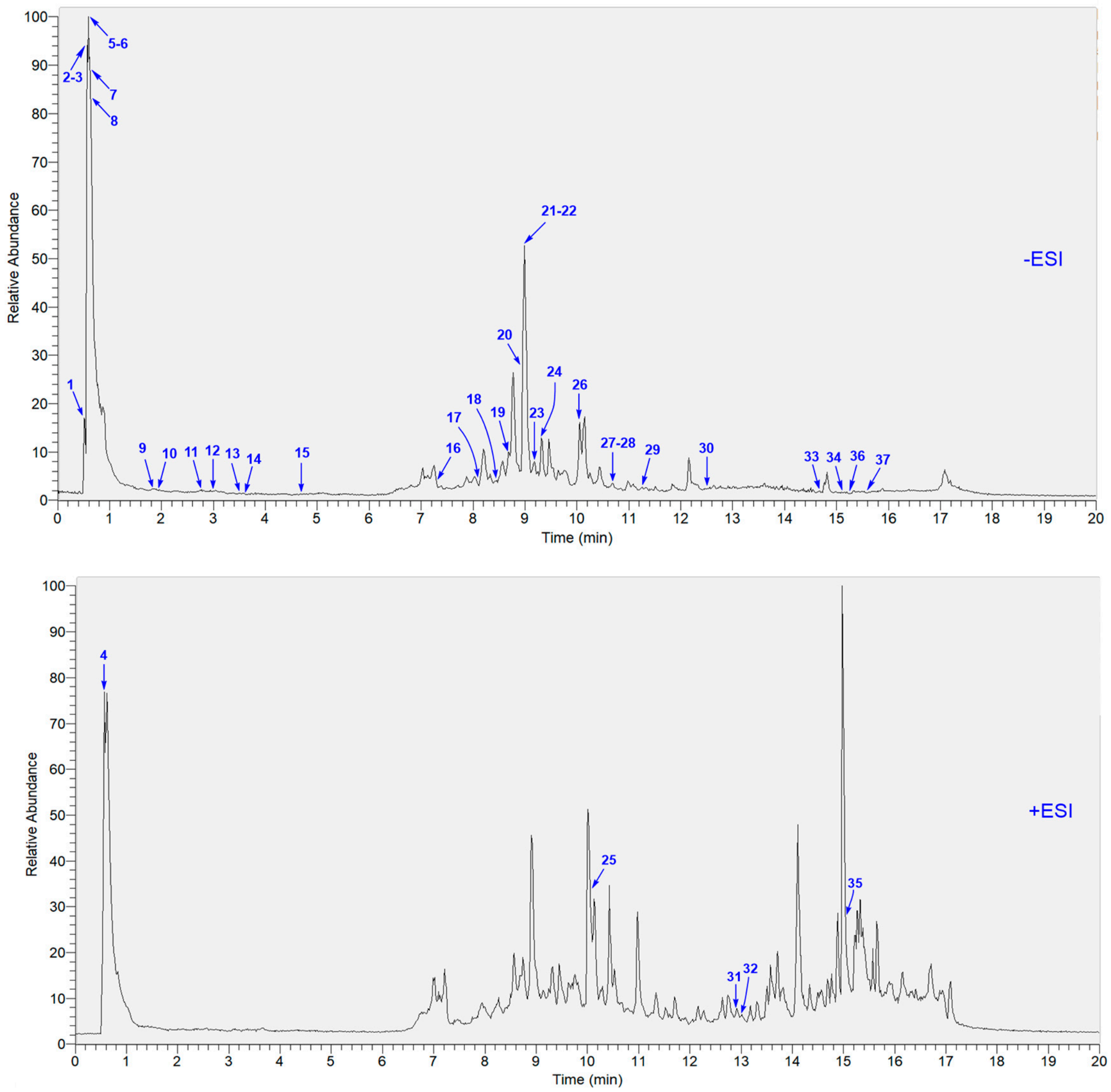

Fig. S2.4 The TIC chromatogram of CoNU was obtained using database-aided UHPLC-Q-orbitrap-MS/MS analysis. The upper section shows the results and further separation of compound 37 for negative ion mode, while the lower section displays the results for positive ion mode.
