# Supplementary material for: Simultaneous Quantification of 66 Compounds in Two Tibetan Codonopsis Species Reveals Four Chemical Features by Database-Enabled UHPLC-Q-Orbitrap-MS/MS Analysis
Source: Molecules. 2024 Nov 3;29(21):5203. doi: 10.3390/molecules29215203 (PMC11547486; doi:10.3390/molecules29215203)
Supplement: Supplementary file 1 [file molecules-29-05203-s001.zip › Supplementary data S4. Measurement of total polyphenols.pdf]

Suppl. 4 Measurement of total polyphenols

Samples	Polyphenols (mg luteolin equiv./g DW)
CoCA	28.38 ± 2.55
CoCU	10.78 ± 0.91
CoNA	57.50 ± 1.35
CoNU	6.72 ± 1.58
