# Supplementary material for: Simultaneous Quantification of 66 Compounds in Two Tibetan Codonopsis Species Reveals Four Chemical Features by Database-Enabled UHPLC-Q-Orbitrap-MS/MS Analysis
Source: Molecules. 2024 Nov 3;29(21):5203. doi: 10.3390/molecules29215203 (PMC11547486; doi:10.3390/molecules29215203)
Supplement: Supplementary file 1 [file molecules-29-05203-s001.zip › Supplementary data S5. Information of all authentic standards.pdf]

Suppl. 5 Information of all authentic standards

Betaine ($C_5H_{11}NO_2$, M.W. 117.15, Cas. 107-43-7, 98%), 1-Kestose ($C_{18}H_{32}O_{16}$, M.W. 504.44 Cas. 470-69-9, 98%), nystose ($C_{24}H_{42}O_{21}$, M.W. 666.58 Cas. 13133-07-8, 98%), quinic acid ($C_7H_{12}O_6$, M.W. 192.17 Cas. 77-95-2, 97%), malic acid ($C_4H_6O_5$, M.W. 134.09 Cas. 6915-15-7, 98%), citric acid ($C_6H_8O_7$, M.W. 192.12 Cas. 77-92-9, 98%), 1,2-Benzenediol ($C_6H_6O_2$, M.W. 110.11 Cas. 120-80-9, 98%), 3,4-dihydroxybenzaldehyde ($C_7H_6O_3$, M.W. 138.12 Cas. 139-85-5, 97%), cis-4-hydroxycinnamic acid ($C_9H_8O_3$, M.W. 164.16 Cas. 4501-31-9, 98%), eriodictyol 7-O-glucoside ($C_{21}H_{22}O_{11}$, M.W. 450.39 Cas. 38965-51-4, 98%), isochlorogenic acid B ($C_{25}H_{24}O_{12}$, M.W. 516.455 Cas. 14534-61-3, 97%), eleutheroside E1 ($C_{28}H_{36}O_{13}$, M.W. 580.6 Cas. 7374-79-0, 98%), apigenin 7-O-glucoside ($C_{21}H_{20}O_{10}$, M.W. 432.381 Cas. 578-74-5, 97%), okanin ($C_{15}H_{12}O_6$, M.W. 288.3 Cas. 484-76-4, 98%), lobetyolin ($C_{20}H_{28}O_8$, M.W. 396.4 Cas. 136085-37-5, 98%), imperatorin ($C_{16}H_{14}O_4$, M.W. 270.284 Cas. 482-44-0, 97%), costunolide ($C_{15}H_{20}O_2$, M.W. 232.32 Cas. 553-21-9, 98%), alantolactone ($C_{15}H_{20}O_2$, M.W. 232.32 Cas. 546-43-0, 98%), zerumbone ($C_{15}H_{22}O$, M.W. 218.33 Cas. 471-05-6, 98%), palmitic acid ($C_{16}H_{32}O_2$, M.W. 256.43 Cas. 57-10-3, 97%), oleic acid ($C_{18}H_{34}O_2$, M.W. 282.46 Cas. 112-80-1, 98%), stearic acid ($C_{18}H_{36}O_2$, M.W. 284.484 Cas. 57-11-4, 97%), α-Linolenic acid ($C_{18}H_{30}O_2$, M.W. 278.43 Cas. 463-40-1, 98%), linoleic acid ($C_{18}H_{32}O_2$, M.W. 280.45 Cas. 60-33-3, 97%) and (9Z,12Z)-N-Benzyloctadeca-9,12-dienamide ($C_{25}H_{39}NO$, M.W. 369.6 Cas. 18286-71-0, 98%) were obtained from Herbest Biotech Co., Ltd (Baoji, China). *D*-gluconic acid ($C_6H_{12}O_7$, M.W. 196.16 Cas. 526-95-4, 97%), resorcine ($C_6H_6O_2$, M.W. 110.11 Cas. 108-46-3, 97%), 3,4-dihydroxy-5-methoxybenzoic acid ($C_8H_8O_5$, M.W. 184.15 Cas. 3934-84-7, 97%) and 3-hydroxybenzoic acid ($C_7H_6O_3$, M.W. 138.12 Cas. 99-06-9, 97%) were obtained from Sigma-Aldrich Co., Ltd. (Shanghai, China). Gallic acid ($C_7H_6O_5$, M.W. 170.12 Cas. 149-91-7, 97%), 5-caffeoylquinic acid ($C_{16}H_{18}O_9$, M.W. 354.31 Cas. 906-33-2, 97%), chlorogenic acid ($C_{16}H_{18}O_9$, M.W. 354.31 Cas. 327-97-9, 97%), caffeic acid ($C_9H_8O_4$, M.W. 180.16 Cas. 331-39-5, 97%), cryptochlorogenic acid ($C_{16}H_{18}O_9$, M.W. 354.31 Cas. 905-99-7, 97%), syringic acid ($C_9H_{10}O_5$, M.W. 198.17 Cas. 530-57-4, 97%), (+) taxifolin ($C_{15}H_{12}O_7$, M.W. 304.25 Cas. 480-18-2, 97%), orientin ($C_{21}H_{20}O_{11}$, M.W. 448.38 Cas. 28608-75-5, 97%), quercetin 3-*O*-*β*-*D*-glucuronide ($C_{21}H_{18}O_{13}$, M.W. 478.36 Cas. 22688-79-5, 97%), luteoloside ($C_{21}H_{20}O_{11}$, M.W. 448.38 Cas. 5373-11-5, 97%), hyperoside ($C_{21}H_{20}O_{12}$, M.W. 464.379 Cas. 482-36-0, 97%), *S*-naringenin-*7-O-β-D*-glucoside ($C_{21}H_{22}O_{10}$, M.W. 434.39 Cas. 529-55-5, 97%), isochlorogenic acid C ($C_{25}H_{24}O_{12}$, M.W. 516.45 Cas. 57378-72-0, 97%), isochlorogenic acid A ($C_{25}H_{24}O_{12}$, M.W. 516.45 Cas. 2450-53-5, 97%), *S*-naringenin ($C_{15}H_{12}O_5$, M.W. 272.25 Cas. 480-41-1, 97%), diosmetin ($C_{16}H_{12}O_6$, M.W. 300.267 Cas. 520-34-3, 97%), apigenin ($C_{15}H_{10}O_5$, M.W. 270.24 Cas. 520-36-5, 97%), chrysoeriol ($C_{16}H_{12}O_6$, M.W. 300.263 Cas. 491-71-4, 97%) and 6-gingerol ($C_{17}H_{26}O_4$, M.W. 294.39 Cas. 23513-14-6, 97%) were obtained from Chengdu Alfa Biotechnology Co., Ltd. (Chengdu, China). *L*-leucine ($C_6H_{13}NO_2$, M.W. 131.17 Cas. 61-90-5, 97%), *L*-phenylalanine ($C_9H_{11}NO_2$, M.W. 165.19 Cas. 63-91-2, 97%) and *L*-proline ($C_5H_9NO_2$, M.W. 115.13 Cas. 147-85-3, 97%) were

obtained from J&K Scientific Co., Ltd. (Beijing, China). Protocatechuic acid($C_7H_6O_4$, M.W. 154.12 Cas. 99-50-3, 97%), vicenin-2 ($C_{27}H_{30}O_{15}$, M.W. 594.52 Cas. 23666-13-9, 97%) and p-Cresol (C_7H_8O, M.W. 108.13 Cas. 106-44-5, 97%) were obtained from SichuanWeikeqiBiologicalTechnologyCo., Ltd. (Chengdu, China). Daphnetin ($C_9H_6O_4$, M.W. 178.14 Cas. 486-35-1, 97%), 5,7-dihydroxychromone ($C_9H_6O_4$, M.W. 178.14 Cas. 31721-94-5, 97%), coniferaldehyde ($C_{10}H_{10}O_3$, M.W. 178.18 Cas. 20649-42-7, 97%), viscidulin I ($C_{15}H_{10}O_7$, M.W. 302.24 Cas. 92519-95-4, 97%), luteolin ($C_{15}H_{10}O_6$, M.W. 286.24 Cas. 491-70-3, 97%) and chrysin ($C_{15}H_{10}O_4$, M.W. 254.24 Cas. 480-40-0, 97%) were obtained from BioBioPha Co., Ltd. (Kunming, China). Sucrose ($C_{12}H_{22}O_{11}$, M.W. 342.3, Cas. 57-50-1, 98%) was obtained from TCI Chemical Co. (Shanghai, China). Ferulic acid ($C_{10}H_{10}O_4$, M.W. 194.19 Cas. 1135-24-6, 97%) and agrimol B ($C_{37}H_{46}O_{12}$, M.W. 696.31 Cas. 55576-66-4, 97%) were obtained from Chinese Medicine Solid State Manufacturing Technology (Nanchang, China). Emodin ($C_{15}H_{10}O_5$, M.W. 270.24 Cas. 518-82-1, 97%), atractylenolide III ($C_{15}H_{20}O_3$, M.W. 248.32 Cas. 73030-71-4, 97%) and 4-hydroxybenzoic acid ($C_7H_6O_3$, M.W. 138.12 Cas. 99-96-7, 97%) was obtained from National Institute for the Control of Pharmaceutical and Biological Products (Beijing, China). All other reagents used in this study were purchased as analytical grade from the ChengDu Chron Chemicals Co,.Ltd (Sichuan, China).
